# Automated flow cytometric identification of disease-specific cells by the ECLIPSE algorithm

**DOI:** 10.1038/s41598-018-29367-w

**Published:** 2018-07-19

**Authors:** Rita Folcarelli, Selma van Staveren, Roel Bouman, Bart Hilvering, Gerjen H. Tinnevelt, Geert Postma, Oscar F. van den Brink, Lutgarde M. C. Buydens, Nienke Vrisekoop, Leo Koenderman, Jeroen J. Jansen

**Affiliations:** 1 Analytical Chemistry, Institute for Molecules and Materials, Radboud University, P.O. Box 9010, 6500 GL Nijmegen, The Netherlands; 2 TI-COAST, Science Park 904, 1098 XH Amsterdam, The Netherlands; 3 Department of Respiratory Medicine and laboratory of translational immunology, University Medical Center Utrecht, Heidelberglaan 100, 3584CX Utrecht, The Netherlands

## Abstract

Multicolor Flow Cytometry (MFC)-based gating allows the selection of cellular (pheno)types based on their unique marker expression. Current manual gating practice is highly subjective and may remove relevant information to preclude discovery of cell populations with specific co-expression of multiple markers. Only multivariate approaches can extract such aspects of cell variability from multi-dimensional MFC data. We describe the novel method ECLIPSE (Elimination of Cells Lying in Patterns Similar to Endogeneity) to identify and characterize aberrant cells present in individuals out of homeostasis. ECLIPSE combines dimensionality reduction by Simultaneous Component Analysis with Kernel Density Estimates. A Difference between Densities (DbD) is used to eliminate cells in responder samples that overlap in marker expression with cells of controls. Thereby, subsequent data analyses focus on the immune response-specific cells, leading to more informative and focused models. To prove the power of ECLIPSE, we applied the method to study two distinct datasets: the *in vivo* neutrophil response induced by systemic endotoxin challenge and in studying the heterogeneous immune-response of asthmatics. ECLIPSE described the well-characterized common response in the LPS challenge insightfully, while identifying slight differences between responders. Also, ECLIPSE enabled characterization of the immune response associated to asthma, where the co-expressions between all markers were used to stratify patients according to disease-specific cell profiles.

## Introduction

Multicolour Flow Cytometry (MFC) is a powerful analytical technique, widely used in biomedicine as a diagnostic tool to evaluate and characterize health and disease^1^. It enables quantitative detection of marker expression, among other cell characteristics, at the single-cell level by specific antibodies conjugated to a multitude of fluorophores. The power of MFC lies in the simultaneous measurement of multiple surface or intra-cellular markers. This allows both a comprehensive biological and physical characterization of cells and cell populations of interest. Advances in technology and fluorophore chemistry have drastically increased the number of parameters that can be concurrently measured^2,3^. Fluorescence-based flow cytometry allows simultaneous measurement of more than 20 markers, while the most recent generation of mass cytometry platforms (Cytometry-Time of Flight) can routinely run experiments with more than 40 parameters^4^. In fact, massive amounts of data are generated in a single experiment, for which many different dedicated data analysis methods have been proposed^5^.

One of the major objectives of MFC data analysis is the identification of homogenous cell types of interest. In the conventional MFC data analysis software, cells of interest are selected through a selection process called ‘gating’, based on uni- or bivariate marker expressions. Manual ‘multiple’ gating on binary combinations of cell characteristics is by far the most widely used method. This is however highly subjective and resource-intensive, because expert technicians need to establish quantitative thresholds in several bi-dimensional plots that cannot be mutually compared on the single-cell level. Manual gating of a data set with seven measured cellular markers would already require inspection of 21 bivariate plots per individual sample. The number of possible combinations becomes difficult to manage with increasing numbers of measured markers, to the extent that the manual gating approach becomes unfeasible quite soon. Aside from the extensive time-consumption involved, it would place additional requirements in consistency of operation and expertise between operators. Moreover, this bi-dimensional approach is done hierarchically, by which cell populations may be overlooked like in sequential gating on single markers^6^.

Recently, several multivariate methods have been proposed to overcome these problems. The viSNE method^7^ is commonly used as a visualization tool for high-dimensional MFC data. Clusters of single cells are visualized in a biaxial viSNE map, using the non-linear t-Stochastic Neighbour Embedding (t-SNE) algorithm for dimensionality reduction. Even though viSNE may be beneficial in the presence of strongly non-linear data, the use of such a non-convex objective algorithm brings about several drawbacks. Each run performed on the same dataset would result in a different map, making the maps difficult to validate. Consequentially and importantly, the arrangement of the cells cannot be directly and easily associated with the marker expression and it is not possible to project a new individual in an existing map without a complete new run. This highly limits the comparison of new, incoming datasets to a model calibrated and validated as a diagnostic instrument for single-cell analysis.

Spanning-tree Progression Analysis of Density-Normalized Events (SPADE)^8^ uses hierarchical clustering to connect different cell subpopulations in ‘minimum spanning trees’ which represents their mutual relations. The cell distribution is visualized as nodes of clustered cells in the SPADE tree that have specific phenotypes. Unlike viSNE, a new MFC sample may be represented into a spanning minimum tree previously built on a dataset, by matching all the cells to the nodes with the most similar phenotype. However, if an extra cell population is present in the new sample, these cells are forced to incorrectly belong to one or more of the available nodes. The (high) residuals of the projection of the cells are not directly detectable. Another recently developed method, Citrus^9^, also uses hierarchical clustering to identify phenotypically similar cell populations. The method is particularly used for intergroup analysis, for which a regularized classification model detects group-specific cell clusters for each sample. Both SPADE and Citrus adopt multivariate and linear approach but they fail, like viSNE, to show the multivariate co-expression that underlies the diversity of cell phenotypes. Co-expression relationships among all the measured markers either remain hidden in the models or are limited to a visual inspection of all the single marker expression levels.

Two alternative methods to analyse Flow Cytometry data are APS (Automatic Population Separator)^10^, and FLOOD (FLow cytometric Orthogonal Orientation for Diagnosis)^11^. Both methods employ Principal Component Analysis (PCA)^12^ as dimension reduction technique to condense the relevant biological cell marker information in a few, mostly two, dimensions. Although they use the same underlying principle, both methods differ considerably in important aspects. APS builds a PCA model on different phenotypes of disease known as reference groups. After that, each case is compared against each reference group^13^ and it is matched to the closest group in the PCA space. The reference groups need to be established *a priori*, which becomes challenging in absence of information about a disease and/or with a small number of individuals within each reference group. The solution to circumvent this disadvantage is to use healthy individuals as reference group and highlight the deviation of diseased individuals from the normal cell marker variability, as done in FLOOD. The FLOOD approach first describes the characteristic cell-to-cell marker variability among healthy (or control) individuals; then it models the cell variability present only in responding individuals within a second model, the response model. FLOOD provides multivariate ‘biplots’ that offer an intuitive view of the cell marker variability associated to a specific immune response, interpreted in terms of co-expression between all the markers, in a representation that is analogous to that used in bivariate gating. However, the information within the FLOOD response model is reduced by the contribution of considerable numbers of ‘healthy’ (or not response-specific) cells in the analysis of responding individuals.

We present ECLIPSE (Elimination of Cells Lying in Pattern Similar to Endogeneity), which combines Simultaneous Component Analysis (SCA), a generalization of PCA suitable for MFC data, and Probability Density Functions of the cell distribution in the dimension reduced space, to identify and eliminate the ‘healthy’ or ‘normal’ cells from the patient (or responder) data. Since these normal cells in the responder individuals are not the discerning factor in the disease response (only the change in number is), they can be removed from the MFC dataset. When initially healthy cells change in relative abundance or when they upregulate or downregulate certain markers as a response to a stimulus or a disease, these cells are considered response-specific and are, therefore, not removed from the dataset.

## Methods

### Data

#### LPS study

MFC measurements of the ‘Lipopolysaccharide study ‘were performed on cells obtained from healthy individuals challenged with systemic endotoxin (LPS). (NCT01374711; www.clinicaltrials.gov) The LPS dataset comprises gated neutrophils from eight ‘control’ individuals who did not receive LPS intravenously, and eight different ‘response’ individuals who received LPS administration and from whom blood was collected 180 minutes post LPS administration. Seven surface markers were measured on the neutrophils in samples of both classes of individuals: CD62L, CD11b, CD11c, CD64, CD32, CD69, and CD16. Further details regarding the Flow Cytometry experiments that generated the data can be found in a previous publication^11^.

#### Asthma study

The asthma dataset consists of a ‘response group’ of 11 patients classified as suffering from severe asthma (aged 22–78, $\bar{{\rm{x}}}=57$) and a ‘control group’ of 10 non-asthmatic individuals (aged 25–57, $\bar{{\rm{x}}}=40$.). Whole-blood cells were stained with the following eight fluorescent markers: CD3, CD4, CD8, CD14, CD16, CRTH2 (CD294), CD123, and CD193. After staining, red blood cells were lysed using a FACS Lysing solution (Becton Dickinson). Cells were measured on a LSR Fortessa flow cytometer (Becton Dickinson)^14^.

In both studies, the data were compensated before the analysis to limit the spectral ‘spill over’ between the markers. This assures that conclusions drawn on marker co-expressions were mainly due to biological correlation rather than spectral overlap.

Details about the pre-processing procedure to transform the data can be found in the Supplementary Material I.

## LIPSE Algorithm

**Figure 1:**
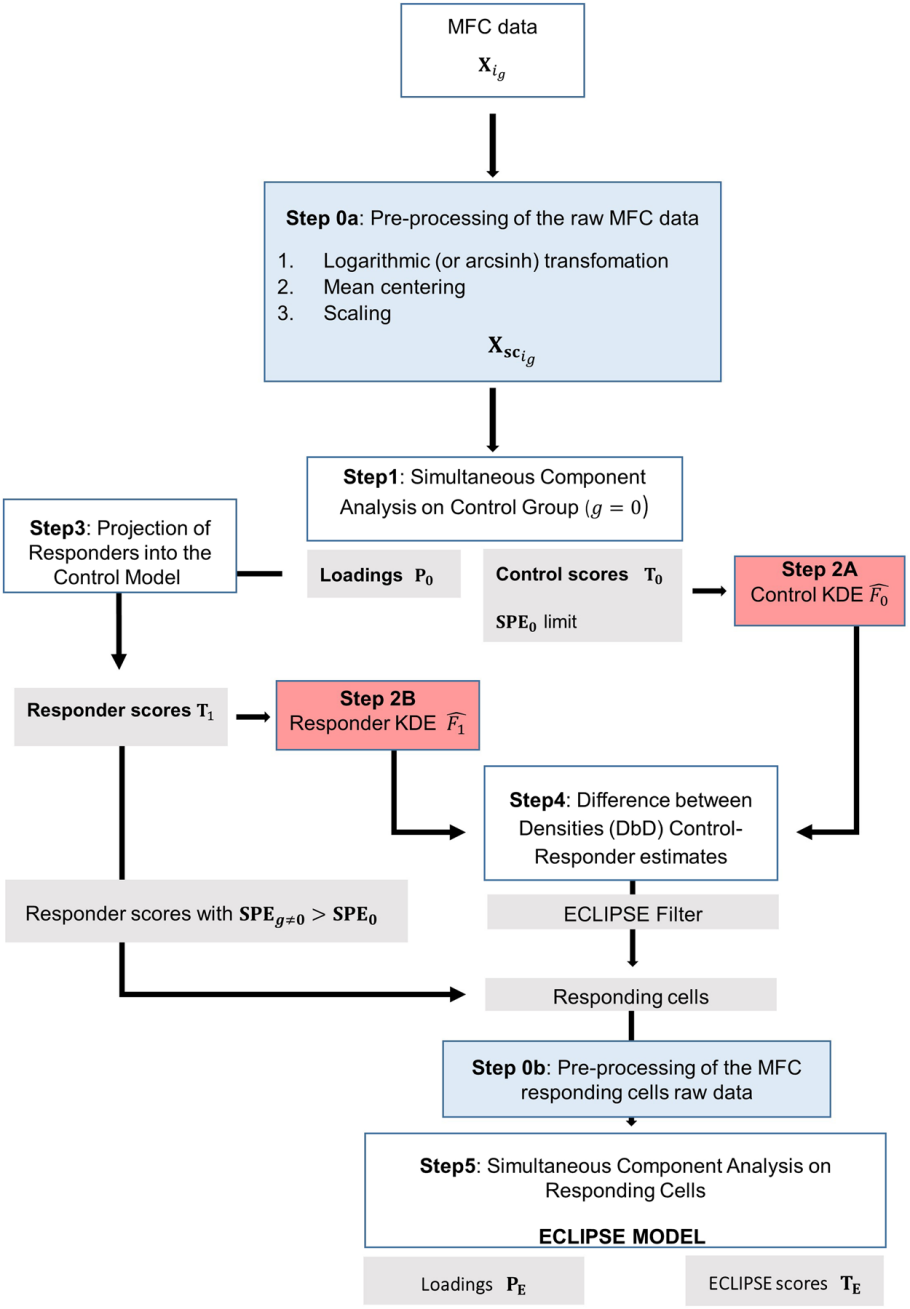
Flowchart of the ECLIPSE algorithm. In light blue are highlighted the pre-processing steps (0a: pre-processing on the raw MFC data, 0b: pre-processing on the raw MFC data, after removal of normal cells); in red the probability densities distribution estimations (2A: estimate of control PDF, 2B: estimate of responder PDF, from the projection of responder-group cells); in grey the results of the corresponding previous steps.

The MFC data were arranged in a multiset structure illustrated in the Supplementary Material I. This arrangement is relevant throughout the quantitative analysis of Flow Cytometry data and each following step of ECLIPSE therefore takes this distinctive structure by design, while retaining the information of each single individual sample. In fact, the ECLIPSE method, through the whole algorithm, takes into account the fact that cells in MFC data may come from different individuals. Difference in terms of the number of cells measured per individual is preventively addressed in the pre-processing (extensively described in Step 0: Data pre-processing, in Supplementary Material I), so that each individual equally contributes to the models, disregarding the size of each set. The ECLIPSE algorithm is described step by step and the FlowChart of the methodology is shown in Fig. 1.

### Step 1: Simultaneous Component Analysis on control individuals

Chemometric dimension reduction techniques such as Principal Component Analysis (PCA) reduce the multidimensional information into a few dimensions to facilitate the information-rich representation of, in this case, flow cytometry data^6^. PCA is an unsupervised technique for the multivariate explorative analysis that operates dimension reduction of multidimensional data. The idea underlying PCA is to contract the information present in the original variables in a lower-dimensional space that best fits the data, with the aim of retaining all the relevant information and removing minor, non-systematic sources of variability. PCA operates a projection of the original data onto a new, reduced space where each dimension is a linear combination of the original variables. The new dimensions, called *Principal Components* (PCs), capture as much variability in all immunological features among all measured MFC events as possible. They are mutually orthogonal and the first Principal Component explains most of the original variance and the successive principal components explain then the highest percentage of remaining variance^12^.

PCA has been widely used to reduce the dimensionality of Multicolour flow cytometry data^10,15,16^. Because these earlier methods disregard the MFC multiset structure (described in Figure S1 of the Supplementary Material I), application of such methods may lead to individuals with large numbers of cells dominating the resulting model. Multiset extensions of PCA, such as described here, consider these differences, in a way that each individual will contribute equally to the constructed model. Secondly, PCA on both control and responder individuals simultaneously may mix normal and response-related cell variability in the resulting PCA model. Both aspects need to be addressed in a comprehensive modelling approach.

From the multiset structure, we consider the control individuals and we build a *Control Model*, which describes the cellular markers’ variability within all the control class individuals. This variability is not of primary interest for describing the immune response; it may therefore serve as a benchmark, where the complement to the cell variability in immunologically perturbed samples or responder individuals is more likely response-related. The availability of identical loading vectors across all control individuals explicitly models the similarities between all control individuals and allows direct comparison between the distinctive scores for each control individual. The Simultaneous Component Analysis with equal patterns (SCA-P) imposes the constraint of identical loading vectors^17^.

The pre-processed MFC data are arranged in the matrix ${{\bf{X}}}_{{{\rm{sc}}}_{0}}$ from Equation S5 (Supplementary Material I, *Data pre-processing*) of size ${N}_{{i}_{g}}\,\times \,J$, where ${N}_{{i}_{g}}$ is the number of cells of the ${i}_{g}$–th individual, ${i}_{g}={1}_{g},\ldots ,{I}_{G}$, with $g=0$ for the control and $g\ge 1$ for the responder groups; $J$ corresponds to the markers measured, $1\ldots j\ldots J$. Before applying the SCA decomposition, ${{\bf{X}}}_{{{\rm{sc}}}_{0}}$ is normalized such that each individual contributes with the same amount of information, regardless of the number of cells measured per set (Eq. 1). The resulting matrix ${{\bf{X}}}_{{{\rm{sc}}}_{{0}^{\ast }}}$ is then decomposed according Eqs 2a,b,c:

1 $${{\bf{X}}}_{{{\rm{sc}}}_{{{\rm{0}}}^{\ast }}}=[\begin{array}{c}{{\bf{X}}}_{{{\rm{sc}}}_{{1}_{0}}}{{\bf{N}}}_{{1}_{0}}^{-1}\,\\ \vdots \\ {{\bf{X}}}_{{{\rm{sc}}}_{{I}_{0}}}{{\bf{N}}}_{{I}_{0}}^{-1}\end{array}]$$

2 $$\begin{array}{llll}(a) & {{\bf{X}}}_{{{\rm{sc}}}_{{0}^{\ast }}} & = & {{\bf{T}}}_{{0}^{\ast }}{{\bf{P}}}_{0}^{{\rm{T}}}+{{\bf{E}}}_{{0}^{\ast }}\\ (b) & {{\bf{T}}}_{0} & = & {{\bf{X}}}_{{{\rm{sc}}}_{0}}{{\bf{P}}}_{0}^{{\rm{T}}}\\ ({\rm{c}}) & {{\bf{E}}}_{0} & = & {{\bf{X}}}_{{{\rm{sc}}}_{{\rm{0}}}}-{{\bf{T}}}_{0}{{\bf{P}}}_{0}^{{\rm{T}}}\end{array}$$

where the matrix of the scores ${{\bf{T}}}_{0}$, of size $\sum _{{i}_{0}}^{{I}_{0}}{N}_{{i}_{g}}\,\times \,{R}_{C}\,\,$, with ${R}_{{\rm{C}}}$ dimensionality of the new low-dimensional space, holds the coordinates of the ${i}_{g}\,-$ th control individual in the new space; ${{\bf{P}}}_{0}$ the $J\,\times \,{R}_{{\rm{C}}}$ matrix of loadings, common to all the control individuals; ${{\bf{E}}}_{0}$ of size $\sum _{{1}_{0}}^{{I}_{0}}{N}_{{i}_{g}}\,\times \,J\,\,$contains the residuals of the ${i}_{g}$ control individuals, with ${i}_{g}={1}_{0},\ldots ,{I}_{0}$.

The scores ${{\bf{T}}}_{0}$ represent the major variability across cells in all cellular markers simultaneously. This is however an inherently simplified representation. The residuals matrix ${{\bf{E}}}_{0}$ contains the cell variability that remains unmodeled by loadings $\,{{\rm{P}}}_{0}$. These residuals may be diagnostic for outlying or abnormal cells, where cells with high residuals are poorly described by the Control loadings. This abnormality is quantified in the Sum Prediction Error (SPE) in Eq. 3:

3 $${\bf{S}}{\bf{P}}{{\bf{E}}}_{{i}_{0}}={\rm{sum}}({{\bf{E}}}_{{i}_{0}}{{\bf{E}}}_{{i}_{0}}^{{\rm{T}}})$$

Where ${{\bf{E}}}_{{i}_{0}}\,$contains the residuals for the ${i}_{0}$–th individual. The statistics for all the ${I}_{0}$ control individual might be collected in the cumulative $${\bf{SP}}{{\bf{E}}}_{0}=[\begin{array}{c}{\bf{SP}}{{\bf{E}}}_{{1}_{0}}\\ \vdots \\ {\bf{SP}}{{\bf{E}}}_{{I}_{0}}\end{array}]\,\,$$ of size $\sum _{1}^{I}{N}_{{i}_{0}}\,\times \,1$. A confidence limit corresponding to the (1 − *α*) percentile may be evaluated for the statistics ${{\rm{SPE}}}_{{i}_{0}}\,$of each individual as the value exceeded by *α*% of the cells. These individual values with α = 0.05 can be collected in the vector $${{\rm{SPE}}}_{{0}_{0.05}}=[\begin{array}{c}SP{E}_{{1}_{{0}_{0.05}}}\\ \vdots \\ SP{E}_{{I}_{{0}_{0.05}}}\end{array}]\,\,$$of size ${I}_{0}\times \,1$. The total confidence limit, is calculated as the mean of the individuals $SP{E}_{{{\boldsymbol{i}}}_{{0}_{0.05}}}\,$and it is used as reference to establish the degree of “normal condition” of the cells.

### Step 2: Probability density estimation of cell variability with KDE

The Control Model may be represented in a multivariate biplot. This two-dimensional representation, obtained by plotting two principal components and superimposing these onto the loadings, allows simultaneous visualisation of the $\sum _{{1}_{0}}^{{I}_{0}}{N}_{{i}_{g}}\,\,$observations and the *J* variables and therefore how these associate.

In the resulting space, the single cell coordinates of an individual are represented as single points. To enable interpretation of the distribution of the cells and the presence of distinctive cell subpopulations we approximate this distribution from the measured single cells by multidimensional density estimation. The probability density function (PDF) may be used to describe the probability that a cellular marker expression assumes a certain value in a D-dimensional space, which can be established by Kernel Density Estimation^18,19^.

This estimate does not require *a priori* assumptions about the shape of the underlying distribution of the expressions, such as Mixture Modeling^20^. The density estimation results from the sum of the individual contributions of the estimated function centered at each sample position. In our case the sample position is represented by the scores’ coordinates in the ${R}_{{\rm{C}}}$- dimensional PCA space for each cells belonging to the ${i}_{g}$–th individual.

Step 2A, in the Flowchart Fig. 1, estimates the PDF of the control cell scores with KDE. Equation 4 contains the formula for the KDE for each cell score belonging to the ${i}_{0}$–th control individual:

4 $$\widehat{{f}_{{i}_{0}}}(t,{\rm{h}})=\frac{1}{{N}_{{i}_{0}}h}\sum _{{1}_{0}}^{{N}_{0}}K(\frac{{{\bf{t}}}^{{\rm{T}}}-{{\bf{t}}}_{{n}_{{i}_{0}}}^{{\rm{T}}}}{h})$$

where ${{\bf{t}}}^{{\bf{T}}}\,$is a vector holding the location where the function is being evaluated, expressed in score coordinates in the PCA space; ${{\bf{t}}}_{{n}_{{i}_{g}}}^{{\rm{T}}}\,$contains the location of the $i$-th cell score belonging to the control individual ${i}_{0}$–th; ${N}_{{i}_{0}}$ is the total number of cells; $K$ the kernel function centered at the cell score location and $h$ the width of the such kernel function, that works as smoothing parameter.

We used Gaussian function as kernel functions $K$. Several studies have shown, however, that this choice of $K$ does not strongly determine the estimate^21^. The kernel bandwidth $h$ in Eq. 4 determines the model outcome to a much larger extent. Figure S2 in the Supplementary Material I, illustrates the effects of the bandwidth choice on the estimate of a normal distribution. Large bandwidths will mask finer structures of the data that may be biologically relevant. Too-small bandwidths may result in spiky density estimates that may be challenging to interpret. Finding the bandwidth where the estimate best approximates the true distribution is therefore imperative. Various methods for optimal bandwidth selection have been proposed in literature^22,23^. The method proposed by Botev *et al*. based on the Fast Gauss Transform performs best in estimating simulated distributions^24,25^. This relatively fast and accurate method was particularly better in representing multimodal distributions of the cellular marker expressions that are characteristic of multiple cell populations. A specific advantage of using the aforementioned method to fit the KDE lies in the fact that it automatically evaluates the optimal bandwidth of Gaussian kernel functions $K$. The KDE formulation (Eq. 4) already includes a normalization over the number of cells per individual ${N}_{{i}_{0}}$, facilitating quantitative comparison between estimates of different individuals within the control class. The KDEs for each individual may be pooled into a KDE_0_ of all control individuals by summing up the individual KDEs and subsequent normalization to unit probability.

### Step 3: Projection of responder individuals onto the Control Model

The Control Model created in Step 1 explains the cellular marker variability of the control individuals. Step 3 consists of orthogonal projection of the cells of the responder individuals into the control space. Assuming the value for the case group $\ne \,0\wedge g=1$, the orthogonal projection is provided by the equation:

5 $${{\bf{T}}}_{1}={{\bf{X}}}_{{{\rm{sc}}}_{1}}{{\bf{P}}}_{0}$$

6 $${{\bf{E}}}_{1}={{\bf{X}}}_{{{\rm{sc}}}_{1}}-{{\bf{T}}}_{1}{{\bf{P}}}_{0}^{{\rm{T}}}$$

Where Eq. 5 is further equivalent:

7 $$[\begin{array}{c}{{\bf{T}}}_{{1}_{1}}\\ \vdots \\ {{\bf{T}}}_{{I}_{1}}\end{array}]=\,[\begin{array}{c}{{\boldsymbol{X}}}_{{{\rm{sc}}}_{{1}_{1}}}\\ \vdots \\ {{\boldsymbol{X}}}_{{{\rm{sc}}}_{{{\boldsymbol{I}}}_{1}}}\end{array}]\,{{\bf{P}}}_{0}$$

${{\bf{T}}}_{1}$ $(\sum _{{i}_{g}={1}_{1}}^{{I}_{1}}{N}_{{i}_{g}}\times {R}_{C})\,\,$is the matrix of the scores for all the ${i}_{1}$–th responder individuals, projected in the Control Space using the orthogonal loadings ${{\bf{P}}}_{0}$ (Eq. 1) from controls and ${{\bf{E}}}_{1}$ $(\sum _{{1}_{1}}^{{I}_{1}}{N}_{{i}_{g}}\,\times \,J)\,$refers to the matrix containing the residuals of the projection. Cells with high residual are outliers in the Control Model and considered abnormal or response-specific. Such cells can be detected by evaluating the Sum Prediction Error (SPE) of the projections using Eq. 3, against the confidence limit for the control calculated as mean of the $SP{E}_{{{\boldsymbol{i}}}_{{0}_{0.05}}}\,$of the ${i}_{0}$–th control individuals. Cells of which the SPE exceeds this limit are considered ‘non-normal’ in their marker expression, as it does not overlap with the variability within the Control model density estimate. These non-normal cells are specifically interesting for the immune response and therefore excluded from the Control Space and they will be reintroduced in the model just after the ECLIPSE filter provided by Step 4, Flowchart Fig. 1).

We estimate the Probability Density Function for the scores of the remaining cells of the responder individual with KDE using Eq. 4 (Step 2B of the Flowchart, Fig. 1). In addition to the amenable representation of the cell scores distributions in a 2D-space, the PDF estimations might allow a direct and easy comparison of cell distribution drawn from different populations.

### Step 4: Difference between Densities

We may now match the KDE of each responding individual against the cumulative control kernel estimate, resulting in a Difference between Densities (DbD) for each responder individual^26^. This step is the core of the ECLIPSE algorithm, with the DbD as an automated filter to exclude those cells with a marker expression that is also found in control individuals. Cells are binned onto the DbD; bins with a positive value contain cells with a higher chance to be found in control individuals. This isolates the cells that are more likely to be found in responders than in control individuals, and therefore likely response-specific together with those cells with a SPE that exceeds the limit observed in the control individuals isolated in Step 3.

### Step 5: ECLIPSE model

As a result from previous steps the new matrix ${{\bf{X}}}_{{{\rm{E}}}_{1}}$ contains the raw signals of the response specific cells while retaining the information of the individuals, which means that the multiset structure is maintained. The resulting matrix needs to be pre-processed following the recipe chosen in Step 0. After log or arcsinh transformation, ${{\bf{X}}}_{{{\rm{E}}}_{1}}\,$is autoscaled using the mean and the standard deviation of each responder individual (i.e. individual centering), according to Eqs S2b and S3c, in the Supplementary Material I. The autoscaled matrix ${{\bf{X}}}_{{{\rm{E}}}_{1}}\,$is normalized over the number of cells of each individual, following Eq. 1, and then decomposed through Simultaneous Component Analysis, according to Eq. 8:

8 $$\begin{array}{llll}({\rm{a}}) & {{\bf{X}}}_{{{\rm{E}}}_{{1}^{\ast }}} & = & {{\bf{T}}}_{{{\rm{E}}}_{{1}^{\ast }}}\,{{\bf{P}}}_{{{\rm{E}}}_{1}}^{{\rm{T}}}+{{\bf{E}}}_{{{\rm{E}}}_{{1}^{\ast }}}\\ ({\rm{b}}) & {{\bf{T}}}_{{{\rm{E}}}_{1}} & = & {{\bf{X}}}_{{{\rm{E}}}_{{1}^{\ast }}}\,{{\bf{P}}}_{{{\rm{E}}}_{1}}^{{\rm{T}}}\\ (c) & {{\bf{E}}}_{{{\rm{E}}}_{1}} & = & \,{{\bf{X}}}_{{{\rm{E}}}_{1}}-{{\bf{T}}}_{{{\rm{E}}}_{1}}\,{{\bf{P}}}_{{{\rm{E}}}_{1}}^{{\rm{T}}}\end{array}$$

where the matrix of scores ${{\bf{T}}}_{{{\rm{E}}}_{1}}$ has size $\sum _{{1}_{1}}^{{I}_{1}}{N}_{E,{i}_{g}}\times {R}_{E},\,\,$with $\sum _{{1}_{1}}^{{I}_{1}}{N}_{E,{i}_{g}}\,$the total number of activated or responding in the responder individuals, and ${R}_{{\rm{E}}}$ the number of dimensions of the new ECLIPSE space in which the majority of the original variability is explained; ${{\bf{P}}}_{{{\rm{E}}}_{1}}\,$the $J\times {R}_{{\rm{E}}}$ matrix holding the loadings, common to all the responder individuals; ${{\bf{E}}}_{{{\rm{E}}}_{1}}$ of size $\sum _{{1}_{1}}^{{I}_{1}}{N}_{E,{i}_{g}}\times {R}_{{\rm{E}}}$ contains the residuals for the ECLIPSE space.

The ECLIPSE model, built on the activated cells within the responder individuals, allows the investigation of the cellular marker variability introduced by the immune response to the disease. This variability is indeed modelled through SCA, which makes it directly comparable across of the responder individuals with the common matrix of loadings ${{\bf{P}}}_{{{\rm{E}}}_{1}}$. The loadings contained in ${{\bf{P}}}_{{{\rm{E}}}_{1}}\,$exclusively describe relations and correlations among cellular markers exhibited by cells that produced or activated in the immune response. These relations are the ones important for the understanding of the pathogenesis and progress of the disease under study. No information about the normal cells is left in this model, because the elimination Step 4 excludes cells with marker expressions similar to those observed in the control individuals.

### Stratified ECLIPSE model

The ECLIPSE model may be very insightful to reveal similar cell distribution patterns across different individuals. This information can then be used to subsequently stratify individuals based on their ECLIPSE scores into responder subgroups. This can be very useful to study immune responses involving subtypes that are either known or unknown, with cell variability both unlike each other and unlike that in the control individuals. Such stratification could be done by visual inspection of the score distributions of ${{\bf{T}}}_{{{\rm{E}}}_{1}}$, perhaps including prior knowledge, but here we propose to cluster the scores of different individuals with an automated algorithm. Cells of each individual are binned into the ECLIPSE space; pairwise distances are calculated between bins across all the responders. The obtained distance matrix is then a measure of (dis)similarity of the score distributions between the individuals. Responders that present cells in the same bins (small distances), express similar cell populations and are therefore allocated to the same cluster. When multiple individuals cluster closely together and away from other individuals, they can be modelled by an ECLIPSE (partial) cluster-specific model dedicated to those individuals to better focus upon their specific cell variability in marker expression of likely response-specific cells.

### Quantitative Validation of the ECLIPSE approach

Steps 1 and 5 (Flowchart Fig. 1) involve a dimension reduction to condense the cellular marker variability within the control individuals (Step 1) and the responder cells (Step 5) respectively into fewer representative dimensions. The number of significant principal components needed for this should be estimated with a bootstrapping procedure to result in a stable model solution^27,28^.

The cells within each individual ${{\bf{X}}}_{{i}_{g}}$ from the multiset structure are bootstrapped and SCA is applied to each new resulting multiset matrix; the stability of the angle between the loading subspaces of the bootstrapped samples should be high to indicate that the SCA components describe relevant and systematic information rather than cells occurring in only one or a few individuals.

## Results

We illustrate ECLIPSE with two case studies. The first investigates the immune response in individuals that intravenously received endotoxin Lipopolysaccharide (LPS) as a model of systemic inflammatory response syndrome (SIRS)^29^.

In the second study, the immune response in peripheral blood of severe asthma patients is examined. Asthma is a chronic and heterogeneous disease, consisting of several disease phenotypes with distinct etiologies and underlying immune responses. Distinguishing these is essential for efficient medical treatment of the relevant phenotypes in the asthma patients^30^.

The running time for both the LPS data (281505 events) and the asthma study data (2157326 events) did not exceed 2 minutes when computing the analyses on a laptop with 2.7 GHz, i7-7500U processor, 8GB of memory.

### Lipopolysaccharide challenge

Intravenous administration of LPS in humans induces the rise of neutrophil subpopulations in the peripheral blood that are normally absent. This is most prominent 180 minutes after LPS administration. The two neutrophil subpopulations that are induced after LPS challenge can be distinguished based on their expression of CD16 and CD62L. One of the populations is CD62L^dim^CD16^bright^ and the second population is CD62L^bright^CD16^dim^. Next to these markers it has been shown that co-expression of multiple other surface markers characterize these neutrophil subpopulations^11,31^.

After log-transformation, the data were mean-centred per individual and scaled over the control group, according to Equations S2b and S5b, respectively. Supplementary Material I contains more detailed information on the data pre-processing. A control individual identified as outlier from previous analysis^11^ was excluded from the ECLIPSE modelling.

The bootstrapping procedure indicated that two components for the Control Model describe the variation of the marker expression in a stable model of the Control individuals. These components together explain 45% of variability in marker expression across cells from control individuals, ranging between 42–53% per individual. This indicates that the model provides a balanced representation of all seven control individuals. Although this percentage might appear nominally low because the majority of marker variability remains unexplained, statistical model validation shows that models with additional components are more unstable and therefore inappropriate as generic filters for ‘normal’ cells.

**Figure 2:**
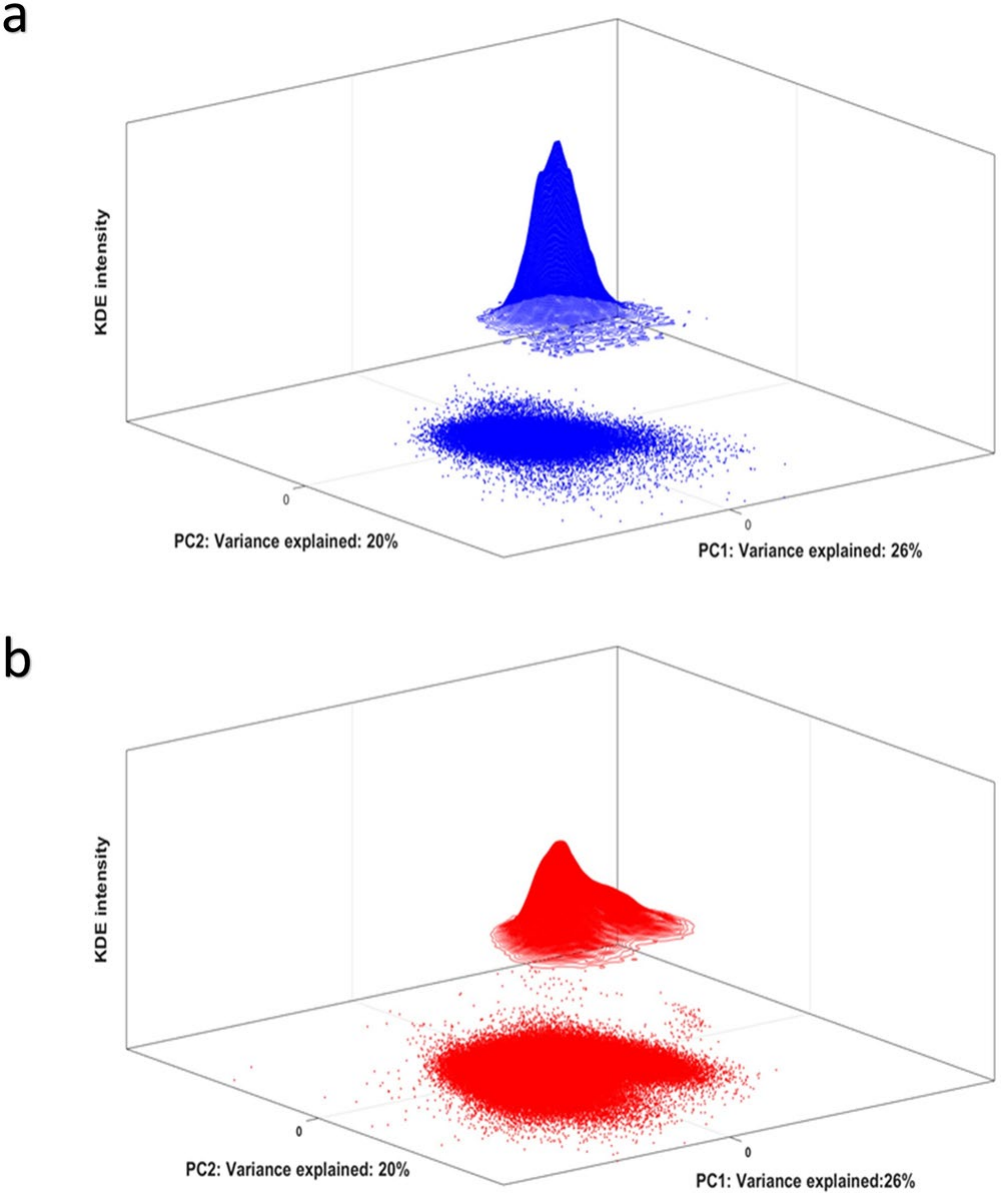
Panel a Scores plot of control cells of all the control individuals in the Control Space, plus 3D-contour of the PDF of the control scores estimated with KDE; Panel b Scores plot of all the responder cells projected in the Control Space, plus 3D-contour of the PDF of the responder scores estimated with KDE.

Figure 2 shows the Control score distributions of cells in control individuals (a, in blue) and of the responder individuals (b, in red) projected onto the control model. Dots represent single cells within the dimension-reduced space. The third dimension is the ‘Kernel density estimation (KDE) intensity’, i.e. the local density estimate at each cell score position. Locations with higher KDE intensities are more densely populated by cells. The inhomogeneous estimate in B indicates a distribution of cells with a continuum variation of fluorescent signal intensities, i.e. surface marker expressions, compared to the control individuals.

**Figure 3:**
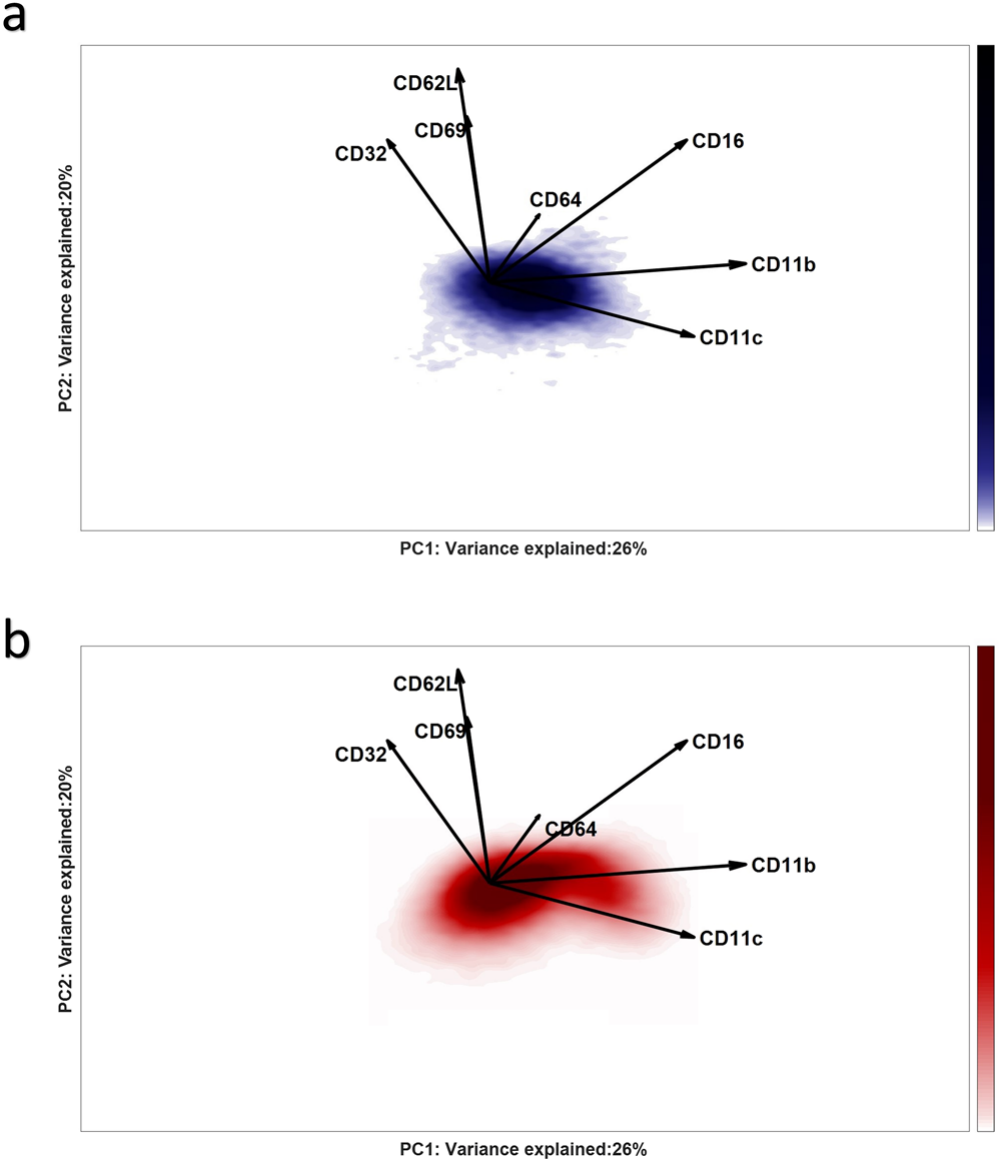
Panel a, Biplot representation of PDF of the control scores, estimated with KDE; Panel b, Biplot representation of the PDF of the responder scores, estimated with KDE. The loadings of the SCA Control Model are plotted as vectors: their length indicates the contribution of each marker to the cell-to-cell variability; the mutual directions suggest a positive (same direction) or negative (opposite direction) co-expression. An angle of 90 between the direction of the vectors indicates no co-expression.

The biplot (Fig. 3a,b) shows the scores distribution of the cells, as a 2D-density representation, together with the surface marker loadings. This plot shows how the continuum of the cell scores associates with the co-expression of the different surface markers. The homogeneous neutrophil population in the control individuals (Fig. 3a) varies mostly in expression of CD11b, CD11c and partially CD16, where cells on the right side of the distribution co-express these markers above-average and more than cells on the left side. The large loadings of CD32, CD62L and CD69, in the vertical direction, indicate that the expression of these markers varies considerably among the neutrophils, along the second component.

The responder cells projected into the Control Model (Fig. 3b) vary in a characteristic ‘bean-like’ shape, showing neutrophil subpopulations towards the markers CD16/CD11b/CD11c from one side and CD32/CD62L/CD69 from the other, that emerge upon LPS response. However, responder individuals contain a considerable fraction of cells with cell surface marker expressions identical to the cells within samples from non-responding Control individuals. These cells are less-interesting for the immune response characterization and would considerably affect any quantitative models of cells variability in responder individuals, at the expense of the rarer cells associated with the LPS induced immune response.

We therefore selected those cells that deviate from the normal cell surface marker variability in the space defined by the first two PCs of the Control model. These abnormal cells are selected in two independent steps.

First, cells with high Sum Prediction Error (SPE), analogous to Eq. 3, are worth further investigation. The Control Model cannot explain these cells well: they exceed the limit established for the Control Model, and are therefore designated as ‘abnormal’. The percentage of these abnormal cells ranges between 12–21% for the different responder individuals—considerably and consistently higher than the 5% of cells in control individuals.

**Figure 4:**
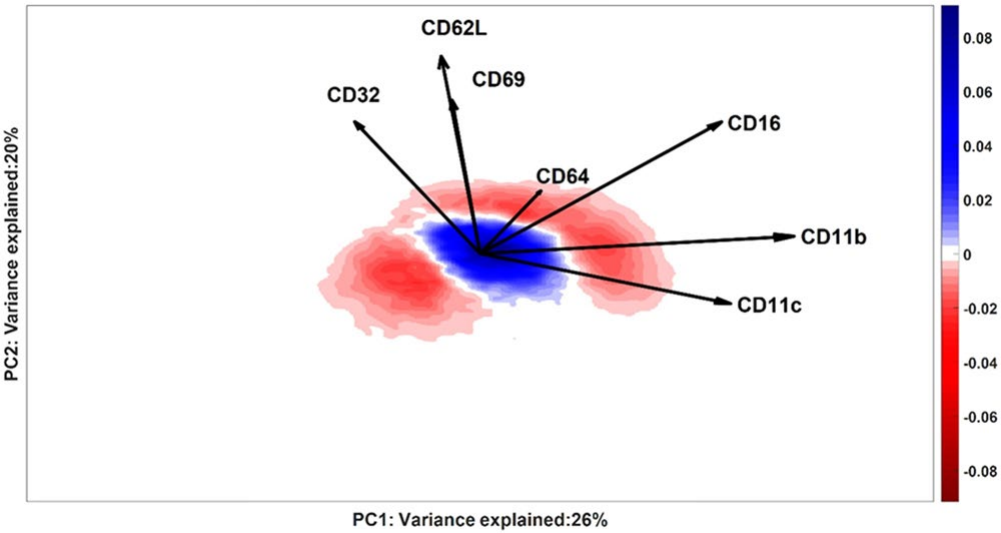
KDE of the scores distribution of a typical LPS-responder (ID #9) is subtracted from the cumulative KDE of the control scores distribution. The negative intensity (red) indicates the location where cells over-produced in the responder are more likely to be present; the positive intensity (blue) indicates the location where healthy cells are more likely to be present. The white area between the red and blue areas corresponds to a value of KDE = 0, which can indicate bins with no cells or equal intensity of control and responder estimates. The axes report the variance of ID#9 explained by the respective PCs (Control Model).

Secondly, cells with model residuals similar to those in the control individuals may be further filtered by quantitative comparison of the kernel density estimates of each Responder against that of the control group. The resulting responder-specific Difference between Densities (DbD) for a typical responder is shown in Fig. 4. Regions with negative intensity contain cells with a higher chance to be found in a responder than in the control individuals on the basis of their Control Model score. These cells are therefore potentially response-related. Those cells lying in regions with positive intensity may be found with higher probability in control than in responder individuals and are therefore excluded from subsequent analyses.

A dedicated new SCA model was modeled on those cells that were either identified based on SPE or DbD as response-related, to specifically fit the variability in marker expression of these LPS-induced cells.

**Figure 5:**
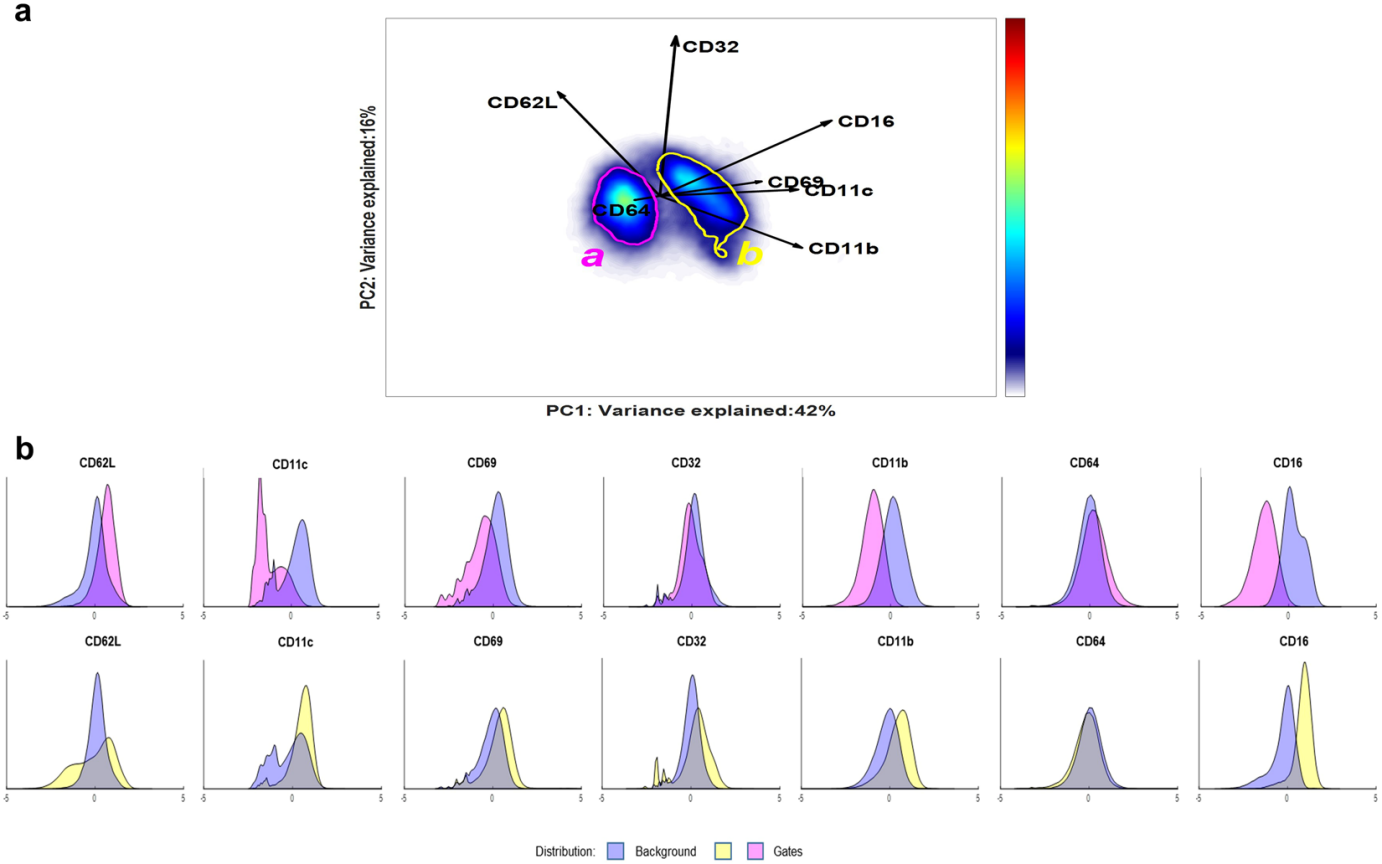
Panel a, ECLIPSE model built on all the responding cells from the responder individuals. The cells generally attributed to the control group have been eliminated from the analysis, and the SCA space has been recomputed. The loadings show the LPS-response specific surface marker expression for all cells. Gates a (magenta) and b (yellow) encircle the two most densely populated areas; Panel b, Histograms of the surface marker expression of the cells lying in the two different gates. The distributions are displayed with the colour of the corresponding gate (magenta for gate a, yellow for gate b); the blue distribution corresponds to the cells not included in the specified gate.

The ECLIPSE Model computed on the response-related cells that were identified before (Fig. 5) shows how two groups of neutrophils arise in the LPS-induced immune response.

The loadings, superimposed on the space, indicate how the surface markers in both groups of cells differ in expression. CD62L is more strongly expressed in gate a, and CD69, CD16, CD11c, and CD11b are more intensively co-expressed on the cells in gate b. Although CD32 varies much in expression across response-related cells, its intensity does not significantly differ between both cell groups.

The population with higher expression of CD62L and lower expression of CD16, in gate a, corresponds to the premature neutrophils (cells with a banded nucleus) and the cells with higher expression of CD16 are mature neutrophils, which were both identified in earlier studies^31^. Single-marker expression profiles of the cells present in both gates are shown as histograms in Fig. 5b, against a background containing the cells that are not included in the specified gate. The background contains both cells of the responders as well as cells of controls outside of the depicted gate. This is analogous to the histogram representation used in Citrus. A differential expression of the marker CD62L, indicated also by the direction of the loading, is present in gate b. This suggests the presence of two sub-clusters within the gate: a cluster which is CD62L^bright^ and a CD62L^dim^ cluster. These sub-clusters might occur due to variability between the different responder individuals. The density estimates of the ECLIPSE cell scores of two different responders (Figure S3, Supplementary Material II) confirm that there is heterogeneity in the surface marker expressions of the cells specifically induced by LPS. The variability in the continuum of surface marker expressions for all the responders are shown in the Table S2 of the Supplementary Material II. All responder individuals react with increases in premature neutrophils three hours after LPS was administered, while the presence and expression profiles of the mature neutrophils vary between individuals. In some cases, these cells consist of two subpopulations. The diversity in responses to the administered LPS is enhanced after the elimination of normal cells. Especially when the number of response-specific cells is limited or when the response is heterogeneous, these normal cells will considerably affect quantitative models of the responder individuals, at the expense of the rarer cells associated to the immune response. As proof of concept, we performed a simultaneous component analysis on the original cells of the responder individuals and compared the model against the SCA Control Model and the SCA ECLIPSE Model, defined previously. The angle between the subspaces spanned by the loadings of each model, built with the first two Principal Components, is used as metric of dissimilarity. A procedure similar to the procedure as described by Liu and al^27^ gives the distribution of the angles between the subspaces. The smaller angle distribution in Figure S4 (Supplementary Material II) confirms that a model built on all cells of the responders grasps a variability in surface marker expression similar to that of the control individuals. The larger angle difference between the ECLIPSE SCA model and Control or Response SCA model shows that the elimination step is essential to capture the surface marker expressions specifically related to the response.

To compare the ECLIPSE results with other multivariate methods, we performed viSNE and Citrus analyses on the same dataset. The cell phenotypes found by the three methods can be compared. From this, we can conclude that the cell phenotypes, highlighted in the ECLIPSE space, are similar to the ones found by the Citrus and viSNE analyses in Supplementary Material III. We performed Citrus and viSNE analyses on three differently pre-processed LPS datasets: the arcsinh transformed (cofactor) LPS data, the LPS dataset pre-processed as used in ECLIPSE (log transformation, mean-centering per individuals, scaling over controls) and the LPS dataset after removal of normal cells by ECLIPSE. When trained on the arcsinh trasformed LPS data, the two most relevant clusters for the classification detected by Citrus may be identified as premature and mature neutrophils (Figure S6). In contrast to the ECLIPSE results, the Citrus cluster of mature neutrophils only consists of CD62L low cells. The cross-validation error rate of the model around 7% (Figure S5) however suggests that the identified clusters might not be specific for all the individuals within the control or responder groups.

Contrary to the arcsinh transformed data, the multi-set data pre-processed according to Equations S2b and S5b led to a perfect classification with Citrus (Figure S7). The similar pre-processing also allows a more direct comparison between both methods. In this case, Citrus recognized two clusters in the LPS data that are more abundant in the control group, with phenotypes that may be assigned to normal mature neutrophils, with CD16+CD62L+CD64− and CD16+CD62L+CD64dim phenotypes, respectively (Figure S8). Only a single cluster, identified as a premature neutrophil population (based on CD16^dim^ marker expression), was found to be distinctive for the response group.

In the third analysis, which was performed on the LPS data after removal of normal cells, only a cluster of premature neutrophils was detected (Figure S10). The premature neutrophil population, which was more abundant in the response group, suffices for a correct classification (Figure S9). In this analysis, there were no clusters of normal mature neutrophils identified as most discriminant ones.

When performing the viSNE analysis on the arcsinh transformed (cofactor 5) LPS data, no clear subpopulations were found. The viSNE map shows single cells with distinctive expression of CD11b, CD11c, CD16, CD32 and CD62L, and the other markers in lesser extend (Figure S11).

Additionally, it seems that cells of the same individual are grouped together, suggesting that the cell distribution found by the algorithm are individual-specific. This grouping is removed when viSNE is performed on the pre-processed data. In the resulting map the cells are indeed evenly distributed across the space and no donor-specific clusters are present (Figure S13).

When the analysis was performed on the data after removal of normal cells, roughly three distinctive cell phenotypes were found based on CD16/CD62L expression: CD16-CD62L+, CD16+CD62L- and CD16+CD62L+ cells (Figure S14). The expressions of the other markers within these three cell populations are more easily interpreted after ECLIPSE-based filtering. Removing the normal cells results in a less crowded viSNE map, which helps identifying the cells of interest and focusing on more subtle differences between and within these cell populations.

### Asthma-specific immune responses

Asthma is a chronic inflammatory respiratory disease that affects the airways. Symptoms range from wheezing and coughing to bronchospasms and dyspnoea. Symptom severity differs among patients, with some barely experiencing problems in daily life, and others having frequent exacerbations, requiring hospitalization^30^. We show here how ECLIPSE may be used to model the asthma-specific immune response associated to differential expression of immune markers on white blood cells (WBCs).

After arcsinh transformation (cofactor 5), the data were mean-centred and scaled over the controls according to Equations S2b and S5b (Supplementary Material I contains more detailed information on the data pre-processing), respectively.

**Figure 6:**
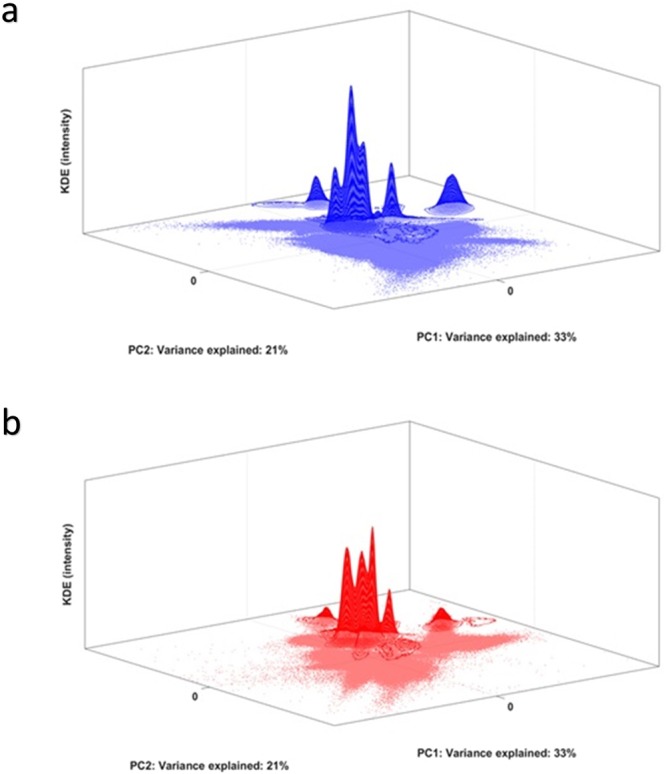
Panel a Scores plot of control cells of all the control individuals in the Control Space, plus 3D-contour of the PDF of the control scores estimated with KDE; Panel b Scores plot of all the responder cells projected in the Control Space, plus 3D-contour of the PDF of the responder scores estimated with KDE. The kernel estimates display PDFs with multiple maxima; this suggests that multiple cell-subpopulations are present in the data.

As a result of the validation step, we used a two-component Control Model for this, the PCs of which explain 33% and 21%, respectively, of the variability in surface marker expression of the 10 control individuals. Figure 6a,b shows the scores and the resulting kernel estimates of control individuals and patients.

The large number of cells and the considerable differences in expression between the cells make the score distributions of patients and controls complex and difficult to interpret, although they are visually distinguishable. The complexity is due to the considerably larger heterogeneity in surface marker expression that can be expected in the full WBC compartment, compared to the gated neutrophils of LPS. In addition, the effects on the immune system due to asthma may be more subtle than the systemic response to LPS. Transcending the single-cell level into the kernel probability density estimates, shows how the multiple cell populations result in multimodal kernel estimates, both in the control and patient group. The high maxima in the center of both control space plots correspond to the neutrophils, the largest constituent of all white blood cells. In addition to the changes in the neutrophils upon asthma immune response, comparing both KDEs already shows that the response involves several other WBC sub-populations as well.

**Figure 7:**
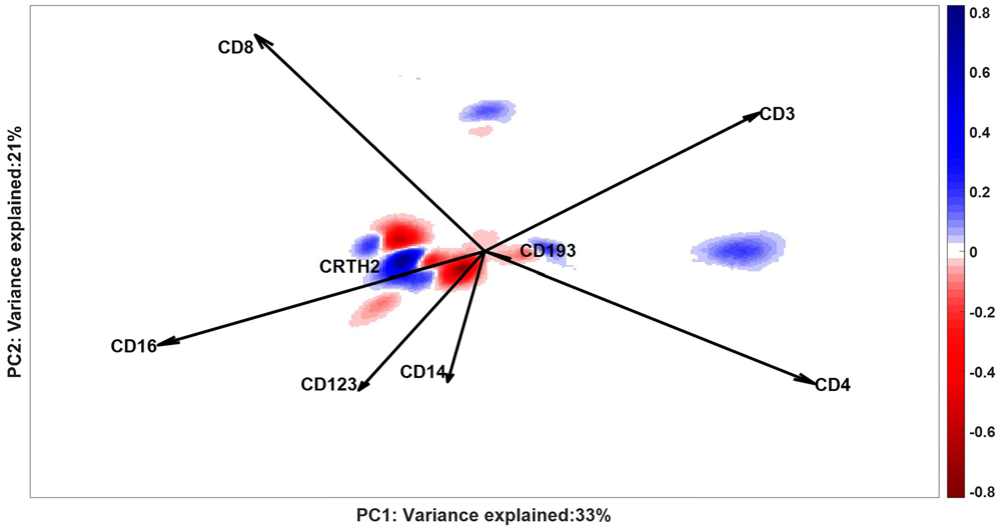
Density plot of Difference between Densities: the negative intensity (red) shows the locations where a larger fraction of cells scores for patients than for control individuals; positive intensities (blue) contain cells with larger fractions of normal cells. White areas either contain no cells or have similar fractions in both groups of individuals. The axes report the variance explained by the SCA Control Model for the control individuals, on which the DbD is calculated. Loadings of the SCA Control Model are represented as vectors: loadings with similar directions indicate positive co-expression (like for CD16, CRTH2 and CD193, CD4) and with opposite directions indicate negative (like for CD3 and CD16) co-expression of both surface markers. Orthogonal vectors indicate surface markers that express independently, e.g.CD8 and CD123.

The DbD plot (Fig. 7) shows which cell populations are over- or underrepresent in patients with severe asthma, when compared to non-asthmatic controls. The DbD plot displays several negative regions (red) with a higher chance of cells with a surface marker co-expression typical of patients; cells in positive areas (blue) are more probable to be found in control samples.

**Figure 8:**
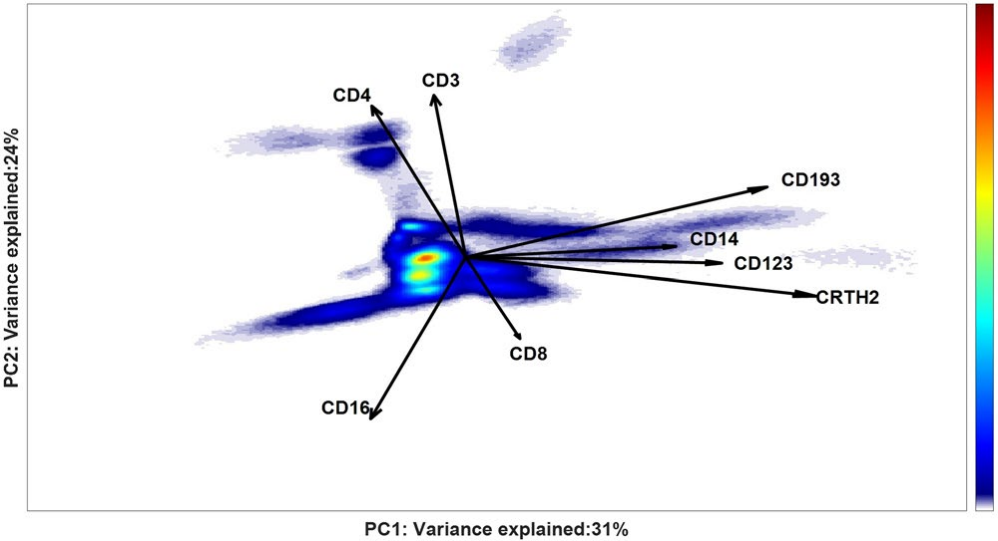
General ECLIPSE model built on all non-normal cells of the 11 asthma patients. A density estimation of the cell scores distribution is shown, where density is quantified from low (blue) to high intensity (red). Multiple cell sub-populations with different intensities can be observed, mainly differing in the co-expressions of CD193, CD14, CD123, CRTH2 (along the horizontal axis), and by simultaneous positive co-expression of CD4, CD3 with a negative co-expression of CD16 (on the vertical axis). Both axes report the variance explained by each component of the two PCs in the SCA model for all the patients. The loading vectors greatly differ from the loadings of the Control Model because they explain the variability of only the not-normal cells.

Cells within negative regions, together with the cells with high residuals are then selected for further analysis within a response-specific ECLIPSE model. The original compensated data of these cells, after arcsinh transformation (cofactor 5), are mean-centered and scaled across all the individuals (Eqs S2-S3, Supplementary Material I). The ECLIPSE algorithm models the variability of all the cells particularly found in the patients, as shown in Fig. 8.

**Figure 9:**
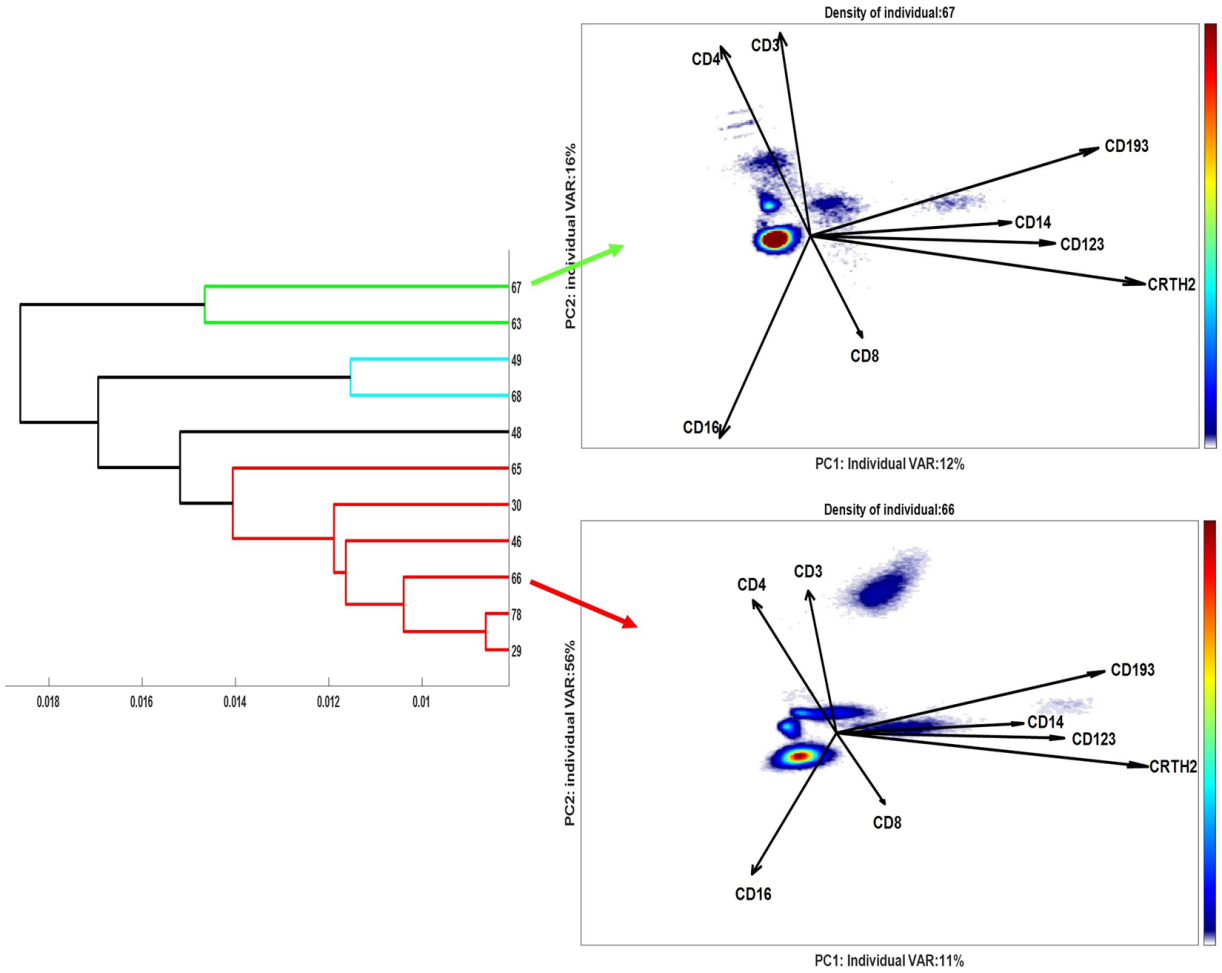
Dendrogram (left panel) of the asthma patients, obtained by average linkage to the distances between the Kernel Density estimates of their ECLIPSE scores. Four different patient clusters are coloured as different dendrogram leaves. The figures on the right panel show the density cells scores distribution of two representative individuals (#67 and #66) from different patient clusters.

From this ECLIPSE model, scores of each individual asthma patient in the ECLIPSE space can be individually extracted and inspected (Table S3, Supplementary Material II, which shows the scores in the general models for all patients). The output of this general ECLIPSE model may be used as input for a clustering analysis, to stratify individuals in an automated and reproducible fashion. Hierarchical clustering by average linkage to the distance of the kernel estimates provides a dendrogram of the patients, visualized in Fig. 9 (left panel), which shows clear subgrouping of this small cohort of asthma patients. Asthma patients may be grouped into three or four clusters, specifically three containing multiple patients (blue, green and red) and one single responder in a separate cluster (black).

**Figure 10:**
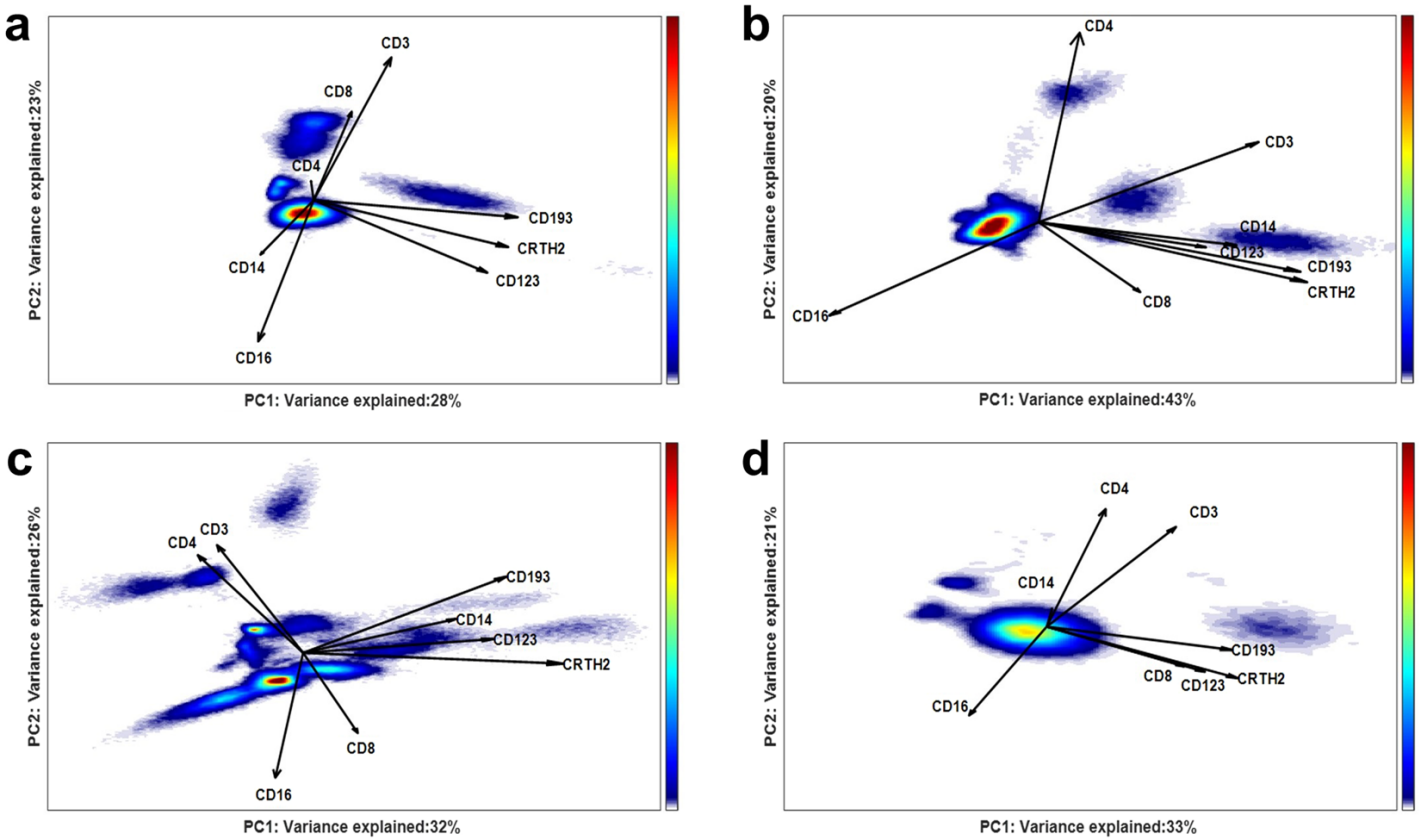
Partial cluster-specific ECLIPSE response models. a Density estimation of cell scores distribution of patients #63 and 67 grouped in the green cluster; b Density estimation of cell scores distribution of patients #49 and 68 grouped in the blue cluster; c Density estimation of cell scores distribution of patients #65, 30, 46, 66, 78 and 29 grouped in the red cluster; d Density estimation of cell scores distribution of patient #48 (black individual). Both axes report the variance explained in total for the patients of each group by the respective PCs of the partial cluster-specific ECLIPSE Models. The 4 models are qualitatively comparable in terms of percentage of variance explained. However, great difference in the loadings indicates that the type of variation accounted by each model is non-identical: each patient cluster is defined by a different and characteristic combination of cell populations.

Figure 9, right panel, shows the scores in the ECLIPSE general model of two patients grouped in different clusters (green and red) and thus reveals differences in the cell populations present. However, the variance in marker expression of the individual patients was not always well described by the general ECLIPSE model: only 28% of the total variance of responder ID #67 was explained in this way. To investigate the cell variability in surface marker expression that is relevant for a specific cluster, we built four partial cluster-specific ECLIPSE models, on each separate cluster of individuals (Fig. 10). As expected for a heterogeneous immune response, the length and the orientation of the loadings are different between the 4 models.

**Figure 11:**
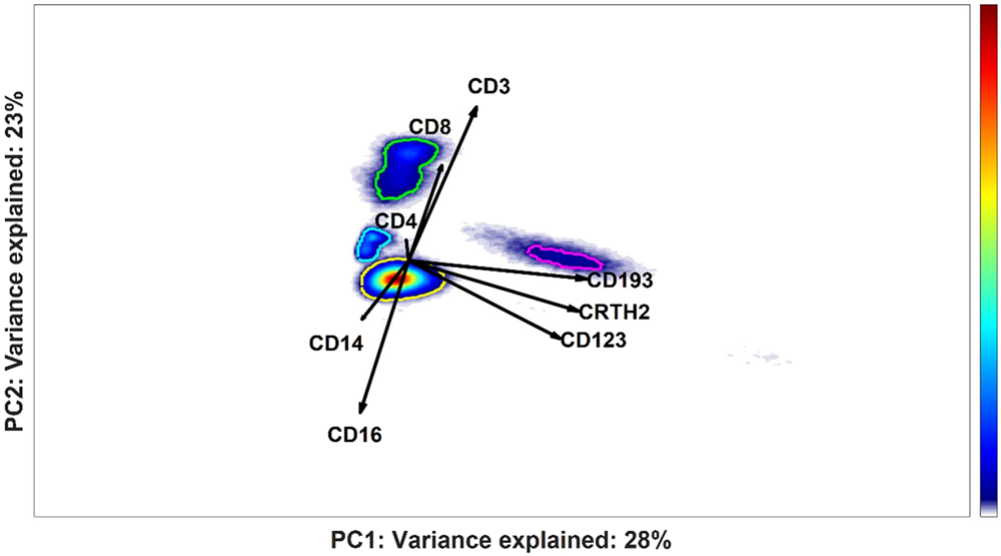
Partial cluster-specific ECLIPSE model built on the variability of the not-normal cells of individual #63 and #67 (green cluster in Fig. 8). Density estimates of cell score distributions of both patients are shown. The intensity of the estimates is indicated with the colour bar on the right, from low (blue) to high intensity (red). The most densely populated regions are highlighted with coloured gates.

The most densely populated regions of the partial ECLISPE models can be automatically gated; the results for the partial ECLISPE model of individuals #63 and #67 from the green cluster are shown in Fig. 11. One of the cell populations (gated in green) is identified as CD8+ T cells, due to its co-expression of markers CD3 and CD8. The partial ECLISPE models built on the other clusters do not show a predominant CD3+ CD8+ T-cell population, in the first two PCs (Fig. 10). On the contrary, in the partial models of the blue and the red clusters CD3+ CD4+ T-cells had a more important contribution to the variance, as indicated by the long loadings in these models and shown in the Supplementary Material IV for the cells of the red clusters. Another characteristic population that we observed in multiple patient clusters most likely were basophils, as indicated by a relatively high expression of CD123, CD193 and CRTH2 (Fig. 11, magenta gate). These latter cells were discriminatory only in some phenotypes of asthma. The mechanism behind the variation in recruitment of cellular populations to the peripheral blood in severe asthma patients will be examined in future studies and goes beyond the scope of this article.

For comparison, we performed Citrus and viSNE on the asthma dataset (Supplementary Material III).

The considerable heterogeneity associated with the asthma study results in a reduced classification accuracy in the Citrus analysis. In fact, the highest achieved accuracy corresponded to 25% of misclassified samples (Figure S15). This is obtained with a model which identifies the four most discriminant cell clusters. Two of these clusters were distinctive for the control group, with CD16+ CD123dimCD8dim and CD3−CD14− expression levels and CD4+ T cell phenotype (CD3+CD4+), respectively. The over-representation of these cells in the control individuals, against the asthma group, was highlighted also by the Difference between Densities plot (Fig. 7). The blue regions here show, among other cell populations, the presence of CD4+ T cells and CD16+CD3− cell, which are more likely to be found in the control individuals. After the removal of normal cells, however, we could observe the presence of CD4+ T cells also in the partial ECLIPSE model of red and blue clusters (Fig. 10b,c). The patients belonging to these clusters, identified by ECLIPSE, might have CD4+ T cells with a differential markers expression compared to the controls, which is emphasized by the difference in the angles of the loadings in the more focused models. Both other clusters identified by Citrus were more represented in the asthmatic patients (Figure S17). In this case, it was harder to identify clear phenotypes, mainly because of very heterogeneous expression patterns. In fact, multiple peaks per marker suggested the presence of multiple cell subtypes within a same cluster. Unfortunately, we were unable to identify which cell types are present in this cluster, since the information about the co-expression of markers is lacking in the Citrus results.

The viSNE map obtained by the analysis of the asthma data showed substantial overlap of cells between the control and the asthmatic patients (Figure S18). Most overlapping regions are associated to CD4+ T cells; CD8+ T cells; CD16+ cells, which might be identified as neutrophils; and CD14+ cells, which might be identified as monocytes. We compared the map showing only the individuals #63 and #67 (Figure S19) to the ECLIPSE partial model of Fig. 11. The phenotypical marker pattern observed is similar to the cell phenotypes shown in the ECLIPSE partial model. The CD16+ cells might be identified as neutrophils; CD123+ CD193+ CRTH2 cells as basophils; while CD3+CD8+ cells and CD3+ CD4+ cells might be assigned to CD8+ T cells and CD4+ T cells, respectively.

## Discussion

The ECLIPSE methodology allows comprehensive exploration of multi-dimensional MFC data specifically for cells that respond upon disease or other perturbations of cellular systems that may be analyzed by flow cytometry. The vast number of cells in responders that are indistinguishable, ‘normal’, from cells present in healthy individuals hampers such an analysis. The methodology demonstrated removes such cells, accurately taking into account the multiset and multivariate features of MFC data. Rather than making the selection of cell subsets based on the expression of marker pairwise combinations (as for manual gating), ECLIPSE performs an automated gating of response-specific cells based on continuous and multivariate variability in surface marker expression compared to the multivariate variability of healthy cells in a control group. This then results in a comprehensive overview and a quantifiable indication of the immune response in terms of specific expression and/or co-expression of cellular markers. Using ECLIPSE for flow cytometry data analysis will result in more precise and elaborate knowledge about the cells specific for the studied host response, in a fast and un-biased manner.

ECLIPSE will be useful in MFC analyses of cells activated upon response, such as in the LPS study. It will also facilitate the analysis of whole blood in individuals with responses of unknown etiology. The latter is illustrated by the analysis of the asthma patients, revealing heterogeneity in their response to the disease.

In the LPS analysis, we showed how ECLIPSE was able to increase resolution and interpretability of the endotoxin–induced immune response based on the relationships among all the markers, due to removal of ‘normal’ cells. In particular, the general ECLIPSE model shows expression (above the average) of the markers CD62L and CD16 for the LPS-induced immune response associated cells in the responder individuals. The same biplot representation indicated a co-expression of markers CD11c, CD11b, CD69 for the cells that highly express CD16. In this way, the algorithm offered a closer look at such a minority of cells, defining their characteristics in terms of phenotypic markers.

Additionally, visualizing individual responder cell distributions provided the exploration of the personalized response to the LPS and focus on diversity among responders. Small differences in the continuum of surface marker expressions may suggest variability in the time of reaction to the endotoxin among the different individuals.

The asthma study shows how ECLIPSE may be used as a flexible method to characterize heterogeneity of poorly understood diseases among individual patients. In such analysis, a general ECLIPSE model was shown to be insufficient in reproducing the heterogeneous response across all individuals. However, quantitative comparison of cell distributions between individuals in the general ECLIPSE model allows stratification into different groups, which can be further analyzed by partial ECLIPSE models. This allows dedicated interpretation of the cell variability associated to the response within each cluster of individuals, allowing a detailed group- or individual-based interpretation of differences in diseases profiles. The applicability and added value of such profiles in clinical decision-making should be examined in a prospective study. An individual personal response model that is exclusively based on the activated cells, may provide an optimal view of a unique profile in the individual patient.

The advantage of increased resolution in individually expressed cell (sub-)populations however comes with the drawback that personal models cannot be compared between individuals as they lack common ground in the model scores and loadings, such that individual and general ECLIPSE models should always be interpreted simultaneously. Full interpretation of the different immune responses across the entire patient cohort therefore requires systematic exploration firstly in the common ECLIPSE model; the findings from this can then be explored in the clustered patient profiles to allow detailed stratification of patients in future studies.

### ECLIPSE compared to classification by Citrus

The quantitative objectives of ECLIPSE, Citrus and viSNE are distinctive for each method, which makes the results derived from their models inherently slightly different and the comparison between the methods not always as straight-forward. In the case of a binary classification problem, Citrus aims to find the differences between an assumed homogeneous control group and a similarly homogeneous group of diseased samples. ECLIPSE equally assumes a homogenous control group, but allows for heterogeneity in the cell variability between diseased samples; it can even explicitly model this heterogeneity in the partial or individual model. This also implies that ECLIPSE does not provide classification accuracies and other metrics that can be directly compared to those of Citrus. Both methods can, however, be compared by the cell subpopulation marker profiles that are distinctive between phenotypes (Supplement Material III). In the case of the LPS dataset, the phenotypes identified by Citrus were highly comparable to the marker profiles of the cell populations found in the ECLIPSE space (Fig. 4). Citrus additionally identified clusters distinctive for the control group, corresponding to mature neutrophils. In fact, Citrus focuses on finding features that are most predictive for the classification endpoint (e.g. controls vs responders). Therefore, also cell populations that are more abundant or specific for a control group may be found to be best predictors. These cells are not considered of primary interest when studying an immune response by the current implementation of ECLIPSE.

We show here that filtering of the ‘normal’ cells with the ECLIPSE-based filter method, positively affects discrimination between control and responder samples, as indicated by the lower error rate also in Citrus. On top of that, removal of normal cells resulted in a considerably lower number of features necessary for the optimal Citrus classification. However, this also provides a fundamental distinction between Citrus and ECLIPSE: the former aims to find an as low as possible number of features to classify samples, and shows the cell clusters distinctive for the control or response group with the marker expressions in histograms. ECLIPSE provides a comprehensive set of single cells that contributes to the distinction of control and responder samples. Features that are considered redundant by Citrus, because they do not improve the prediction, may be needed to describe patient-specific aspects of the response. The only distinctive phenotype found for the LPS responders by Citrus consisted of pre-mature neutrophils (as shown in Figure S10), while our ECLIPSE analysis, additionally to the presence of pre-mature neutrophils, showed a diversity in mature neutrophils associated to a personalized response across the responders.

These results indicate that Citrus is less-well suited for identifying differences between individuals belonging to the same response group and it will fail in identifying putative personalized cell subsets which are not representative of the whole cohort but only of few samples. This explains why Citrus did not provide satisfying classification accuracies for a heterogeneous disease as asthma, as it failed to classify 25–30% of the samples correctly. Although some specific marker profiles were found for the asthma group, such high cross-validation error rate indicates that the identified clusters are not consistent across the entire patient cohort. Therefore, they do not offer an exact overview of the variability present in the asthmatic patients.

Moreover, the clusters which are either predictive for the asthma group or the control group, were not easy to interpret. This is due to the way the Citrus results are visualized. When multiple cell populations are present within one cluster, the single marker histograms do not give information about the co-expression per cell population.

The limits of Citrus in handling heterogeneous dataset was also shown, when it was applied to the synthetic flow cytometry-like data of cell populations characterized by differences in terms of abundance and expression levels of different markers (included in Supplementary Material V). Here, Citrus could only identify cell populations with different relative abundance in the controls compared to the response group; however, it disregarded the relevant individual response-specific cell populations. ECLIPSE is better suited for exploration of the within-group differences, as it reviews individual patient variability. Additionally, we showed how ECLIPSE was able to detect a small cell population, which was only present in one individual. Moreover, removal of normal cells was necessary to correctly model the variability of all the responder cells, as was shown by the orientation of the loadings in the ECLIPSE model (Figure S38).

### ECLIPSE compared to cell visualization by viSNE

Both viSNE and ECLIPSE methodologies reduce the multi-dimensional flow cytometry data into fewer, commonly two, dimensions. In both cases, this provides a 2D-map that retains only part of the variability in the measured data. However, viSNE provides no information about the variability that remains unexplained by the analysis, while in ECLIPSE, the percentage of unexplained variance is displayed and, if needed, this variance may be described by additional PCs without altering the already fitted part of the model. Due to the different algorithm used for the dimensionality reduction, in principle, no heuristic parameter can be used for the quantitative comparison of the two methods. However, the Differences between the cell variability expressed in the ECLIPSE space and in the viSNE maps may be qualitatively compared and interpreted.

The viSNE analyses performed on the two studied datasets showed a big overlap of the responder cells with the control regions, as expected for both studies. Such overlap, however, can hamper the identification of cell populations specific for the response. Secondly, in viSNE, we cannot affirm whether these normal-like cells are overrepresented compared to the controls and thus interesting to describe the immune response. The Difference between Densities (DbD, step 4), performed by ECLIPSE, takes into account both situations that might occur because of immune response: deviation from normal cell marker variability and higher relative abundance of normal immune cells.

An inherent drawback of viSNE is the need to downsample the original data, due to limit in computational power. Populations that are more abundant will be retained in the subsampled data at the expenses of rarer cells. In such situations, the ECLISPE-based filtering of normal cells (which is a more specialized way of subsampling), focusing on the cells associated to the perturbation of the immune system, is more beneficial. We have shown how a less crowded viSNE map with more intuitive interpretation of responder-specific information can be constructed when combined with a responder-specific filter provided by ECLIPSE. An example of such a viSNE map for the LPS study is compared with the analysis run on the complete dataset (Supplementary Material III). Phenotypical patterns distinctive for the cells found in the challenged individuals are better appreciated. In fact, viSNE performed on the ECLIPSE subsampled data reveals three distinctive populations, which are mostly differentiated by CD16 and CD62L marker expressions. Another advantage of ECLIPSE is that the co-expression among the markers is displayed in one single biplot, while in viSNE the single-cell representation cannot be directly linked to the co-expression of the markers measured.

### Multiset pre-processing

Another advantageous hallmark of the ECLIPSE algorithm, next to the removal of normal cells, is the distinctive multiset pre-processing strategy. As described in the Supplementary Material I, the novel pre-processing strategy adopted by the algorithm is able to account for differences in terms of number of cells measured per individual, such that each individual equally contributes to the model. Unwanted variation present between the individuals is also taken care of by the pre-processing steps, which helps to systematically remove variability unrelated to the problem while retaining relevant biological information. We have also shown in Figure S14 (Supplementary Material III) how the elimination of individual variability done by ECLIPSE was beneficial for analysis with the viSNE algorithm, which might have been affected by sample-to-sample variability. Such multiset pre-processing was also beneficial to outperform the predictive ability of the discriminant model obtained with Citrus (Figure S7).

### Multivariate advantage

The results obtained with the two different studies, show how the multivariate advantage may be used to unravel the correlations among all the measured surface markers and to distinguish cell subsets from one another. Other methods like SPADE and viSNE lack this view of mutual correlation among surface markers; any information on surface marker co-expression can only be obtained from a *post hoc* model interpretation, analyzing multiple single marker heatmap plots. The SCA loadings of ECLIPSE provide a direct compass-like representation of the surface marker co-expression on specific subsets of cells that may be much easier interpreted in terms of surface marker co-expression than, for example, viSNE maps. A direct interpretation of marker co-expression, compared to the qualitative evaluation necessary for non-linear models such as viSNE, justifies the use of linear SCA. Non-linear relationships are, anyway, still visible in the ECLIPSE model. In fact, the LPS-specific variability appears in the ECLIPSE space as a ‘bean-like’ continuum (Fig. 2) across co-expression of both CD16 and CD62L.

Although FLOOD offers an explicit view of the correlations among surface markers through a similar biplot representation, this method lacks the filter aspect (removal of ‘normal’ cells) of ECLIPSE. This makes the FLOOD loadings considerably less informative than those from ECLIPSE, because the former also describe ‘normal’ cells to a large extent, which dilutes their response-specific information. This demonstrates the direct benefit of ECLIPSE for the comprehensive and specific interpretation of the immune response across a range of responders/patients. In addition, the use of Difference between Densities instead of the convex hull used in FLOOD, for determining the boundaries of the filter to gate out ‘normal’ cell brings several advantages. While the control data cannot be described using only a single convex shape, the Difference between Densities gating step can easily discern different populations. In addition, the DbD is able to take into account not only deviation from the normal cell surface variability but also changes in the fraction of normal cells, that might increase due to the immune response.

## Conclusion

ECLIPSE provides a filter for ‘normal’ cells present in individuals out of homeostasis, based on the multivariate co-expression among all the cellular markers measured. As a result, the algorithm allows the identification of detailed cellular information particularly present in response individuals (e.g. patients) completely independent of prior expertise knowledge, but through automatic and data-driven cell gating. ECLIPSE is specifically valuable for the identification of unbiased cellular information present in flow cytometry data of individuals with a condition of unknown etiology. The ECLIPSE method provides a higher resolution on response-specific cells that might be hidden among an overwhelming preponderance of normal cells. In future application of the ECLIPSE algorithm, we may expect that ECLIPSE could benefit researchers and clinicians handling intricate data, such as data containing rare cell subsets that only occur in diseased samples.

In addition, the versatility of the algorithm may allow the integration of the multidimensional ECLIPSE filter into other multivariate MFC data analysis methods, in order to enable a better visualization and an outperforming prediction accuracy.

### Software and Data availability

ECLIPSE is implemented in MATLAB®R2016b and the script is available for download at the https://github.com/RoelBouman/ECLIPSE.

The data sets used in the ECLIPSE analyses are available at https://www.ru.nl/science/analyticalchemistry/research/data-analytical-chemistry/

## Electronic supplementary material

Supplementary information
