# Supplementary material for: Automated flow cytometric identification of disease-specific cells by the ECLIPSE algorithm
Source: Sci Rep. 2018 Jul 19;8:10907. doi: 10.1038/s41598-018-29367-w (PMC6053450; doi:10.1038/s41598-018-29367-w)
Supplement: Supplementary file 1 — Supplementary information [file 41598_2018_29367_MOESM1_ESM.pdf]

Automated flow cytometric identification of disease specific cells by the ECLIPSE algorithm

Rita Folcarelli[a*], Selma van Staveren[b,c], Roel Bouman[a] , Bart Hilvering[c], Gerjen H. Tinnevelt[a,b], Geert Postma[a], Oscar F. van den Brink[b], Lutgarde M. C. Buydens[a], Nienke Vrisekoop[c], Leo Koenderman[c], Jeroen J. Jansen[a]

[a]*Radboud University, Institute for Molecules and Materials, Analytical Chemistry, P.O. Box 9010, 6500 GL Nijmegen, The Netherlands*

[b]*TI-COAST, Science Park 904, 1098 XH Amsterdam, The Netherlands*

[c]*Department of Respiratory Medicine and laboratory of translational immunology, University Medical Center Utrecht, Heidelberglaan 100, 3584CX, Utrecht, The Netherlands*

Email: r.folcarelli@science.ru.nl

Contents

Supplementary Material I ... 3
Multi-set control/response structure ... 3
Step0: Data pre-processing ... 5
Supplementary Material II ... 9
Additional results of ECLIPSE on the LPS dataset ... 9
Additional results of ECLIPSE on the asthma dataset ... 14
Supplementary Material III ... 17
Citrus and viSNE analyses on the LPS and asthma study dataset ... 17
Results of Citrus on LPS data ... 17
Results of viSNE on LPS data ... 21
Results of Citrus on the asthma data ... 27
viSNE analysis of asthma data ... 30
Supplementary Material IV ... 33
Manual sequential gating of the asthma red cluster and projection into ECLIPSE space ... 33
References ... 38

Supplementary Material I

Multi-set control/response structure and pre-processing of Multicolour Flow Cytometry Data

Multi-set control/response structure

MFC data quantify the presence of fluorescently labelled cell properties, such as surface marker expressions, of a large number of single cells. These cells are collected per individual sample and the number of collected cells may vary considerably between cell 'sets'. Experiments often compare a 'Case' (or responder) group to samples obtained from 'Control' individuals. The Control individuals present surface marker expression on their cells typical for individuals that do not display the response of interest, while the individuals in the 'responder' group present cell populations with surface marker expressions characteristic of the immune response studied.

Figure S1 shows the possible arrangements of the MFC data by considering three different levels. (1) Single matrices, which hold the cell set measured per individual; comprehensive and comparative analysis of different samples require that the same surface markers are measured across all individuals. (2) Single matrices are concatenated column-wise leading to $\mathrm{X} = \begin{bmatrix} \mathrm{X}_1 \\ \vdots \\ \mathrm{X}_I \end{bmatrix}$ of size $\sum_1^I N_i \mathrm{x} J$, where N_i is the number of cells of the ith individual and J corresponds to the markers measured, $1 \ldots j \ldots J$. This resulting matrix $\mathbf{X}$ therefore consists of a 'multiset', where each set contains the cells of one individual; each row within each set represents measurement of one single cell. Each individual might be either a control or a responder, the information of the respective group is displayed in the level (3) of the multiset structure with the matrix $\mathbf{X}_{i_g}$ size $N_{i_g} \mathrm{x} J$.

The index $g = 0$ for the control and $g \geq 1$ for the responder groups that might correspond to different diseases or subtypes of the same disease. If all responder individuals are drawn from a population with the same disease, then g will assume values 0 and 1, for the control group

and responder group, respectively. In some cases, experiments are paired, which means that the same person is followed and analysed before and after an immune response.

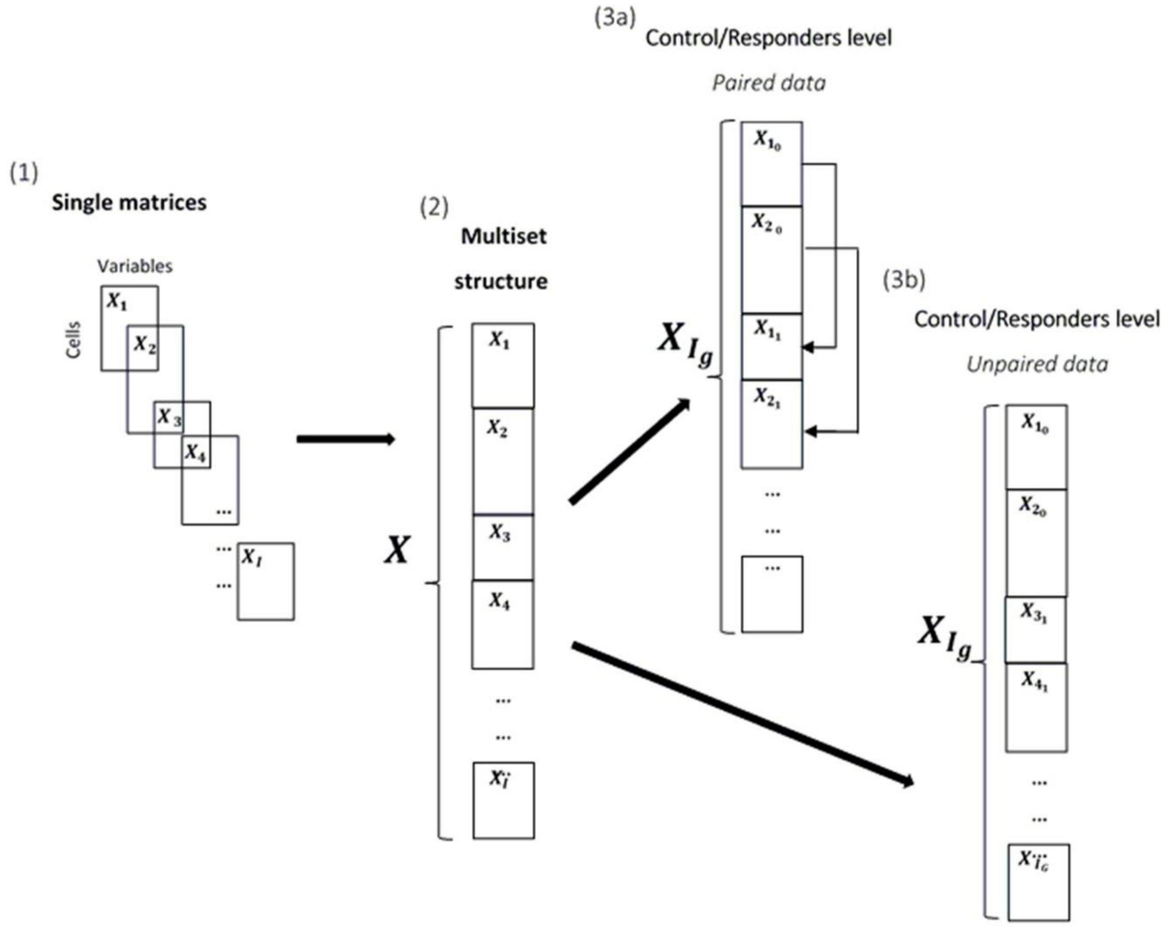

Figure S1: (1) Single data matrices representing measurement per individual, (2) Multiset data arrangement obtained by linking the matrices column-wise, (3a) Control/Responder differentiation of the multiset structure, with paired data, (3b) Control/Responder differentiation of the multiset structure, with paired data

In this case the index i, representing the individuals, is not unique (3a). However, the two Flow Cytometry case studies we consider in this paper both consist of unpaired individuals, indicated by different i (as in 3b).

Step 0: Data pre-processing

Data pre-processing is a very important aspect of chemometric data analysis.[1] It aims to remove variability in the data that is unrelated to the problem under study, while retaining the experimentally relevant information. In Flow Cytometry, such irrelevant variability might derive from instrumental artefacts due to misalignment of the laser source, baseline drift, laser power variability, interference of fluorescent labels, or uninformative noise coming from low intensity signals. Removing such irrelevant variability in a quantitative and reproducible way facilitates the quest for relevant biomedical information and guarantees that the fluorescents intensities are measures of level of protein expression on the cells.

The first step of the pre-processing consists of transforming matrix $\mathbf{X}$ using log (or arcsinh) function. In MFC, this type of transformation is essential to cope with the broad dynamic range of emissions between fluorophores. The data matrix may contain negative values, due to background subtraction or to the compensation of overlap between emission spectra of different fluorophores[2]. These require a more dedicated transformation, such as the arcsinh scaling described by Finak et al.[3]. Multivariate analysis of the cell variability then requires centering and scaling of the log or otherwise-transformed matrix $\mathbf{X}_{\log} = \log_{10}(\mathbf{X})$ Eq. *S1*. Mean (or median) centering subtracts the column mean (or median) from every element in the column, which removes surface marker expression consistently present across all cells, resulting in the variability in surface marker expressions across the cells. Scaling equalizes the variability of each surface marker across the cells, to allow them to contribute equally to a multivariate model of the data, regardless of the intensity of the used fluorophore or the absolute variability in abundance of every surface marker.

Both centering and scaling need to take into account the multi-set structure of the MFC data (Fig. 1). Several strategies for centering and scaling may accommodate this structure and the quantitative comparison between case and responder individuals[4].

One strategy is centering and scaling the variables per set, i.e. per individual.

(a) $\mathbf{m}_{i_g} = \frac{1}{N_{i_g}} \mathbf{1}_{N_{i_{i_g}}}^{\mathbf{T}} \mathbf{X}_{\log,i_g}$ Eq. S2

(b) $\mathbf{X}_{\mathbf{m}_i} = \mathbf{X}_{\log,i_g} - \mathbf{1}_{N_{i_g}} \mathrm{m}_{i_g}^{\mathrm{T}}$

Where $\mathbf{m}_{i_g}$ is the mean calculated for the surface markers measurements of the i_g–th individual, $i_g = 1_g, \ldots, I_G$, hold in the matrix $\mathbf{X}_{\log,i_g}$ of size N_{i_g}xJ; N_{i_g} corresponds to the number of cells of the i_g–th individual; $\mathbf{1}$ is a column vector of ones with length N_{i_g}. The resulting mean centered matrix $\mathbf{X}_{\mathrm{m},i_g}$ is thus scaled using as weight the inverse of $\mathbf{S}_{i_g}$, diagonal matrix of size JxJ holding the standard deviation of the mean-centered surface markers of the i_g–th individual, calculated as follows:

(a) $\mathbf{s}_{i_g}^{\mathbf{T}} = \sqrt{var(\mathbf{X}_{\mathbf{m}_i})}$

(b) $\mathbf{S}_{i_g} = \mathbf{diag}\left(\mathbf{s}_{i_g}^{\mathbf{T}}\right)$ Eq. S3

(c) $\mathbf{X}_{\mathrm{sc}} = \mathbf{X}_{\mathrm{m},i_g}\,\mathbf{S}_{i_g}^{-\mathbf{1}}$

Centering (Eq. S2b) and scaling (Eq. S3c) *per* individual may correct technical individual-specific offsets due to e.g. changes and/or misalignment of laser intensity, sample handling etc. that do not contribute to the biomedical information within the MFC dataset[5].

Next to pre-processing per individual, there are other pre-processing options, based on the class-level (control or response) or the whole dataset. Considering a single class, specifically the 'control group' ($g = 0$), as a reference for 'normal' cell variability may highlight those cells that are specific to a response in the other class(es). Centering and scaling based on the control individuals selectively emphasizes deviations from the 'normal' cell variability observed in the case individuals, when means and the standard deviations used in Equations. S2 and S3 are calculated across the control individuals. This requires correcting for the considerably different numbers of cells per analysed individual, in order to avoid the individual with most cells dominating the calculated means and standard deviations. This correction is accomplished by pooling the means of each set into a weighted class mean, according to Eq. S4.

(a) $\mathbf{m}_0 = \frac{\sum_{1_0}^{I_0} \mathbf{m}_{i_0}}{I_0}$

(b) $\mathbf{X}_{\mathrm{m}_0} = \mathbf{X}_{\log} - \mathbf{1}_{N_{i_g}}\mathrm{m}_0^{\mathrm{T}}$ Eq. S4

where $\mathbf{m}_{i_0}$is the mean of the log-transformed surface marker intensities of the i_0–th control individual, calculated according to Eq. S2a, with $g = 0$; I_0 is the total number of control individuals. $\mathbf{X}_{\mathrm{m}_0}$, of size of size N_{i_g}xJ, represents the multiset matrix centered using the class mean of the log-transformed surface marker intensities of the control class.

Also the cumulative control standard deviation may be calculated by Eq. S5:

$$\text{(a)}\ \ \mathbf{s}_0^{\mathbf{T}} = \sqrt{\frac{\sum_{1_0}^{I_0} var(\mathbf{X}_{\mathrm{m},i_0})}{I_0}}$$

$$\text{(b)}\ \ \mathbf{X}_{\mathrm{sc}_0} = \mathbf{S}_0^{-\mathbf{1}}(\mathbf{X}_{\mathrm{m}_0}) \qquad \text{Eq. S5}$$

with $\mathbf{X}_{\mathrm{m},i_0}$ the mean-centered individual control matrix, estimated according Eq. S2b, with $g = 0$; $\mathbf{S}_0^{-\mathbf{1}}$ the diagonal matrix holding the standard deviation $\mathbf{s}_0^{\mathbf{T}}$ weighted for the number of control individuals I_0; $\mathbf{X}_{\mathrm{sc}_0}$ the multiset matrix resulting from the auto-scaling performed with the weighted mean and standard deviation across the control individuals. From $\mathbf{X}_{\mathrm{sc}_0}$ we can extrapolate the pre-processed matrix of the control and responder sets, expressed in Eq. S6 and Eq. S7, respectively:

$$\mathbf{X}_{\mathrm{sc}_0} = \begin{bmatrix} \boldsymbol{X}_{\mathrm{sc}_{\mathbf{1_0}}} \\ \vdots \\ \boldsymbol{X}_{\mathrm{sc}_{\boldsymbol{I_0}}} \end{bmatrix} \qquad \text{Eq. S6}$$

$$\mathbf{X}_{\mathrm{sc}_1} = \begin{bmatrix} \boldsymbol{X}_{\mathrm{sc}_{\mathbf{1_1}}} \\ \vdots \\ \boldsymbol{X}_{\mathrm{sc}_{\boldsymbol{I_1}}} \end{bmatrix} \qquad \text{Eq. S7}$$

Among the different options, centering and scaling based on the control class (Eqs. S4b, S5b), will enhance the deviation of the responder individuals from the cell variability observed in the control individuals. Alternatively, individual centering and scaling (Eqs. S2b, S3b) might be a preferable option when there are considerable shifts in the observed surface marker expressions of cells between different samples. These operations now rid the resulting data of uninformative offsets and allow each surface marker to *a priori* contribute equally to the model fitted subsequently, taking into account both the multiset and control/responder structure of the data. However it should be noted that the last option has a disadvantage. When all cells of one response individual show up or downregulation of one or multiple markers compared to the cells of the control individuals, this information will be lost due to individual centering.

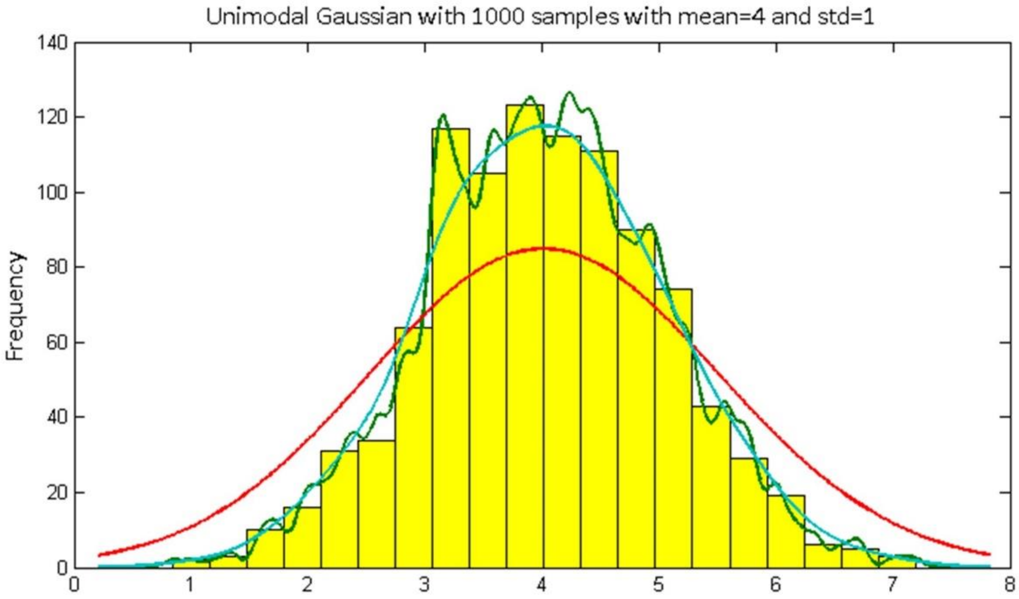

Figure S2: The effect of Kernel bandwidth (h in Equation 4, main article) on the estimate of a (normal) distribution. In yellow: a histogram of 1000 samples from a Unimodal Gaussian distribution with mean 4 and standard deviation 1. In green: an under smoothed estimate using a small bandwidth. In red: an over smoothed estimate using a large bandwidth. In light-blue an estimate using a near optimal bandwidth.

Supplementary Material II

Additional results of ECLIPSE on the LPS dataset

As an example of the heterogeneity between responders, the ECLIPSE results obtained from Individuals #14 and #16 are shown in Figure S3. Two groups of neutrophils, mainly separated along the first component, can be observed in both individuals. However, in Individual #14 the two populations are not as well distinguished from each other as in Individual #16. Cells with a continuum in surface marker expressions connects the two populations at the upper part. In addition, Individual #14 presents a quite heterogeneous distribution within the mature neutrophils that can be encircled by two different gates. The percentages of the cells included in the gates for Individual #14 and #16 are reported in Table S1. Individual #16 displays two already well distinguishable homogenous populations. This indicates a difference in response between the two individuals, which for instance could suggest that the immune system of Individual #16 responded faster to the LPS stimulus when compared to the other individual.

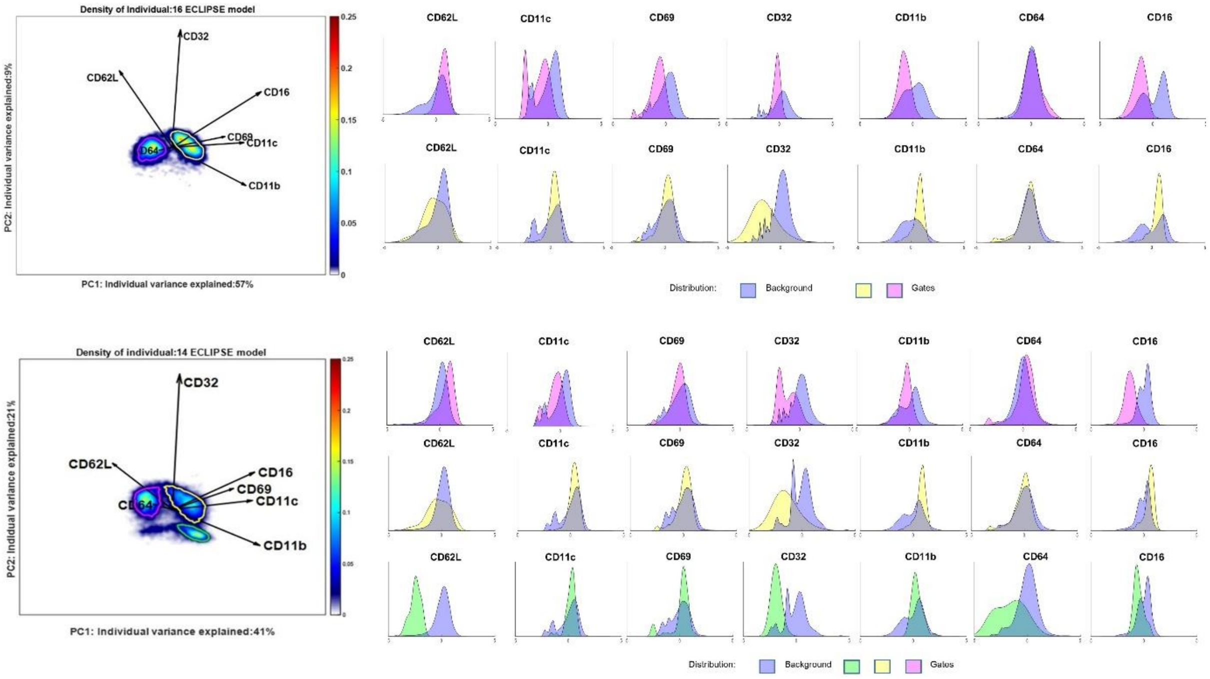

Figure S3: Visualization of two responder individuals in the ECLIPSE model, built after eliminating the normal cells. The variance explained by the general model for each individual is displayed on the axis. The individuals present different distribution of the responding cells that can be selected by different gates, based on the higher density estimation. The histograms show the marker expression of the cells within the different gate; the distribution are displayed with the colour of the corresponding gate. Percentage of the cells, normalized on the original total amount of cells, is visualized in Table 1.

Table S1: Percentages of the total cells of responder individuals #14 and #16 in the gates displayed in Fig. S3.

	Gate magenta	Gate yellow	Gate green
ID #14	13%	18%	7%
ID #16	16%	27%	0-

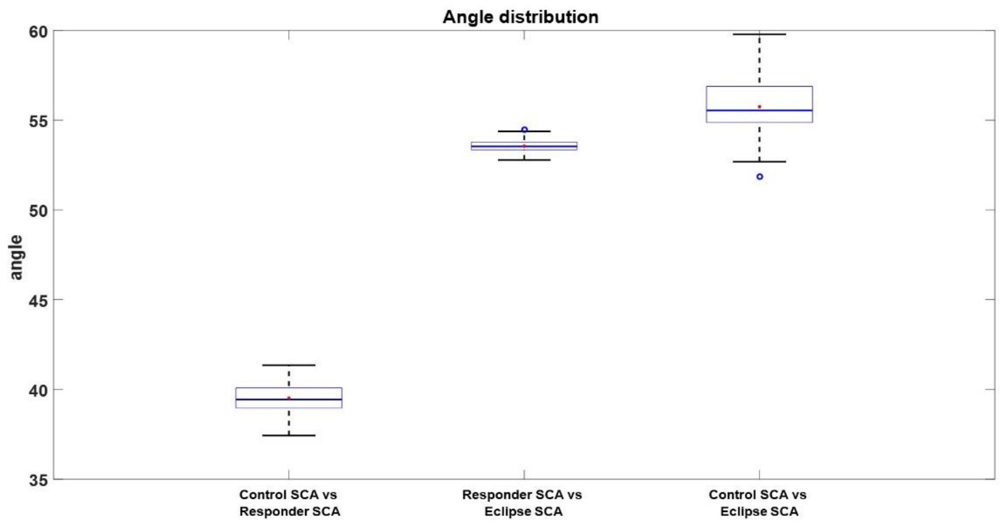

Figure S4: Box plot of the angle distributions between the sub-spaces defined by loadings of different SCA models. The first comparison shows that the Control SCA and Responder SCA models are the most similar as shown by the smaller angle than those obtained by the comparison of each of the models to the ECLISPE SCA model.

Table S2: ECLIPSE plots of the LPS response group per individual.

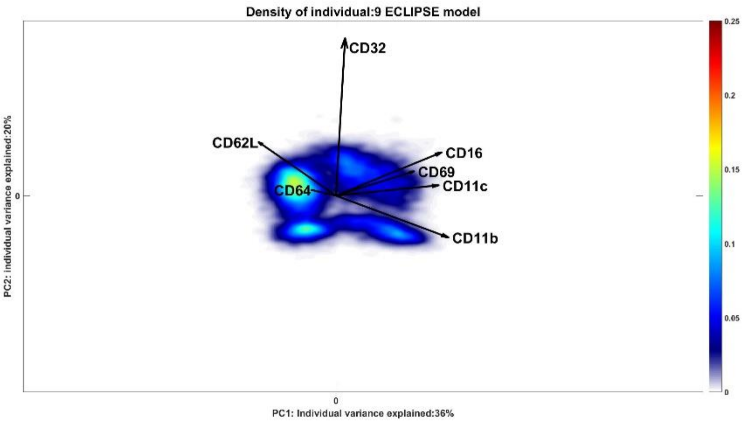

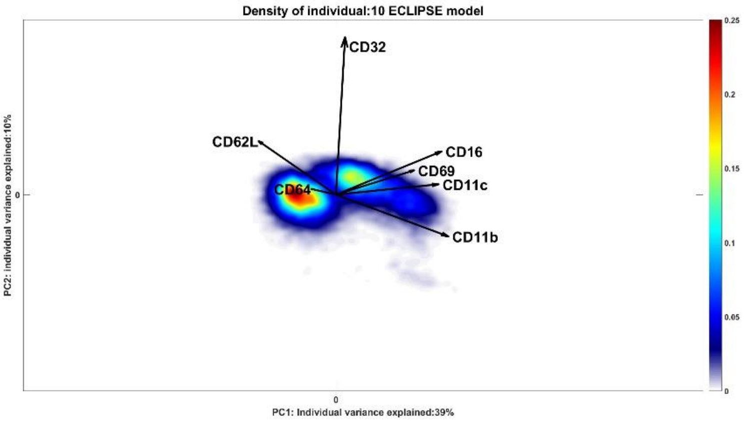

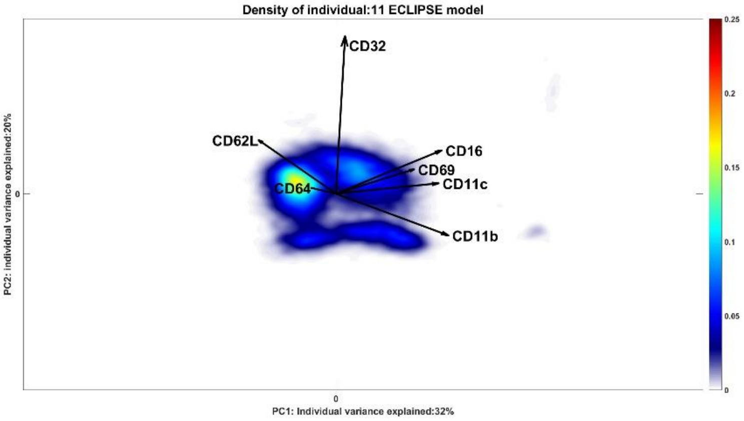

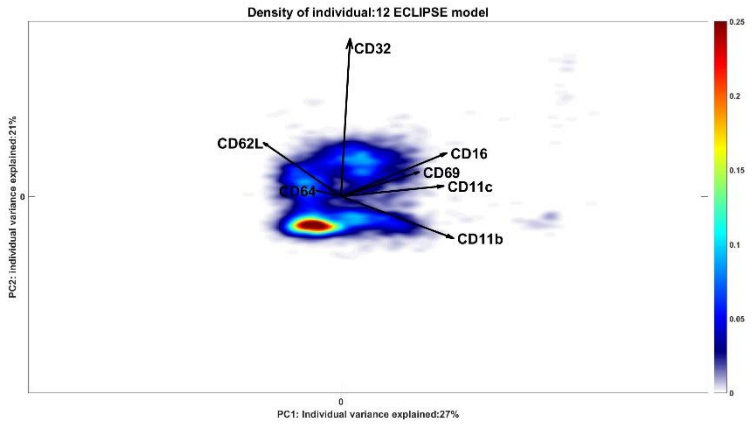
Density of individual:12 ECLIPSE model
CD32
CD62L
CD16
CD69
CD64
CD11c
CD11b
PC2: individual variance explained:21%
PC1: Individual variance explained:27%
0
0
0.25
0.2
0.15
0.1
0.05
0

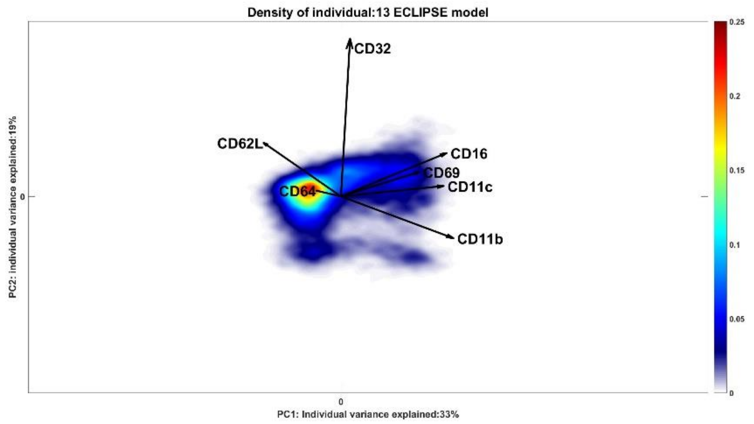
Density of individual:13 ECLIPSE model
CD32
CD62L
CD16
CD69
CD64
CD11c
CD11b
PC2: individual variance explained:19%
PC1: Individual variance explained:33%
0
0
0.25
0.2
0.15
0.1
0.05
0

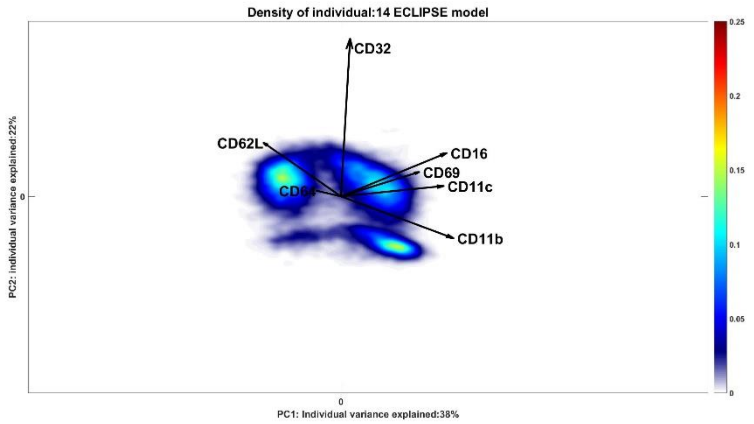
Density of individual:14 ECLIPSE model
CD32
CD62L
CD16
CD69
CD11c
CD11b
PC2: individual variance explained:22%
PC1: Individual variance explained:38%
0
0
0.25
0.2
0.15
0.1
0.05
0

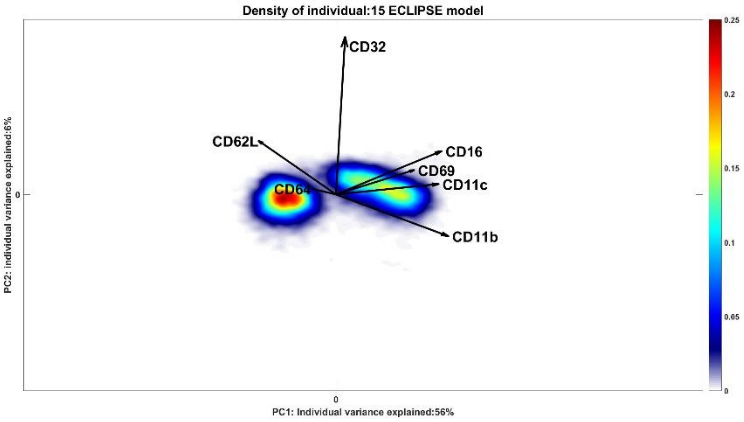

Density of individual:15 ECLIPSE model
CD32
CD62L
CD16
CD69
CD64
CD11c
CD11b
PC2: individual variance explained:6%
PC1: Individual variance explained:56%
0.25
0.2
0.15
0.1
0.05
0

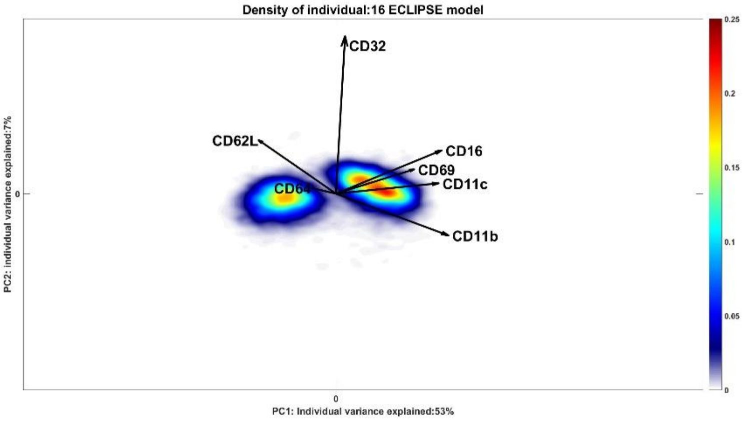

Density of individual:16 ECLIPSE model
CD32
CD62L
CD16
CD69
CD64
CD11c
CD11b
PC2: individual variance explained:7%
PC1: Individual variance explained:53%
0.25
0.2
0.15
0.1
0.05
0

Additional results of ECLIPSE on the asthma dataset

Table S3: Density plots of all the asthma patients in the ECLIPSE model Space

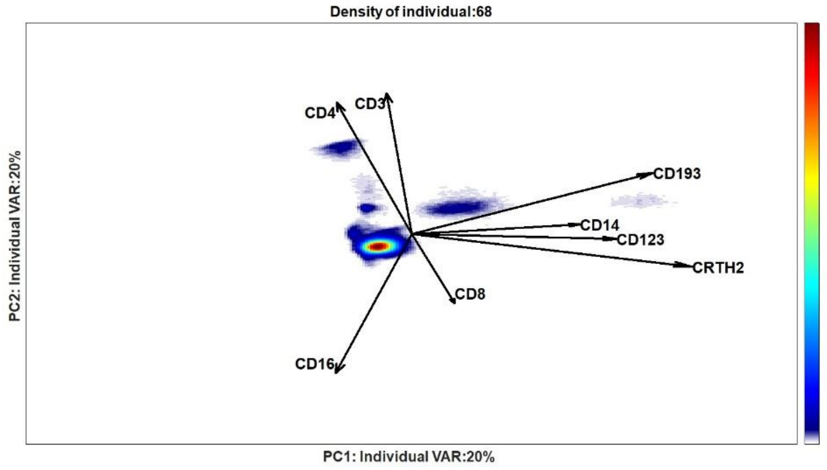

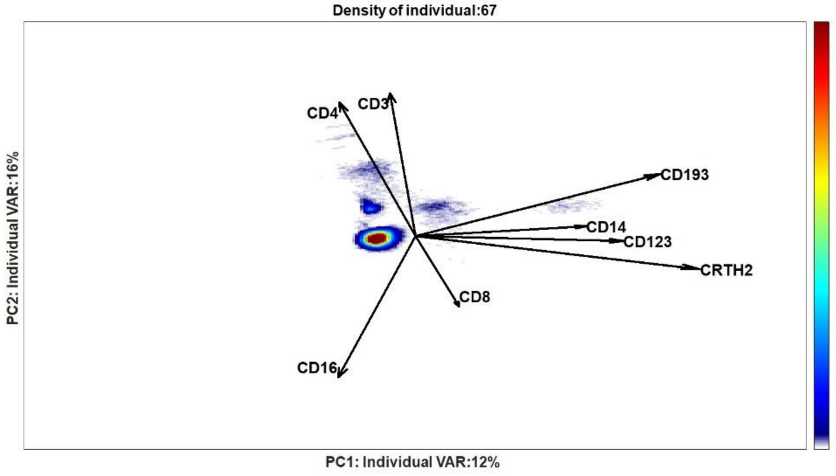

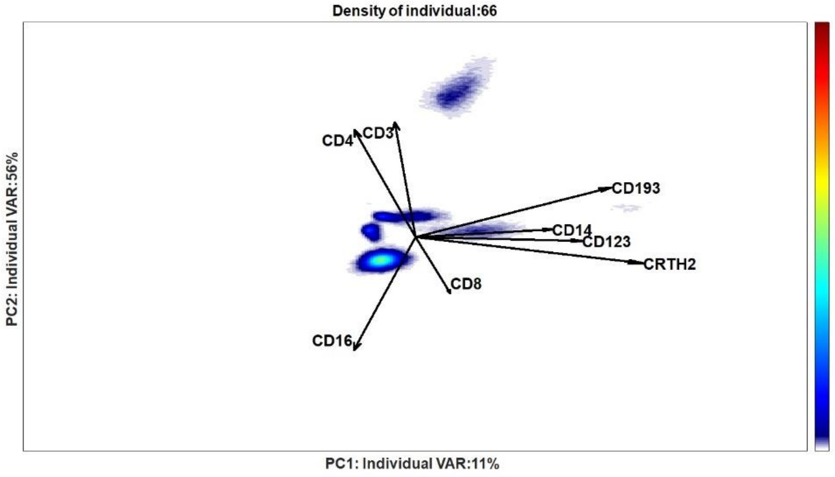

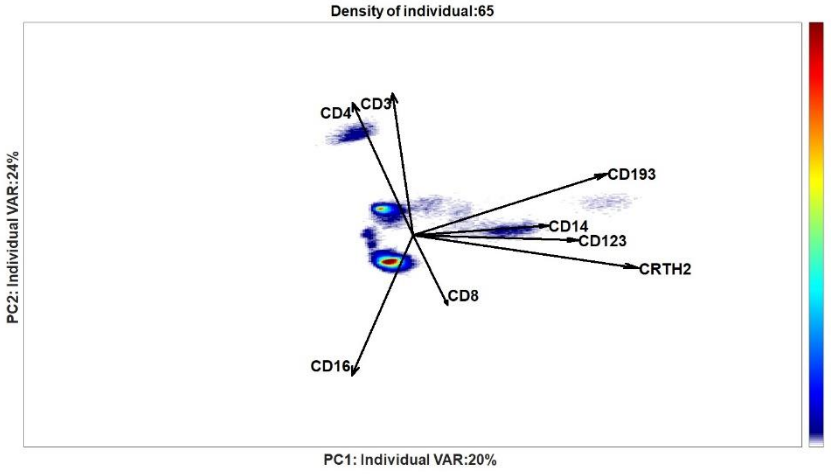
Density of individual:65
PC2: Individual VAR:24%
PC1: Individual VAR:20%
CD4
CD3
CD193
CD14
CD123
CRTH2
CD8
CD16

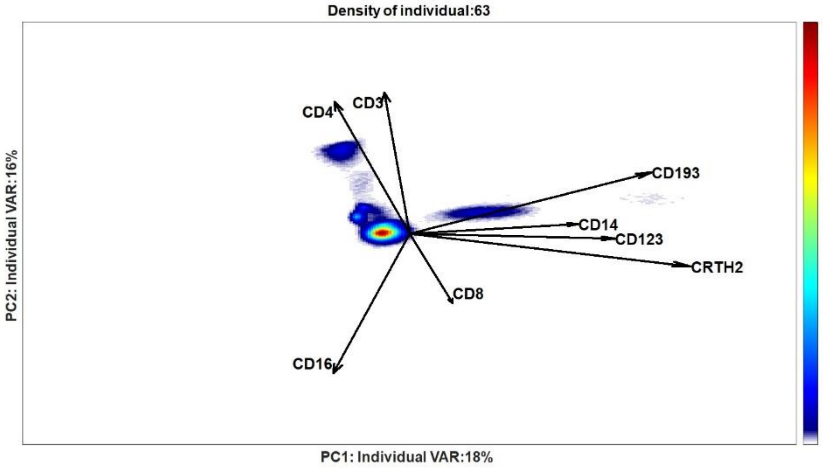
Density of individual:63
PC2: Individual VAR:16%
PC1: Individual VAR:18%
CD4
CD3
CD193
CD14
CD123
CRTH2
CD8
CD16

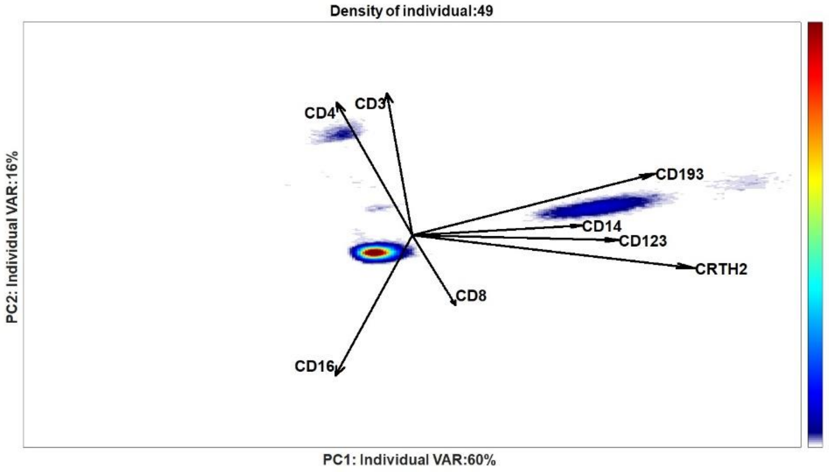
Density of individual:49
PC2: Individual VAR:16%
PC1: Individual VAR:60%
CD4
CD3
CD193
CD14
CD123
CRTH2
CD8
CD16

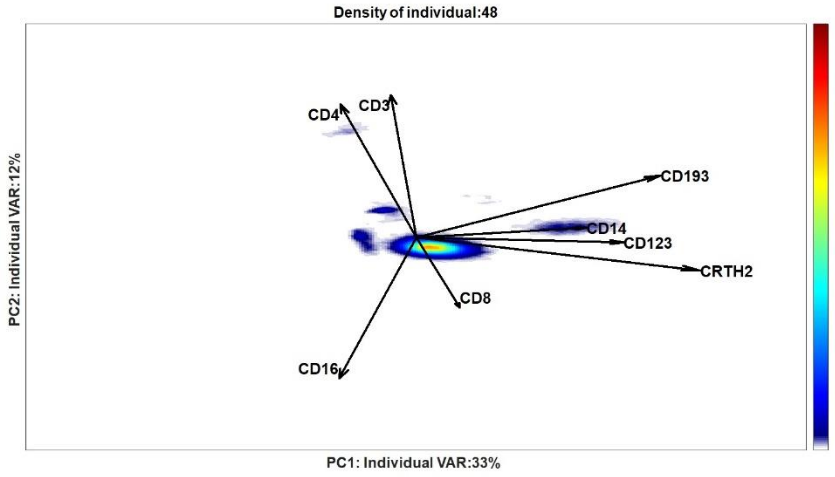
Density of individual:48
CD4
CD3
CD193
CD14
CD123
CRTH2
CD8
CD16
PC2: Individual VAR:12%
PC1: Individual VAR:33%

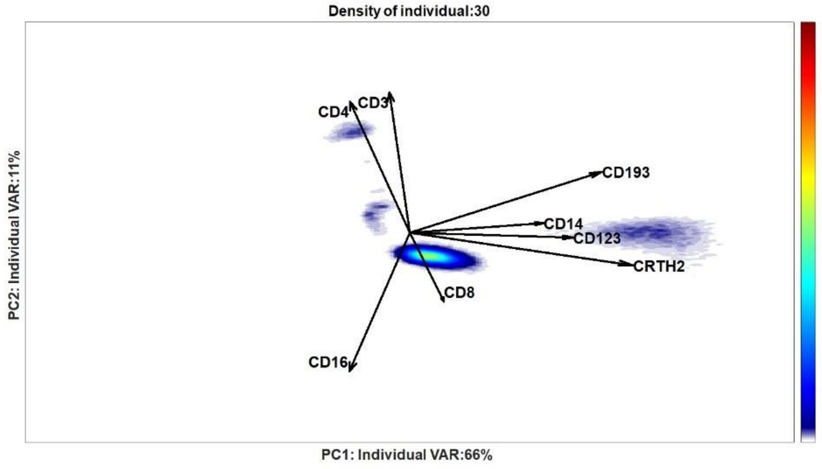
Density of individual:30
CD4
CD3
CD193
CD14
CD123
CRTH2
CD8
CD16
PC2: Individual VAR:11%
PC1: Individual VAR:66%

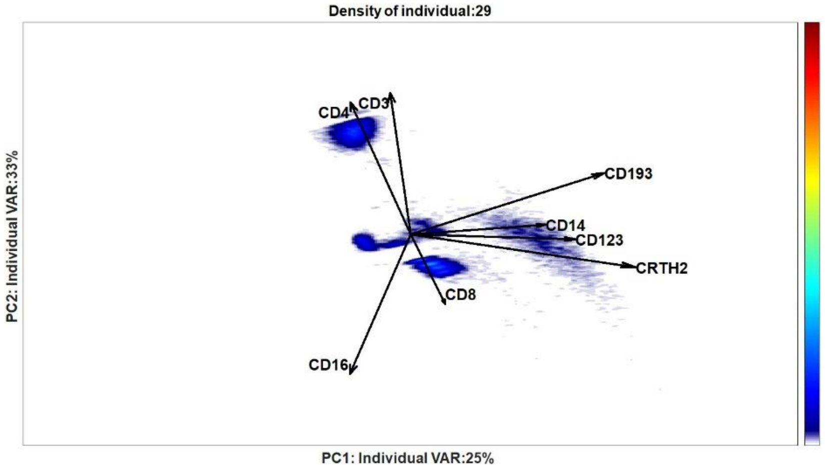
Density of individual:29
CD4
CD3
CD193
CD14
CD123
CRTH2
CD8
CD16
PC2: Individual VAR:33%
PC1: Individual VAR:25%

Supplementary Material III

Citrus and viSNE analyses on the LPS and asthma study dataset

Results of Citrus on LPS data

Citrus[6] uses hierarchical clustering to identify phenotypically similar cell populations. Descriptive cell cluster-specific features are calculated on per-individual basis. These features can include, for example, the proportion of cells in the identified cluster for each individual. For intergroup analysis, the method employs a regularized classification model to detect the group-specific cell clusters for each sample.

By definition, a regularized model selects subsets of features of the data to achieve the best prediction, avoiding overfitting. The constant *regularization threshold* regulates the number of features used for the classification. A series of models with increasing complexity, i.e. with increased number of selected features, is built by varying this threshold. The fit of each model is estimated through cross-validation. A plot of the Model cross-validation Error Rate versus Regularization Threshold enables investigators to assess the quality of the results of the classification and the fit of the model chosen by Citrus analysis. An optimal model has low cross-validation error rate, which corresponds to a low percentage of misclassified samples. When the error remains constant within a range of increasing number of features, the regularized threshold associated to the fewest number of features is chosen in order to select the most informative features that differ between the two groups.

Citrus was applied to the LPS dataset using the R GUI. The regression classification model was trained on the data using the default pre-processing of the GUI, which implies arcsinh transform with cofactor 5. The accuracy of the classification models constructed is shown in Figure *S5*.

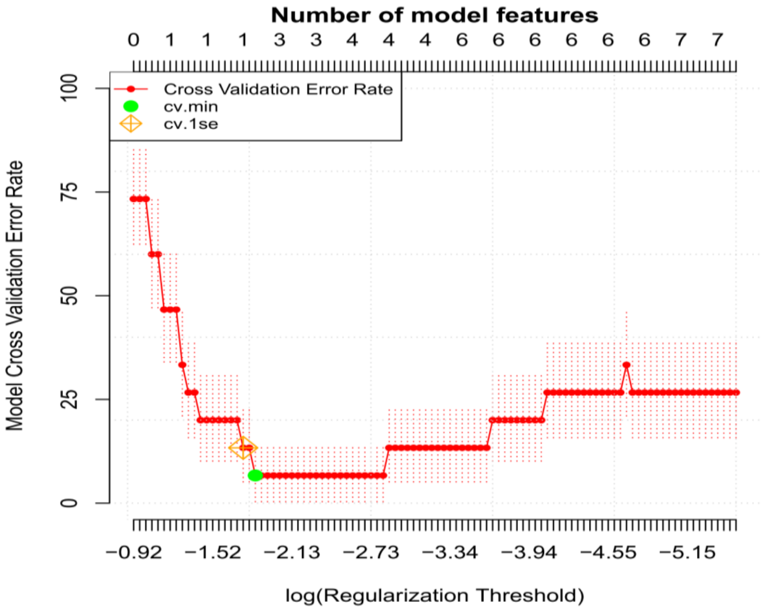

Figure S5: The figure shows the Model Cross-validation Error Rate vs the log(Regularization Threshold) for the classification models constructed on the arcsinh-transformed LPS dataset. The number of the features, used to build the model, associated to the different regularized thresholds is shown on the top of the plot. The green circle (cv.min) points out the model with the smallest number of features necessary to obtain the lowest cross-validation error; while the orange diamond (cv_1se) indicates the model with the smallest number of features associated to cross-validation error 1 std higher than the minimum error. The model with cv.min is chosen by the Citrus analysis and this corresponds to an error rate of around 7%.

The model, identified as optimal by the cross-validation procedure (cv.min in Figure *S5*), incorrectly classifies around 7% of the samples. Two cell clusters were selected as the most discriminating ones between LPS responders and controls by the cross-validated model. The histograms of these clusters, both more abundant in the LPS responder, are shown against the background cluster, which contains all the rest of the cells not included in the specified cluster (Figure *S6*).

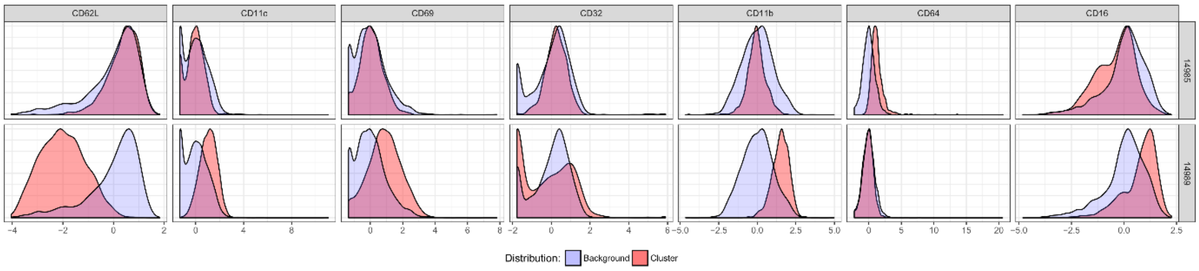

Figure S6: The histograms show the phenotype of the cells belonging to the cluster (red) selected by the cross-validated model. The background histograms (blue) show the rest of the data, not included in the cluster. Cells from cluster 14985 are characterized by the distinctive phenotype CD16-and+CD62L+CD64+; cells from cluster 14989 are CD16+CD11b+CD69+CD11c+CD62L-. Both clusters are more abundant in the responder group.

Cluster 14989 has a distinctive phenotype, existing of CD16+CD11b+CD69+CD11c+CD62L− cells. Two CD32 populations are present in this cluster: CD32− and CD32+. In contrast, cells from cluster 14985 express high levels of CD64 and CD62L, while they have a dim expression of CD16. The cells present in the two cluster might be identified as activated mature and partly immature (CD16^{dim}) neutrophils, respectively. Using the ECLISPE method we also identified two LPS-specific cell clusters in the ECLIPSE space generated when plotting PC1 and PC2, Figure 4. To be able to compare these results, we applied Citrus on the pre-processed data as used in the ECLIPSE analysis. Subsequently, we applied Citrus on the LPS response-specific cells after ECLIPSE cell elimination.

First, Citrus was applied on the data pre-processed with the ECLISPE algorithm, as described in *Pre-processing section*. In this analysis, all the cells were included. The cross-validated error rates of the obtained models are displayed in Figure *S7*.

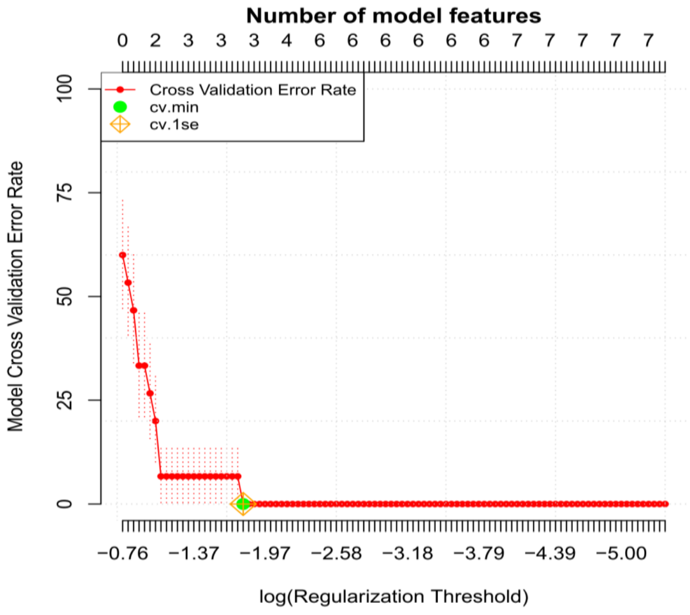

Figure S7: Cross-validation error rate plot for LPS dataset transformed with the ECLIPSE pre-processing procedure: arcsinh transformation, individual mean center and control scaling. Model Cross Validation Error Rate equals to 0 is obtained with 3 stratifying features.

A perfect classification is achieved with a model which uses three distinctive features. This model is chosen as optimal by the Citrus Analysis. The most discriminant three clusters identified by the model are shown in Figure *S8*. Cluster 14956 and 14993 represent phenotypes that might be assigned to mature neutrophils, with CD16+CD62L+ and CD16+CD62L+CD32−, respectively. These clusters are found more abundant in the control group, compared to the

responder group for which cluster 14990 is more present. This last cluster, with CD16−CD69− and CD62L+ can be assigned as immature neutrophils.

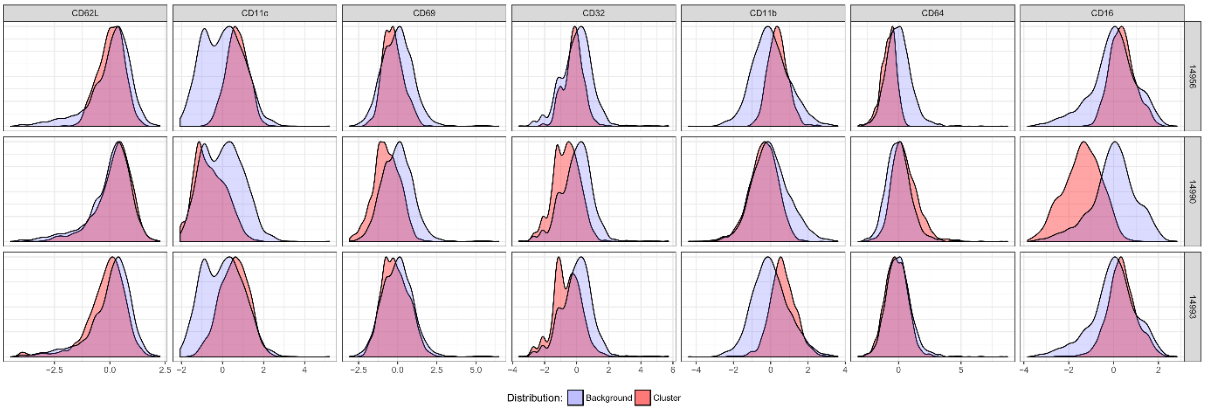

Figure S8: Histograms show the phenotypes of the three cluster identified by the cross-validated classification model. Cluster 14956 and 14993 present phenotypes that might be assigned to mature neutrophils, with CD16+CD62L+and−. The cluster 14990, more abundant in the responder group, is identified as premature neutrophils with CD16−CD69− and CD62L+.

Finally, we applied Citrus to the LPS dataset, after the elimination of normal cells, provided by ECLIPSE. The model chosen by the Citrus analysis offers a perfect classification of the individuals in the two groups, as shown in the cross-validation error rate plot (Figure *S9*). In this case, however, the discriminating model requires a lower number of informative features. In fact, only one feature is necessary to correctly classify the samples. This feature corresponds to the abundance of cluster 14994 in the responder group. The phenotype of this cluster (Figure *S10*) indicates that the most discriminant population is represented by cells CD16−CD11b−CD62L+CD69−CD11c−/dim, which might correspond to gate **a** in Figure 4 of ECLIPSE, defined as immature neutrophils. In contrast to the previous analyses, there are no clusters of mature neutrophils identified as most discriminant ones between the groups.

The results obtained by the three analysis show how individual mean-centering and control scaling has a positive effect in the predictive ability of the Citrus algorithm. The ECLIPSE algorithm, at the current stage, is not developed to be used as a classification model. It is more suited for explorative research, finding cell populations that arise upon an immune response. These results can give further insight in the mechanism behind the immune response studied. Due to the different purposes of the models, a direct comparison of the prediction accuracy of the two methods is not possible. However, the last analysis, performed on the data after removal of normal cells, brings a further improvement of the classification model in terms of

complexity. This shows the power of the subsampling provided by ECLIPSE, since in principle the ECLIPSE subsampling may be beneficial for discriminant classification methods as Citrus.

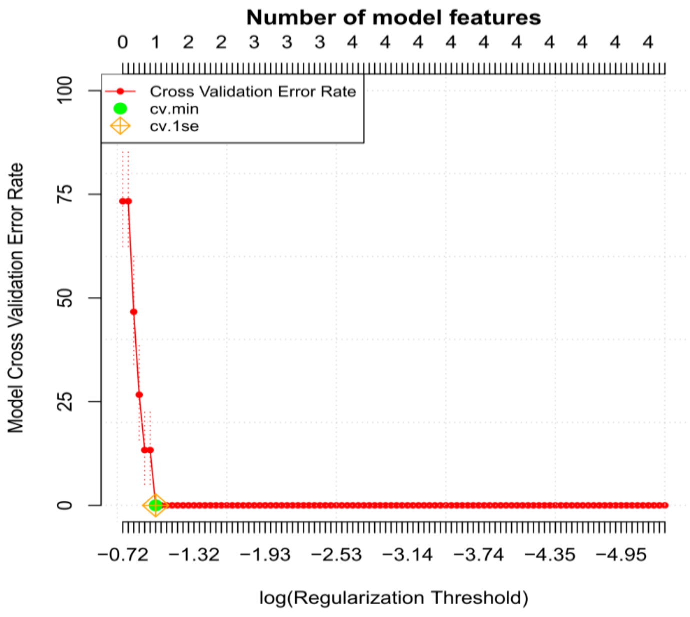

Figure S9: Cross-validation error rate vs Regularization threshold plot for LPS dataset transformed with the ECLIPSE pre-processing procedure (arcsinh transformation, individual mean center and control scaling), after elimination of normal cells. Model Cross Validation Error Rate null is obtained with 1 stratifying feature.

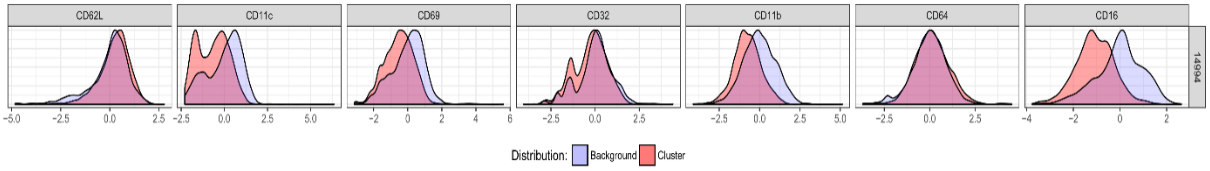

Figure S10: Histograms of the phenotypes of the most discriminant cluster, more abundant in the responder group. Cells in the cluster are CD16−CD11b−CD62L+CD69−CD11c−/dim.

Results of viSNE on LPS data

viSNE[7] analysis was applied to the LPS dataset using the Matlab GUI cyt (downloadable from the website https://www.c2b2.columbia.edu/danapeerlab/html/cyt.html). In order to obtain a better visualization of single-cell resolution, we run all the analyses on a subset of the LPS dataset. Each individual was subsampled to 2000 cells, so that the total amount of cells was 30,000. The analysis was first performed using the default transformation present in the GUI, consisting of arcsinh transformation with cofactor 5. The results are shown in Figure S*11*. The

upper left panel (Figure *S11*a) show the cells in the viSNE map coloured per individual. It seems that cells of the same individual are grouped together, suggesting that the cluster found by the algorithm are individual specific. The viSNE map in the upper right panel (Figure *S11*b) shows cells coloured per control/response group: a considerable overlap of cells between control and response individuals is observed. Distinctive region for the responders seems to be the upper left region and the right area. However, not all the response individuals show cells in those regions.

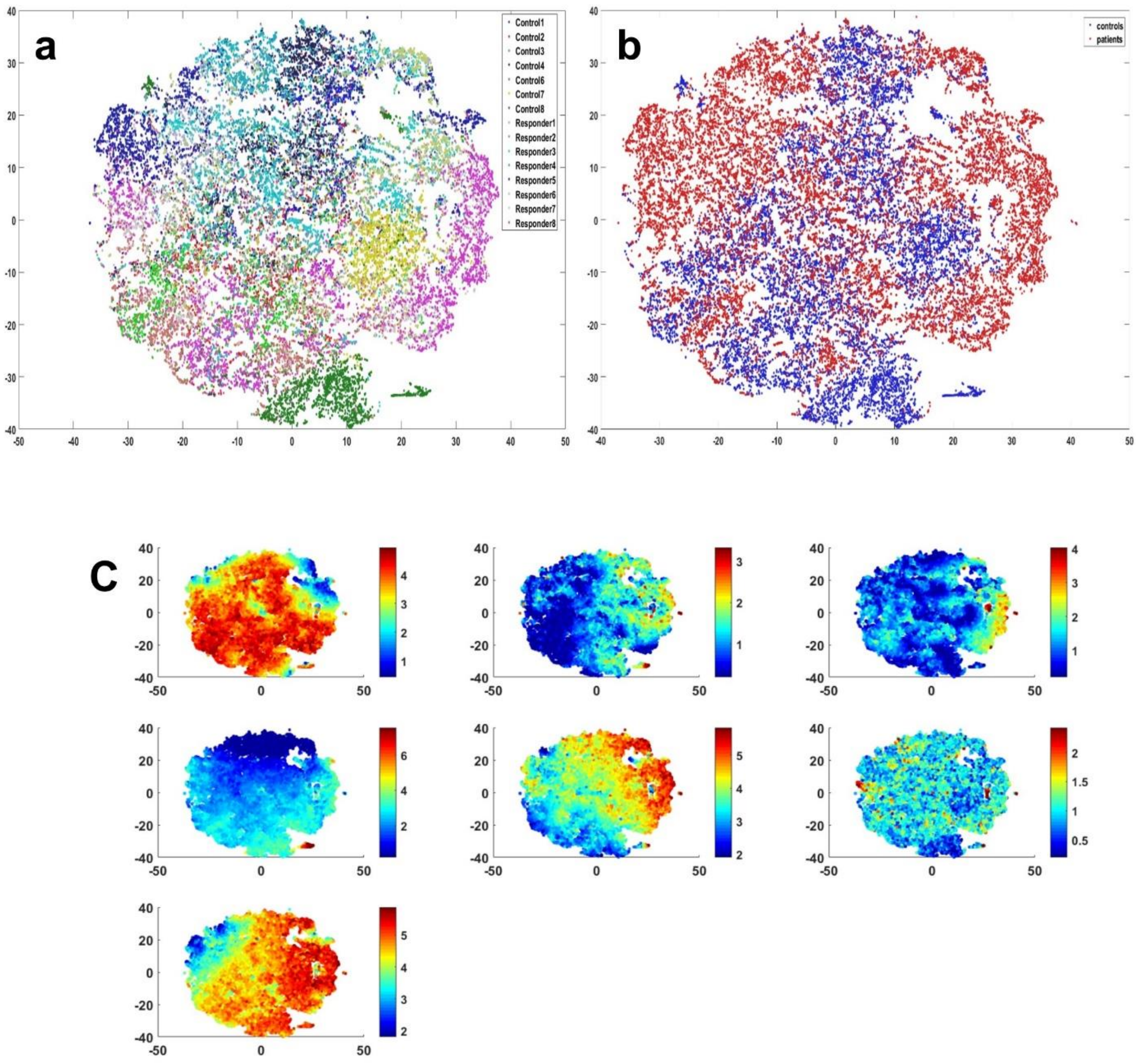

Figure S11: viSNE analysis performed on the LPS data pre-processed with the default arcsinh transformation present in the cyt gui. 11a: cells in the viSNE map are coloured per different individual; 11b: cells from the responder individuals, in the viSNE map, are coloured based on control (blue) and responder (red) group. 11c: cells are coloured based on expression of the single 7 markers.

Figure *S12* shows the constructed viSNE map only for the responder individual *ID14*, for whom we show the ECLIPSE model in the Supplementary material II (Figure *S3*, upper panel). LPS-specific cells CD62L−CD16+CD11b+ (upper right) and CD62L+andCD16− (upper left) can be observed. These cell populations were clearly distinct also in the ECLIPSE space.

A big overlap is present with the control region. However, in viSNE, we cannot affirm whether these normal-like cells are overrepresented compared to the controls and thus interesting to describe the immune response. The Difference between Densities (DbD, step 4), performed by ECLIPSE, takes into account both situation that might occur because of immune response: deviation from normal cell marker variability and overproduction of normal immune cells.

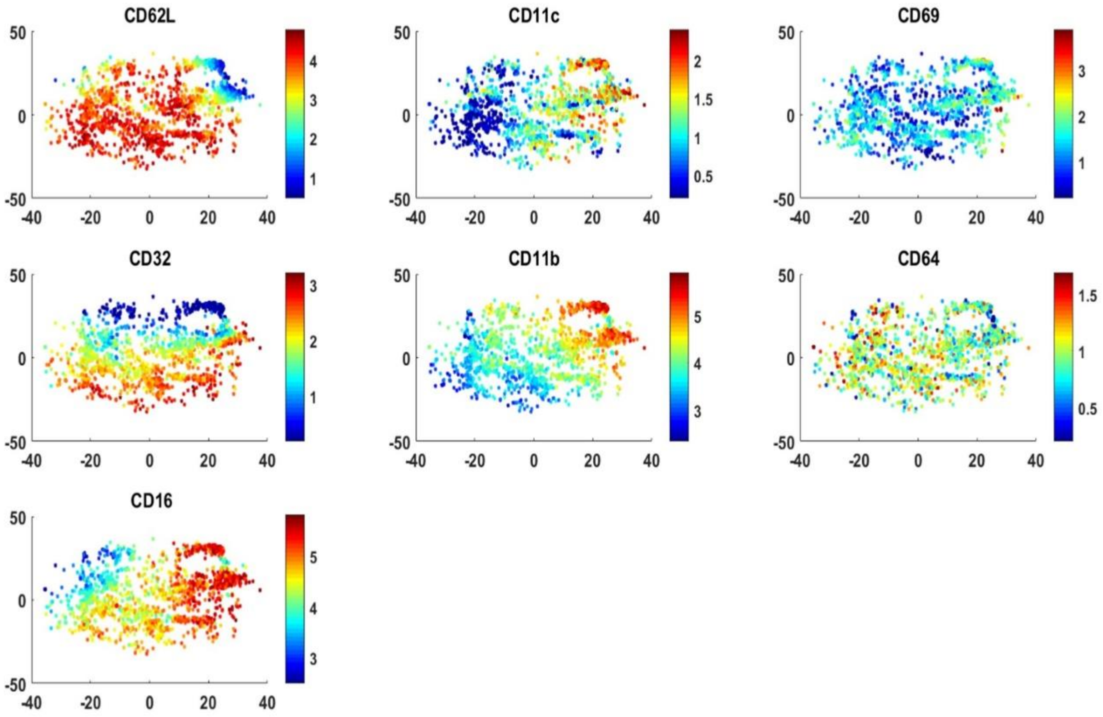

Figure S12: Cells from the responder individual with ID14 shown on the viSNE map built on all the control and responder individuals. The panels show the marker expression of the measured marker.

Secondly, we performed viSNE on the LPS data pre-processed by the ECLIPSE algorithm; the results can be observed in Figure *S13*. The viSNE map, coloured per individual, shows how the cells are distributed across the map and no donor-specific clusters are present. In fact, the multiset pre-processing specifically adopted for the LPS study by ECLIPSE (as described in the

Pre-processing section of Supplementary Material I, Equations *S2b* and *S5b*), helps to remove the individual variability of the control individuals. In this case, the individual control variability is biologically not that relevant and introduces noise into the results. It might originate from instrumental variability, for instance.

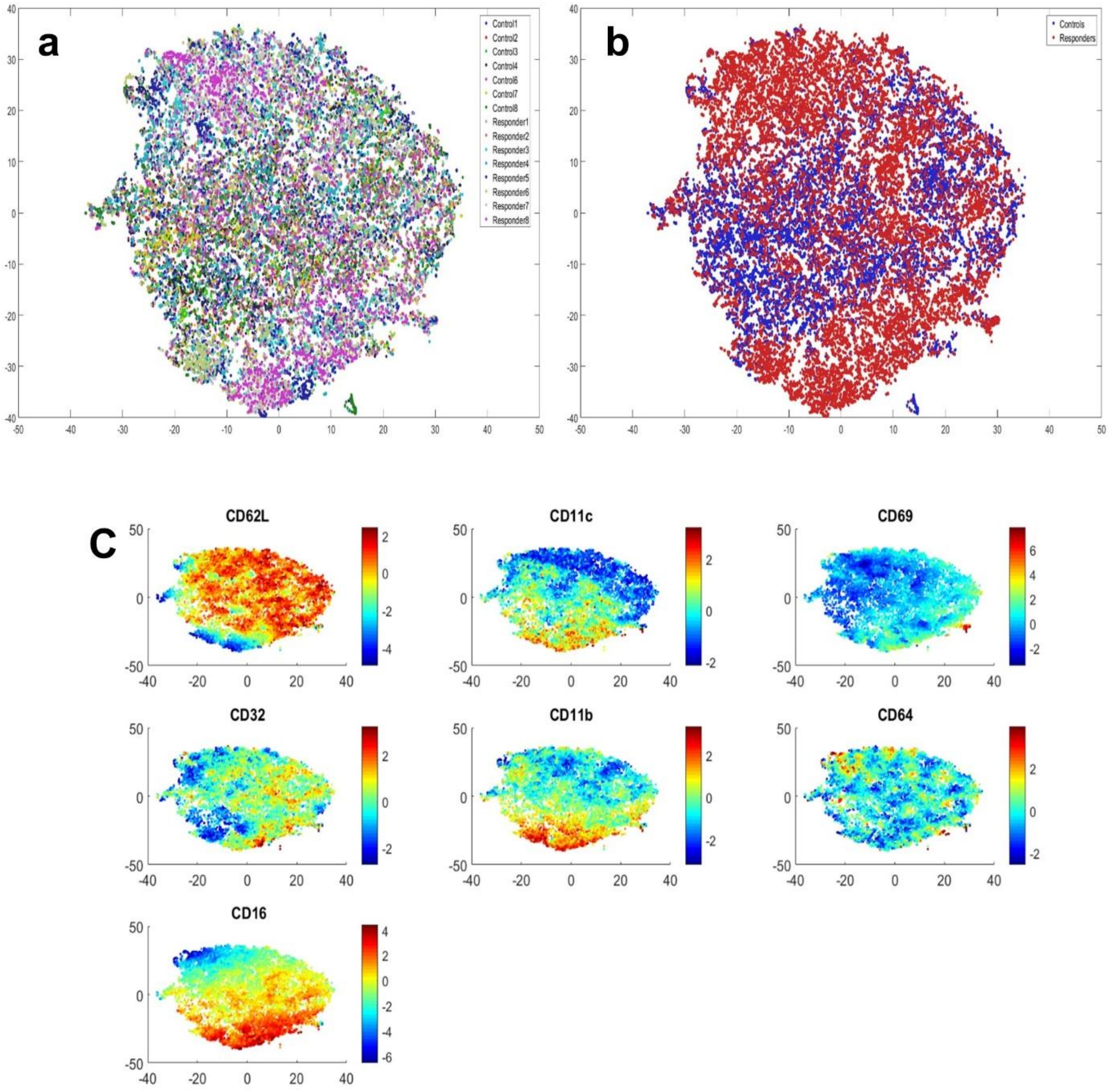

Figure S13: viSNE analysis performed on the LPS data mean-centered and scaled according to the ECLIPSE algorithm. 13a: cells in the viSNE map are coloured per different individual; 13b: cells in the viSNE map are coloured based on control (blue) and responder (red) group. 13c: cells from the responder individuals, in the viSNE map, are coloured based on expression of the single 7 markers.

Finally we applied viSNE to the LPS data pre-processed by ECLIPSE, but after removal of normal cells from the responder individuals, performed by the ECLIPSE algorithm. The results are

shown in Figure *S14*. It is clear how the cells from control individuals (blue, Figure *S14b*) are placed in the middle of the viSNE map, whereas the cells from the responder individuals (red, Figure *S14b*) are distributed in the upper and lower region of the map. Very little overlap is present between cells of the two groups and clearly, a lower amount of responding cells is present. The marker expression of these remaining cells is shown in Figure *S14*c. ECLIPSE removes mostly the CD16dimCD62L+ and CD16−CD62L− cells, leaving two quite distinctive populations. No particular difference in marker expression is noted in the viSNE analysis, performed on the ECLISPE subsampled data.

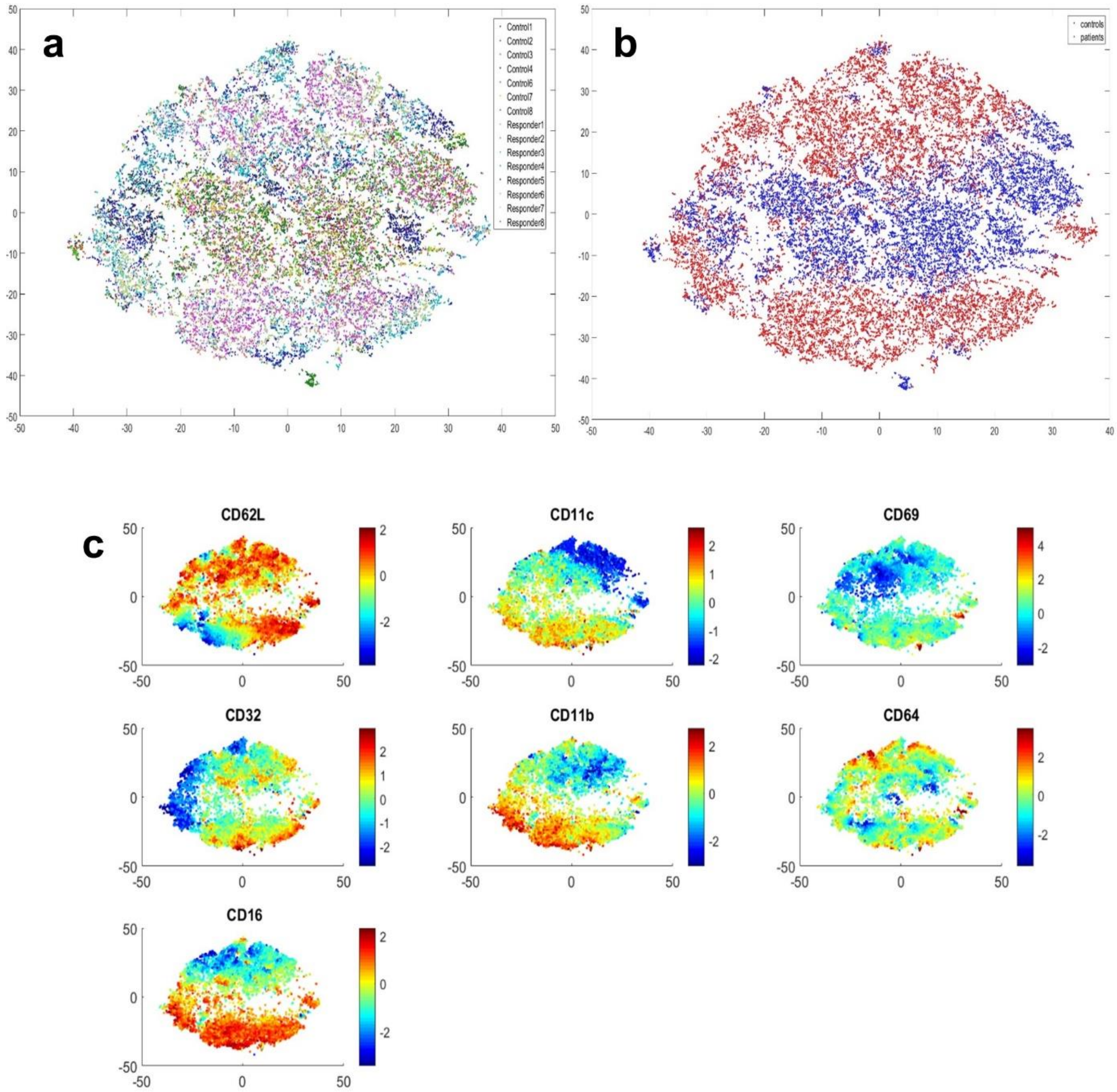

Figure S14: viSNE analysis performed on the LPS data mean-centered and scaled, after removal of normal cells in the responder done by ECLIPSE. 14a: cells in the viSNE map are coloured per different individual; 14b: cells in the viSNE map are coloured based on control (blue) and responder (red) group. 14c: cells from the responder individuals, in the viSNE map, are coloured based on expression of the single 7 markers.

The viSNE algorithm is used mostly for data dimensionality reduction and visualization purposes, while the main goal of ECLIPSE is the selection of interesting cellular information and/or populations based on multiple marker co-expression. In principle, no quantitative parameter can be used for the comparison with the ECLIPSE results on the LPS data. However, we have shown how the viSNE analysis is susceptible to sample-to-sample variation, which has influence on the resulting map. The multiset pre-processing operated by ECLIPSE systematically reduces this variation.

Both ECLIPSE and viSNE performed on the ECLIPSE subsampled data reveal two distinctive populations, which are mostly differentiated by CD16 and CD62L marker expressions. However, an advantage of ECLIPSE is that the co-expression among the markers are displayed in one single biplot. Secondly, ECLIPSE defines response-specific cell populations, also taking into account normal cells that are overrepresented upon an immune response. These cells are not specifically defined when analyzing data with the viSNE algorithm. Next to this, the hallmark of ECLIPSE is the removal of normal cells, leading to a less crowed representation, which enables to distinguish the LPS responding cell populations better, also in the viSNE map (Figure *S14*).

Results of Citrus on the asthma data

We trained Citrus on the asthma data using the R GUI. The model error rates plot of the built models is shown in Figure S*15*.

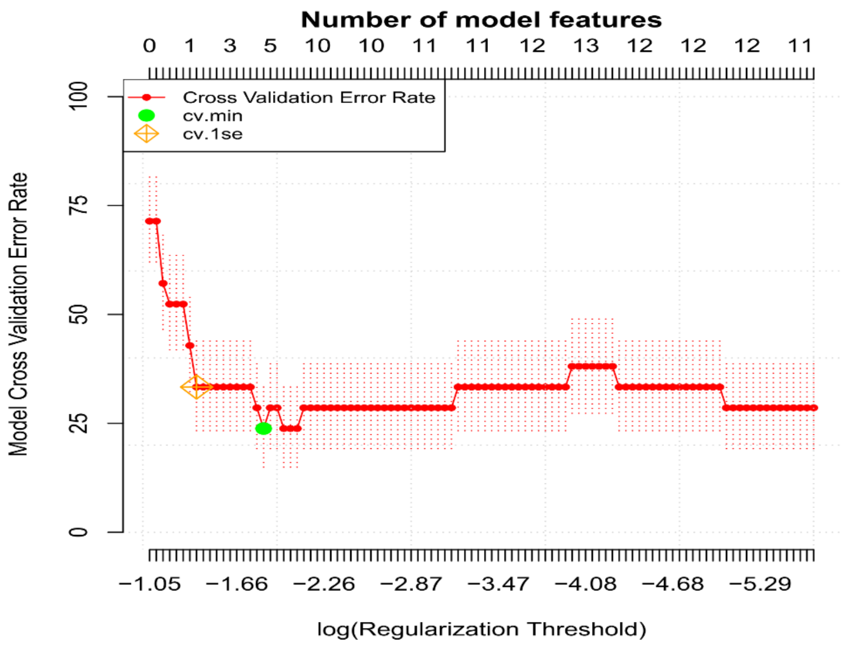

Figure S15: The figure shows the Model Cross-validation Error Rate vs *the log(Regularization Threshold) for the classification models constructed on asthma data. The green circle (cv.min) points out the model with the smallest number of features necessary to obtain the lowest cross-validation error, which corresponds to 25% of misclassified samples; the orange diamond (cv_1se) indicates the model with the smallest number of features associated to cross-validation error 1 std higher than the minimum error, which leads to an error of around 30%.*

The highest accuracy achievable by the analysis correspond to 25% of misclassified samples. Four features were necessary for this minimal error (Figure S*15*, cv.min). A model with a cross-validation error 1 std higher than the minimum, corresponding to 30% of misclassification, requires 1 feature (Figure S*15,* cv.1se). The clusters detected by both models are shown below.

The feature identified by the model cv.1se corresponds to the abundance of cluster 41977, found to be more abundant in the control group (group 1) compared to the asthmatic individual (group 2) (Figure S*16*). The phenotype indicated that the cluster is mostly characterized by CD4+ T cells, having CD3+CD4+ and CD8−CD16−CD123−CD14−CRTH2− expression levels.

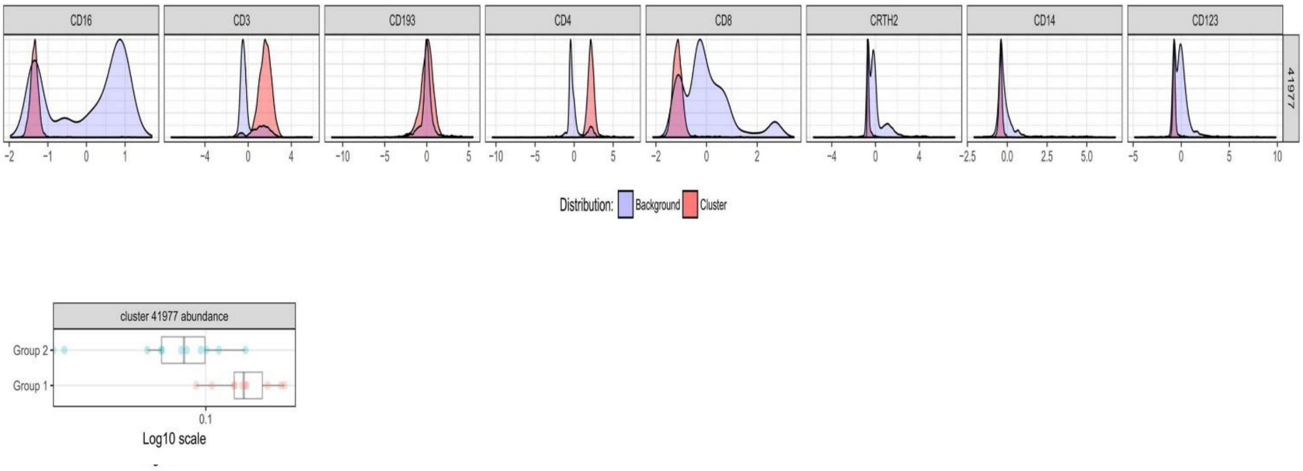

Figure S16: The upper panel shows the phenotype of the most discriminant cluster between control and asthmatic individuals, found by the model associated with cv.1se error (~30%). This cluster is on average more present in control group (group 1) than in asthmatic (group 2), as displayed on the lower panel. Cells in the specified cluster can be recognized as CD4+CD3+ and CD8- T cells.

The cell clusters found by the best performing model are shown in Figure S*17*. Cluster 41962 is characterized by cells with CD16+CD123dimCD8dim and CD3−CD14− expression levels; while cluster 41977 has a CD4+ T cell phenotype (CD3+CD4+). These clusters are more abundant in the control group compared to the asthmatic. This is in contrast to the ECLIPSE results, in which we found CD3+CD4+ cells present in the asthmatic patients after the ECLIPSE elimination step of normal cells.

The last two clusters are more represented in the asthmatic group when compared to the control group. For these clusters, it is harder to identify the cell populations. Cluster 41979 consists of CD16+CD14dimCRTH2+CD123+ cells, which might be identified as monocyte derived cells. As shown by the cluster abundance graph on the right, there is high variability in the occurrence of this cellular cluster among the asthmatic patients. The last cluster 41994 has a very heterogeneous expression pattern, with multiple peaks per marker. |This indicates the presence of multiple cell types within the cluster. Based on the smaller peaks with high CD193, CRTH2 and CD123 expression, basophils and/or eosinophils might be identified within this cluster. In addition, CD3+CD8+ T cells might be present in this cluster. However, co-expression

of these markers should be verified to be able to draw these conclusions. When multiple cell types are present within one Citrus cluster, it is impossible to state which cell types are represented, since the single marker histograms do not give information about co-expression of markers. ECLIPSE, on the other hand, allows interpretation of co-expression from the location of the cell populations and the respective orientation of the loadings.

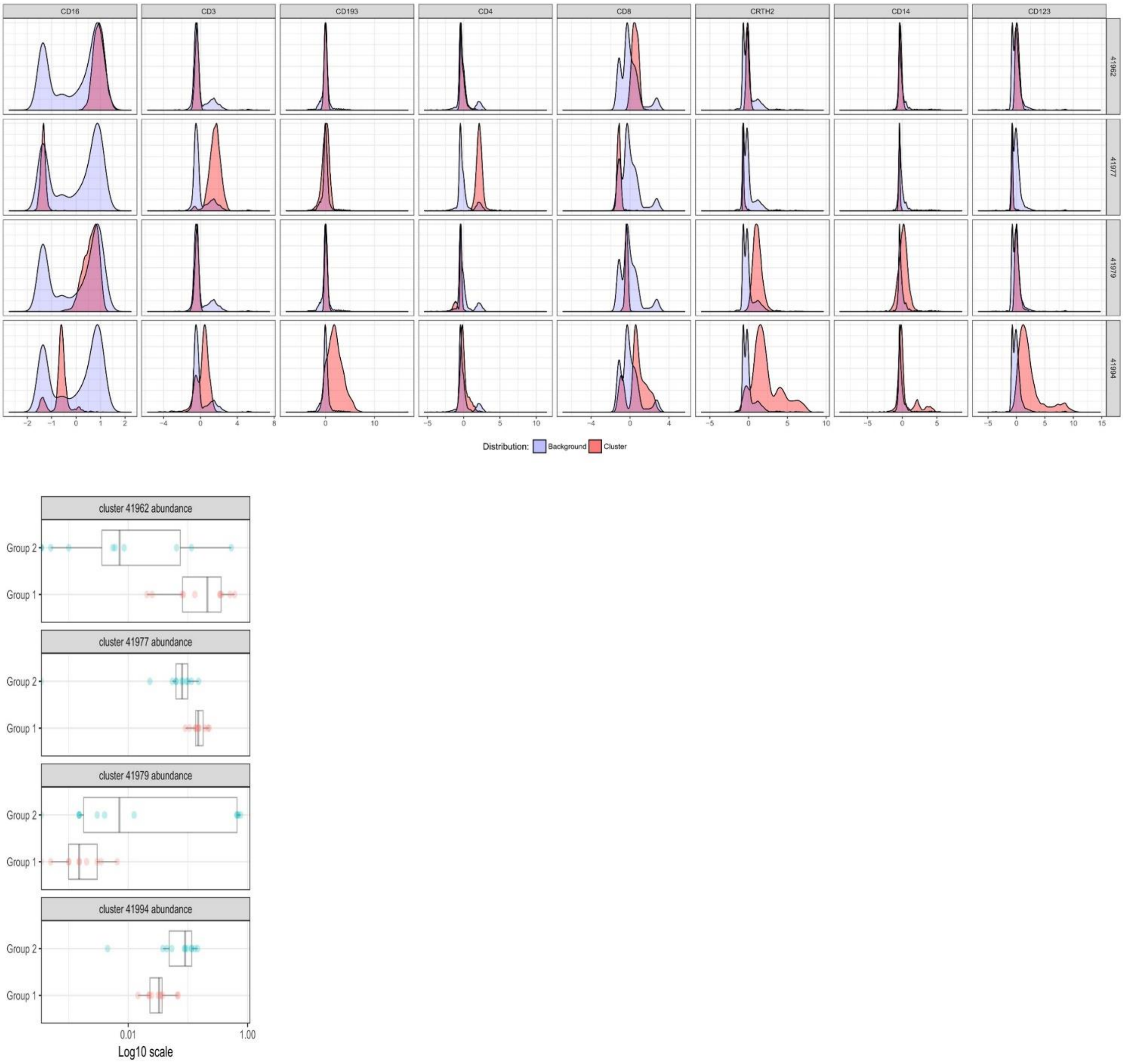

Figure S17: Upper panel: Histograms show the phenotype of the discriminant clusters, found by the model associated with smallest cross-validation rate error, corresponding to 25% misclassification. Lower panel: Differential abundance of these clusters between control (group 1) and asthma (group 2).

viSNE analysis of asthma data

viSNE was performed on the asthma data. Due to limits in computational power, the original datasets consisting of almost 2 millions cells needed to be downsampled to 3000 cells *per* individual (48000 cells in total). Figure S*18* shows the viSNE analysis performed on the arcsinh-transformed data (cofactor 5), coloured per individual (upper left panel) and for both groups (upper right). Substantial overlap of cells is present between the control and the asthmatic patients. Based on the single-marker expression profile of all cells (lower panel), we might conclude that most of the overlapping regions are associated to CD4+ T-cells; CD8+ T-cells; CD16+ cells, which might be identified as neutrophils; and CD14+ cells, which might be identified as monocytes. Also a few smaller CD193+ clusters were found, which could be eosinophils or basophils.

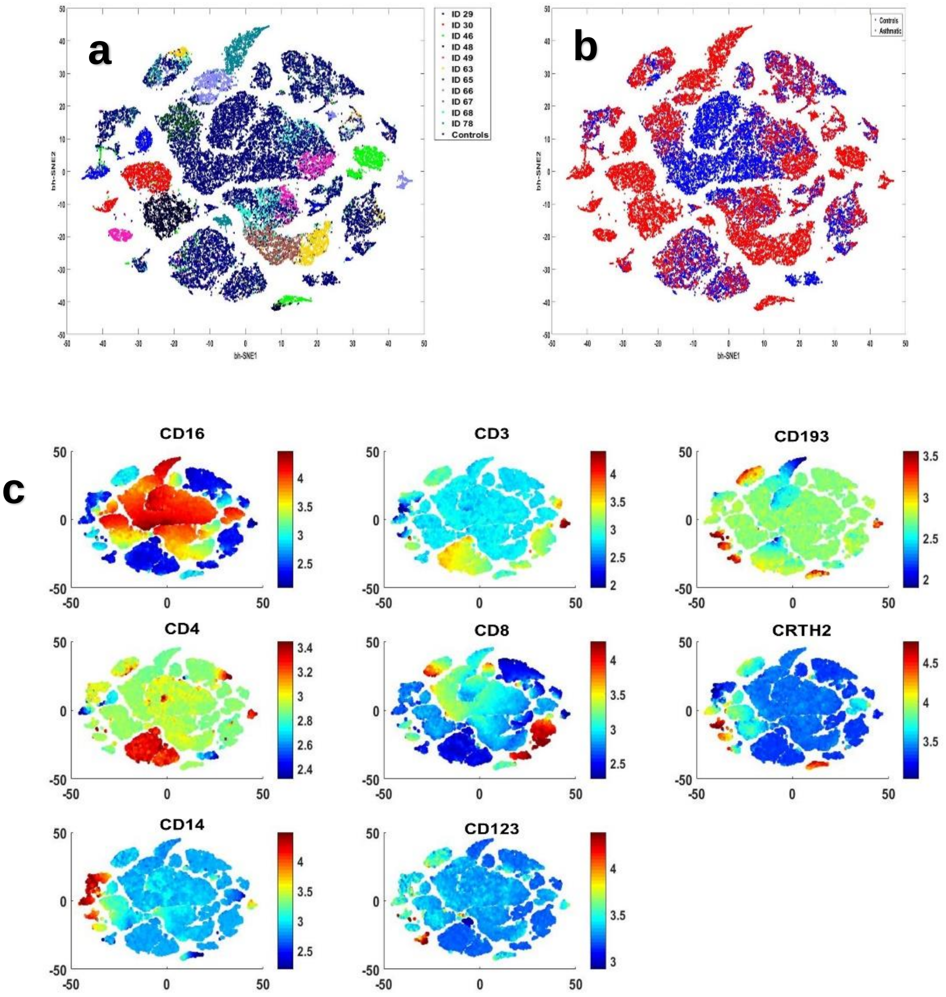

Figure S18: viSNE analysis performed on the asthma data. ***a****: cells in the viSNE map are coloured per different individual;* ***b****: cells, in the viSNE map, are coloured based on control (blue) and responder (red) group.* ***c****: cell are coloured based on the expression levels of the single 8 markers.*

Considerable heterogeneity can be observed among the asthmatic patients. In this case, maps with distributions of a single or a few patients may enhance focus on subtle and patient-specific cell populations. Figure S*19* shows the viSNE map with individuals #63 and #67, which were also grouped and analysed in a partial model by ECLIPSE (Figure 11).

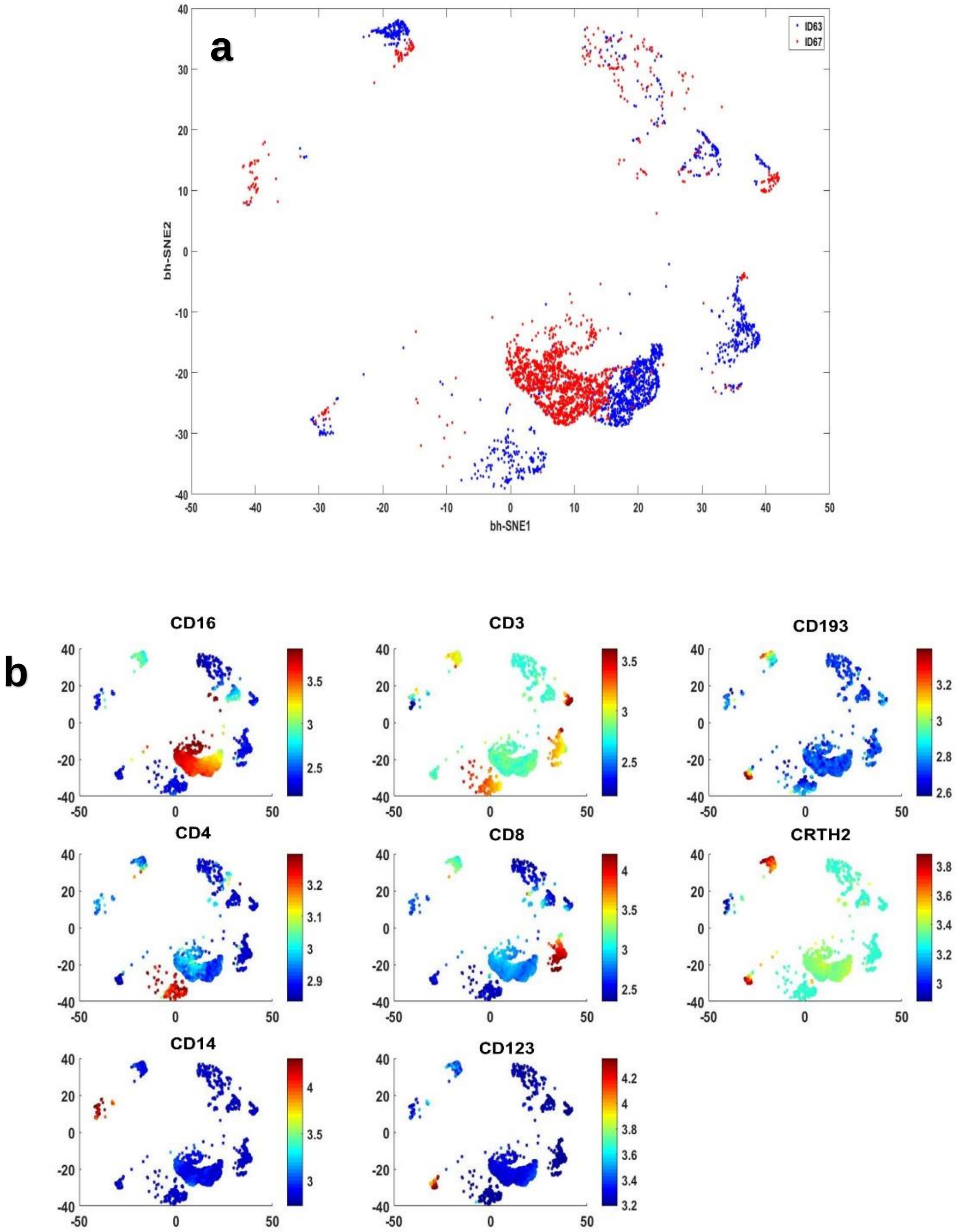

Figure S19: ***a****: viSNE map obtained by the analysis of the asthma data, only the cell distribution of asthmatic individual #63 and #67 are shown.* ***b****: cells coloured based on the expression levels of the single 8 markers.*

Similar cell subpopulations seem to be present for both individuals. However, for individual #67 only a few cells are present in various regions. This might be a consequence of the down-sampling, which retains the more abundant CD16+ cell population at the expenses of rarer cells. The phenotypical marker pattern observed in Figure S19b is similar to the phenotypes shown in the ECLIPSE partial model. CD16+ cells might be identified as neutrophils; CD123+CD193+CRTH2 cells as basophils; while CD3+CD8+ cells and CD3+CD4+ cells might be assigned to CD8+ T cells and CD4+ T cells, respectively. A key disadvantage for easy identification of the various cell populations is the representation of the marker expressions in single plots per marker.

Supplementary Material IV

Manual sequential gating of the asthma red cluster and projection into ECLIPSE space

Manual gating of the cells of the individual belonging to the red cluster (Figure 10c) was performed in FCS express 5, according to the surface markers phenotype as shown in Figure *S20*. The gating demonstrates the absence of double positive CD4/CD8 T cells. Double negative CD4/CD8 T cells are present in a very low percentage. These cells, probably also present in the control individuals, were eliminated by the ECLIPSE algorithm. We can conclude that the mutual direction of CD4 and CD8 markers in the ECLIPSE space represents a big part of the variation of the data well, associated with the presence of different types of T cells (CD4+ and CD8+).

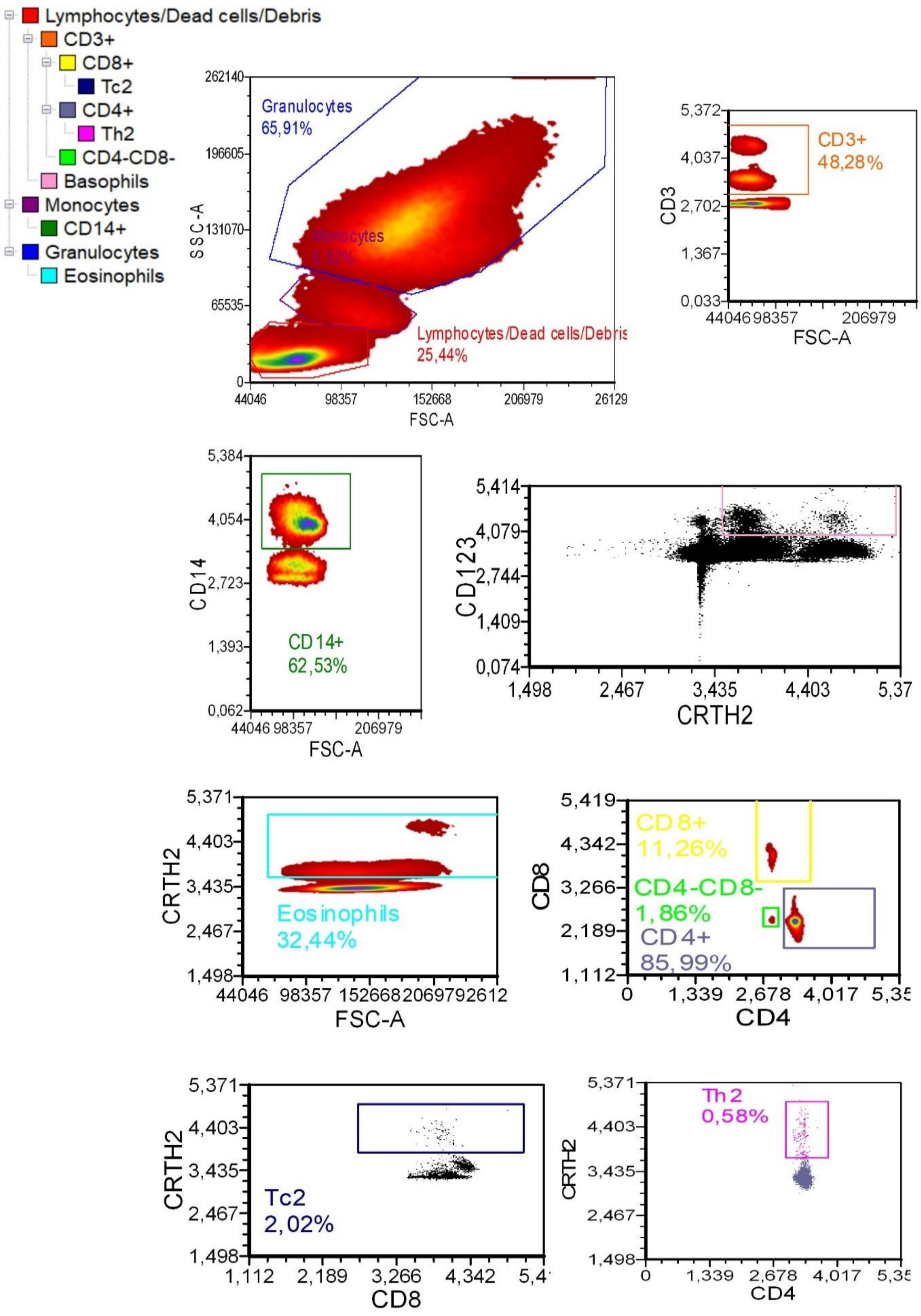

Figure S20: Manual sequential gating of the cells from the individuals belonging to the red cluster, in figure 10c. The gates are differentially coloured.

CD8+ T cells (CD3+CD8+CD4-, yellow gate) are not easily visualized in the first two PCs of the ECLIPSE model (Figure *S21,* left panel, PC1-PC2 plot). The CD8+ T cells are positioned in the middle of the plot, overlapping with other cell populations. Obviously, the other markers are more important to explain the variance of cells this 2PCs model (Figure 10C), since all the other markers show longer loadings when compared to the CD8 loading. If the CD8+ T-cells were more important, the loading would be longer and the cell population would be visible in these first 2 PCs. The investigation of the ECLIPSE space built on PC1 and PC3 made the CD8+ T cells better distinguishable (figure *S21,* right panel, PC1-PC3 plot and figure *S22*).

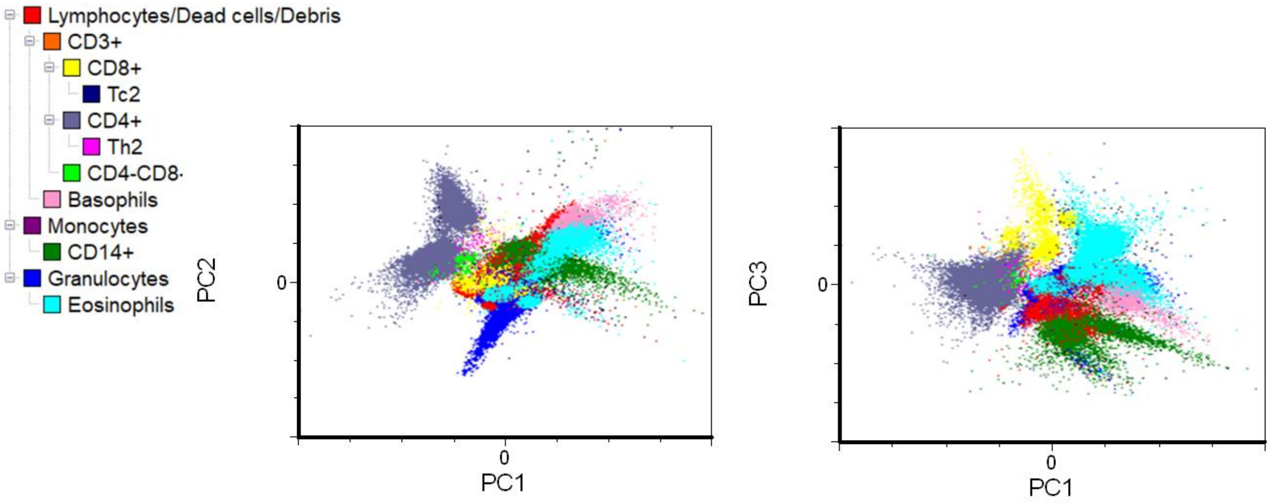

Figure S21: ECLIPSE partial model of the red cluster: the left panel shows the space built with PC1-PC2, the left panel shows the space of PC1-PC3. The cell scores in the plots are coloured accordingly to the gates found by the manual gating procedure.

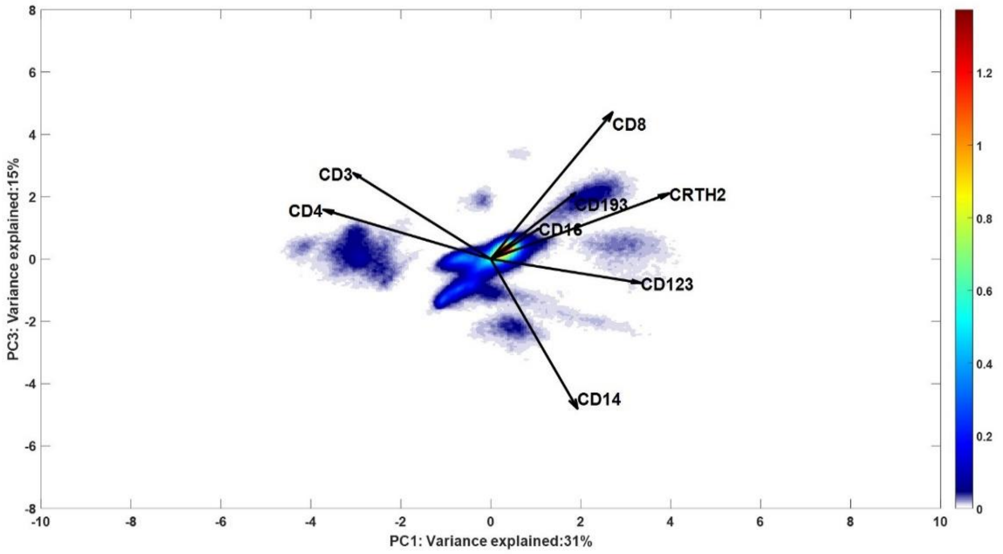

Figure S22: Density representation of the ECLIPSE partial model of the red cluster, showing PC1 and PC3.

CD4+ T-cells were easily visualized in the left part of the ECLIPSE plot (figure 10c, ECLIPSE paper), with two main distinctive populations. These populations are different because they express different levels of the marker CD3. Based on the orientation of the loadings in space spanned by PC1 and PC2, we would expect to find $CD3^{bright}$CD4+ cells situated closer to the CD3 loading; while $CD3^{dim}$CD4+ cells are expected to lie more to the left, in the direction of the CD4 loading and a bit further away from the CD3 loading. This was confirmed when backgating the cells, as shown in Figure *S23* and *S24*.

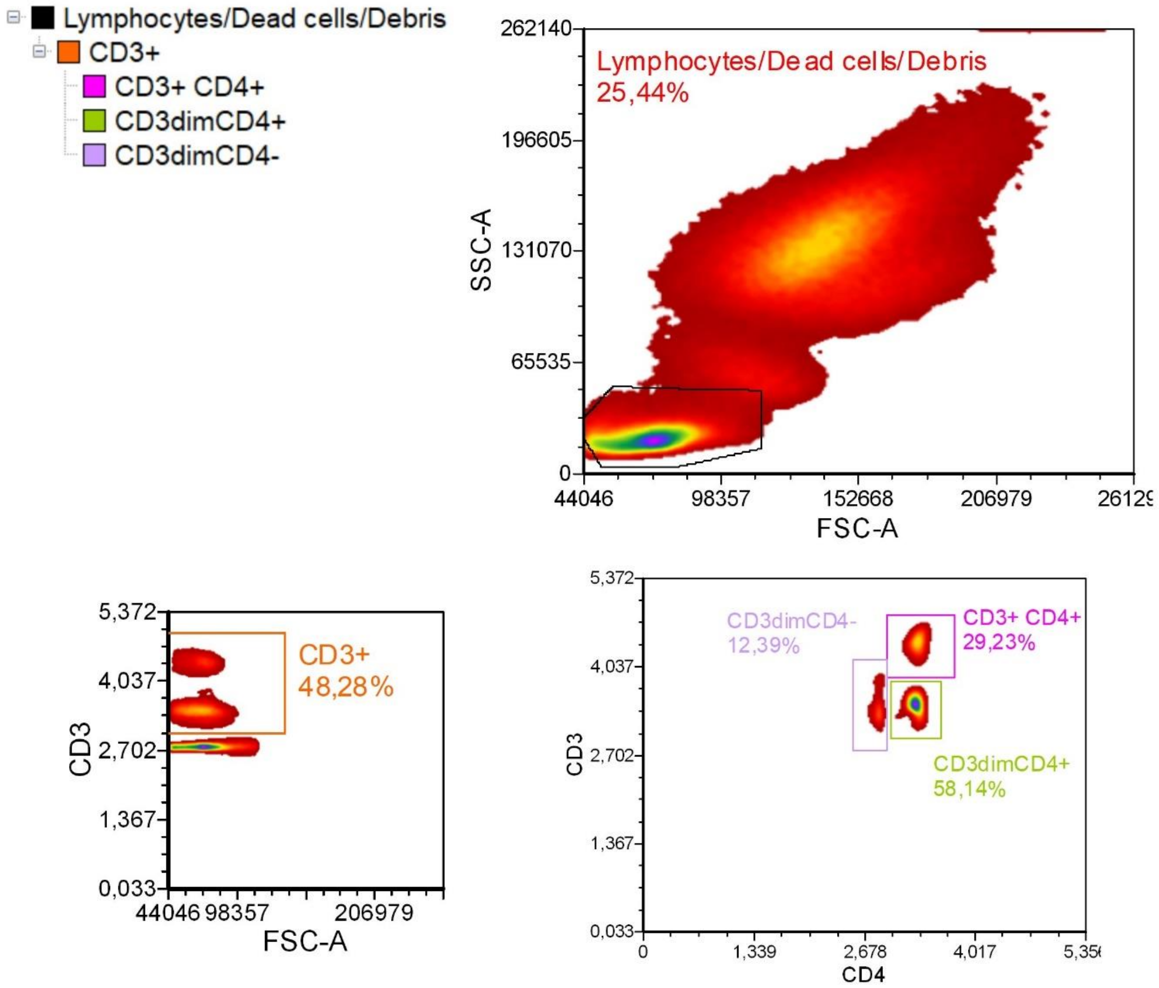

Figure S23: Manual sequential gating of the cells from the individuals belonging to the red cluster, in figure 9c. Gates for CD3+ cells are shown and coloured differently.

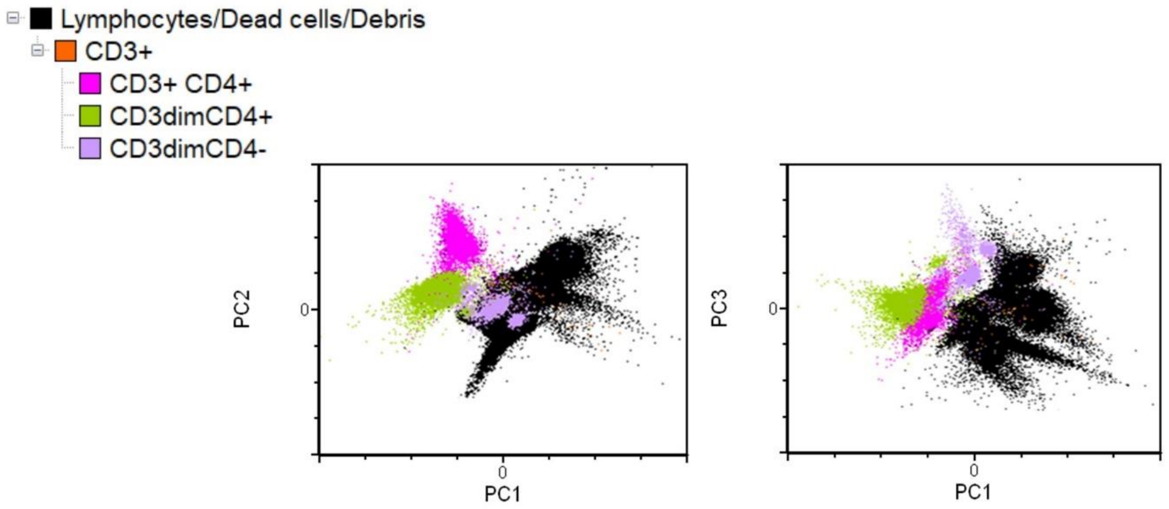

Figure S24: ECLIPSE partial model of the red cluster: the left panel shows the space built with PC1-PC2, the left panel shows the space of PC1-PC3. The cell scores in the plots are coloured accordingly to the expression of the marker CD4 and CD3, in the CD3+ gate.

Supplementary Material V

ECLIPSE and Citrus analyses on synthetic datasets

Immunological response to a stimulus or a disease can comprise changes in relative abundance of a particular cell population and/or changes in expression levels of certain markers can also lead to the appearance of new cell phenotypes. The detection of both types of changes is essential for a comprehensive understanding of the etiology of the immune response.

A two-dimensional synthetic dataset was created to show the performance of ECLIPSE and Citrus in identifying cell populations, characterized by differences in terms of abundance and expression levels of different markers. The synthetic dataset consists of 10 controls and 10 responders. The control group presents a single cell population with higher abundance than the response group. Heterogeneity has been introduced into the response group as the samples have diverse cell subpopulations, one of which consists of a small subset of 20 cells present only in one responder. This heterogeneity can be observed in the 2D scatter plot, Figure *S25* (the small cell population in yellow in the top right corner belongs to Responder 10), and in the histograms shown in Figure *S26*; contour plots showing the differing relative abundance of cell populations between controls and responders are shown in Figure *S27*.

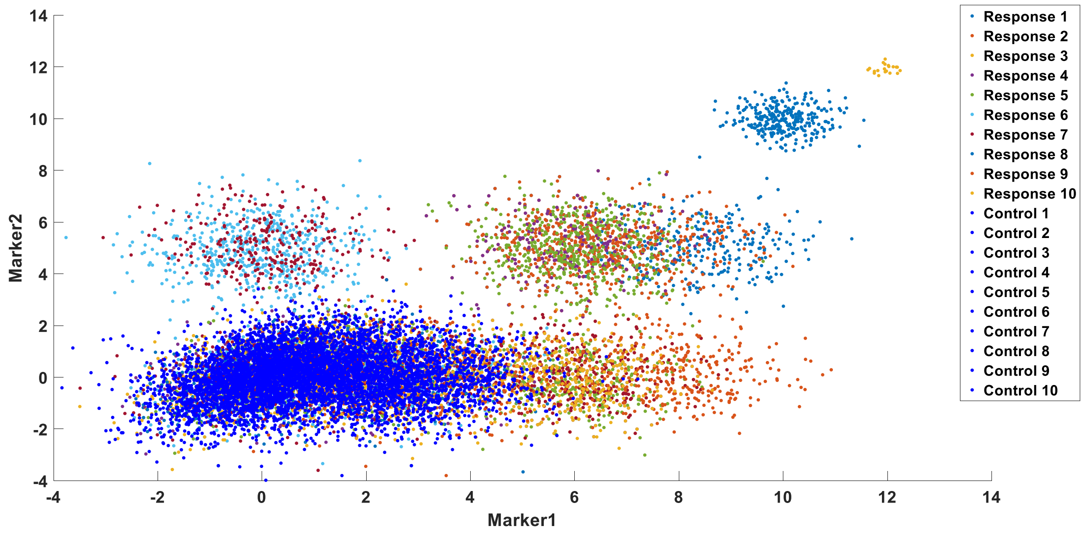

Figure S25: 2D scatter plot of the synthetic data. Cells from the control individuals are coloured in blue, while cells from the responders are differently coloured per individual.

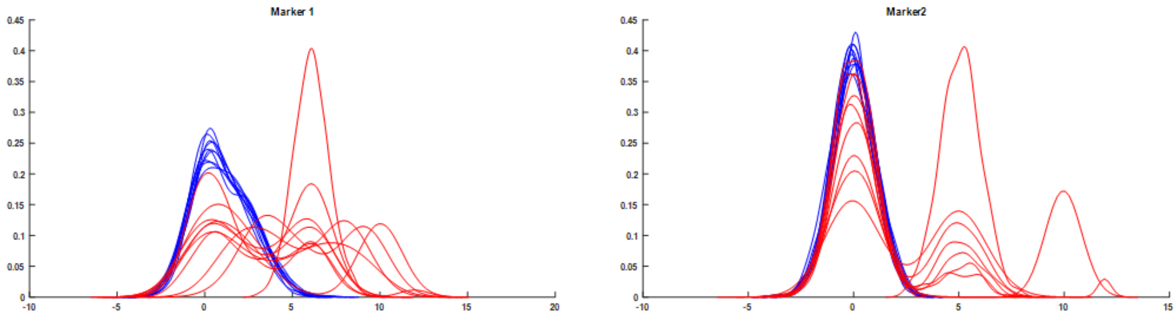

Figure S26: Histograms showing the expression of the 2 markers included in the synthetic data. Histograms of the control individuals are depicted in blue, while the histograms of the responders are in red.

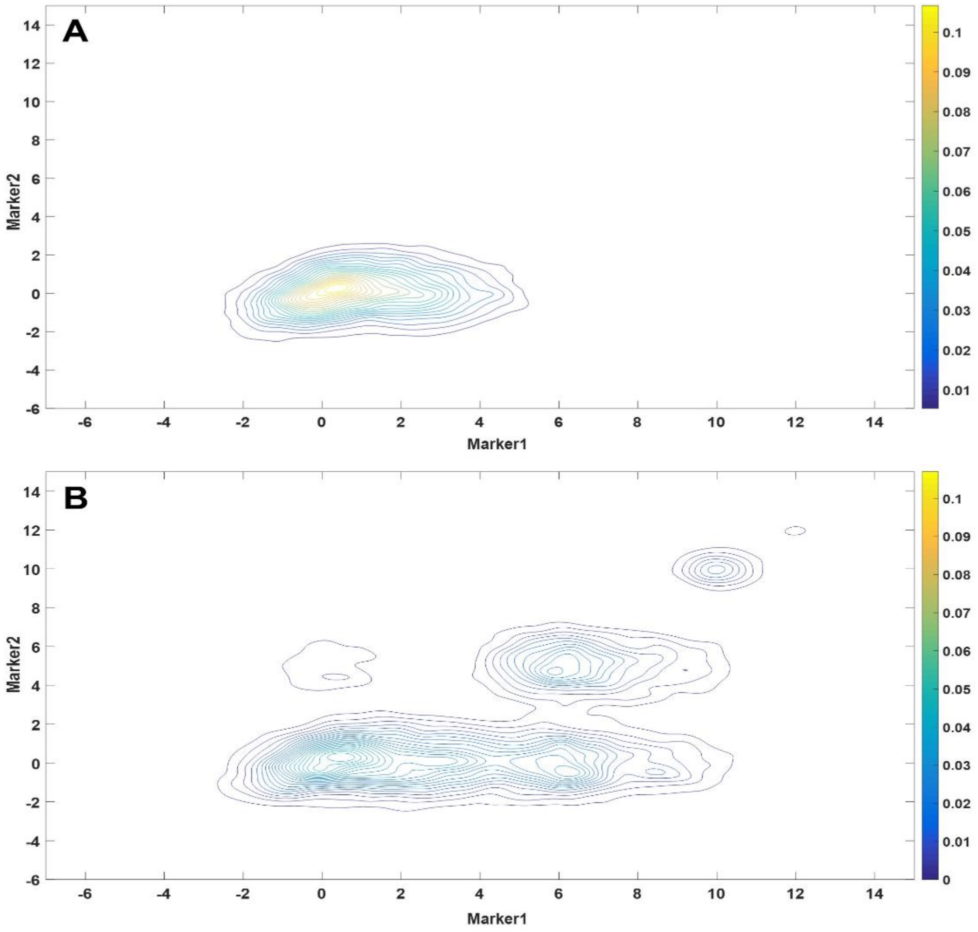

Figure S27: Panel A: contour plot of the cells distribution of control individuals. A single cell population is present for all the controls; Panel B: contour plot of cells distribution for response individuals.

Citrus was applied to the dataset with a Minimum Cluster Size Threshold (MCST) corresponding to 10 cells, so that the method could identify the rare subset. The accuracy of the constructed models is shown in Figure *S28*, which reports the model cross-validation error rate versus the regularization threshold associated to the number of features identified. Seven features were detected by the model with the minimum error (cv.min), while the model with a cross validation error 1 std higher than the minimum (cv.1se) identified two most discriminant features.

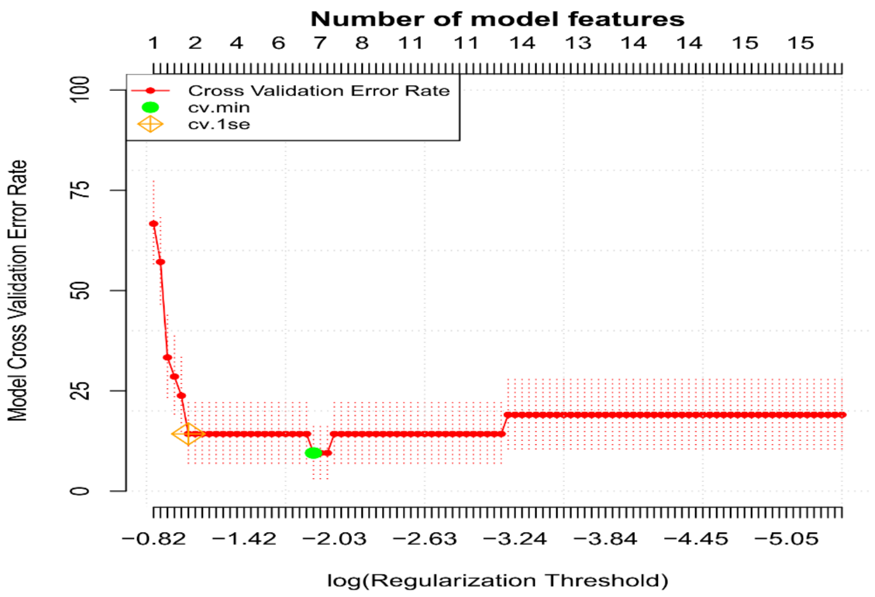

Figure S28: The figure shows the Model Cross-validation Error Rate vs the log(Regularization Threshold) for the classification models built on the simulated data. The green circle (cv.min) points out the model with the smallest number of features necessary to obtain the lowest cross-validation error, which corresponds to 10% of misclassified samples; the orange diamond (cv_1se) indicates the model with the smallest number of features associated to cross-validation error 1 std higher than the minimum error, which leads to an error of around 15%.

The features returned by both models are visualized in Figure *S29*. In both cases, the relative abundance of the identified cell clusters is higher in the control group compared to the response. These clusters correspond to the population with lower expressions of *Marker1* and *Marker2*, created as more abundant for the control samples. Although the information of differential cell population abundance might be relevant for describing an immune response, evidence about such relevant response-specific cell populations is missing from the optimally parsimonious Citrus model.

a

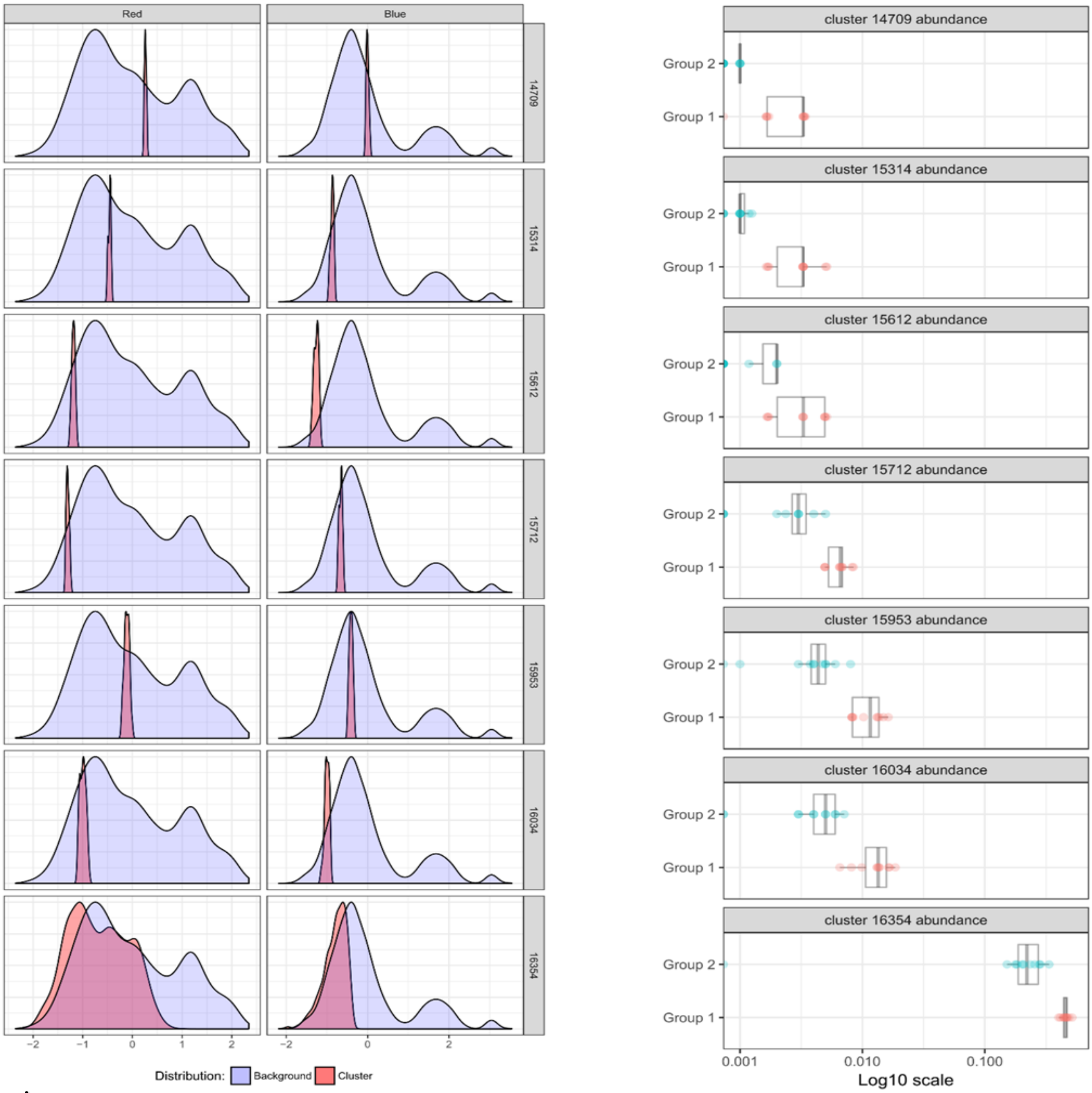

b

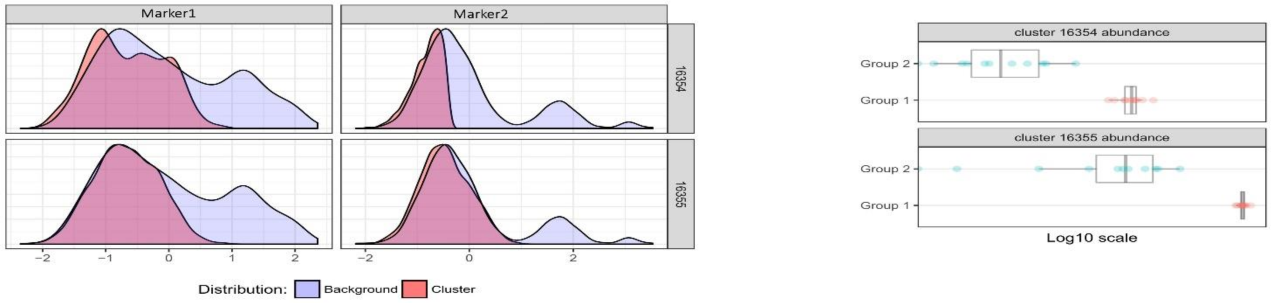

Figure S29: Panel A: Histograms show the phenotype of the discriminant clusters, found by the model associated with cv.min error (10% of misclassification). Differential abundance of these clusters between control (group 1) and response group (group 2) can be observed in the right panel: these are all found more abundant in the control group. Panel B: Histograms show the phenotype of the discriminant clusters, found by the model associated with cv.1se error (15% of misclassification). Differential abundance of these clusters between control (group 1) and response group (group 2) can be observed in the right panel: they are more abundant in the control group.

Secondly, we performed an ECLIPSE analysis on the synthetic dataset. The Difference between Densities (DbD) plot (Figure *S30*) clearly shows the difference between the two groups. Advantageous is the possibility to estimate the difference between densities of a single responder against the control group estimate (Figure *S30*B). This will enhance the resolution on a specific individual and it is helpful when one sample is available for the response class/classes.

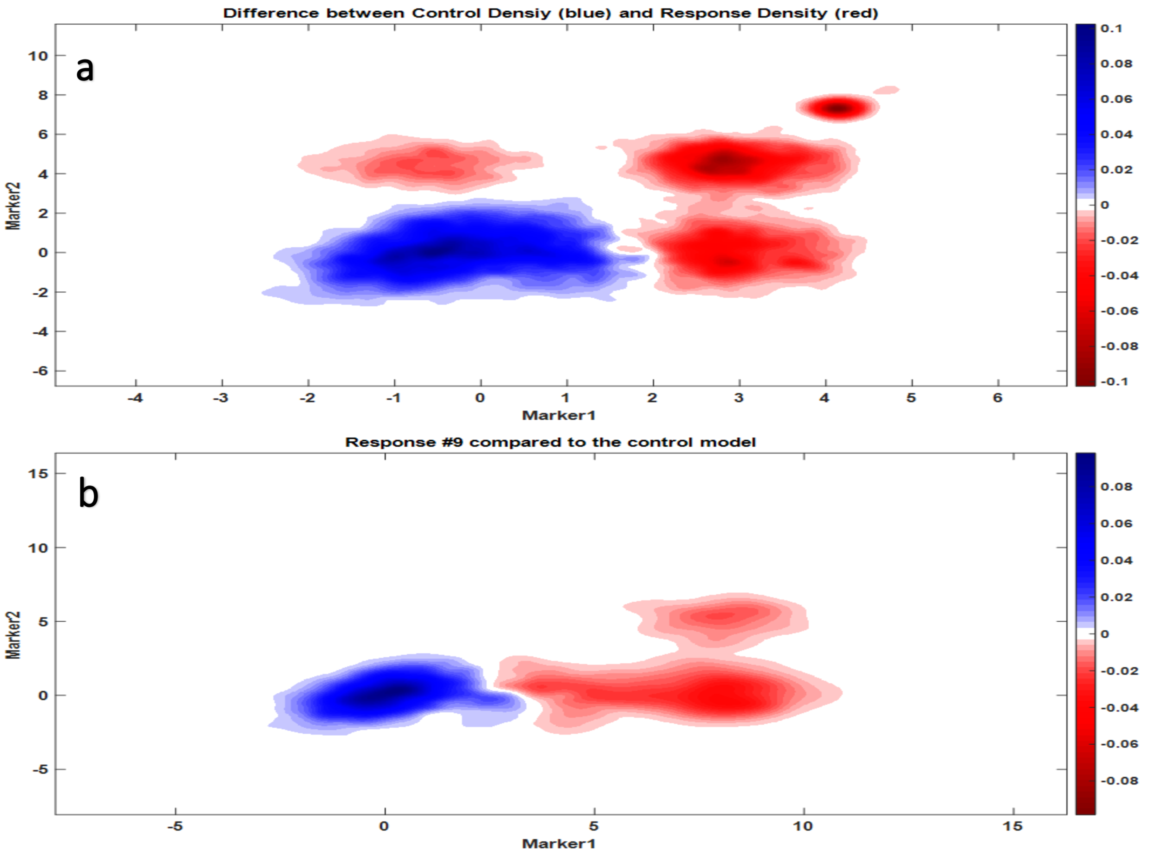

*Figure S30: Panel **a**: Difference between Densities plot, obtained by subtracting the KDE cell distribution of 10 responders from the cumulative KDE of the control cells distribution. The negative intensity (red) indicates the location where cells over-produced in the responder are more likely to be present; the positive intensity (blue) indicates the location where control cells are more likely to be present. Panel **b**: Difference between Densities plot of the KDE of cell distribution of a responder (ID #9) subtracted from the cumulative KDE of the control cells distribution.*

Response cells overlapping with the control marker variability were removed from the dataset and the remaining cells are displayed in Figure *S31*. The small population existing of 20 cells is still visualized (top right corner). Few cells are retained also for the control group, due to the individual variability we introduced between the control samples.

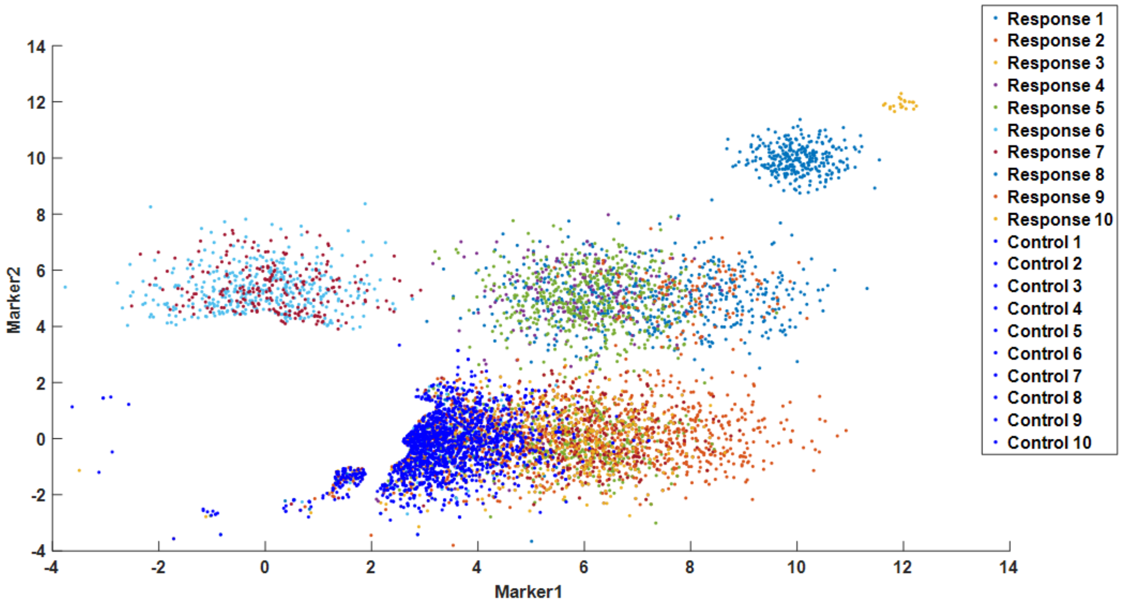

Figure S31: 2D scatter plot of the synthetic data, after the elimination of normal cells performed by ECLIPSE. Cells left for the control individuals, due to personal variability, are coloured in blue, while cells from the responders are differently coloured per individual.

The results of the analyses on the synthetic data showed how ECLIPSE outperforms Citrus in presence of a heterogeneity within the response class. In fact, Citrus is a two-class classifier method which needs numerous samples with similar phenotypically properties within both groups to find their signature features. Contrarily, ECLIPSE is a one class classifier method that requires only a group of control individuals. This is necessary to define a reference, against which a single patient or a group of patients, even highly heterogeneous in their response, can be compared. The removal of normal cells will put more focus on the response specific subpopulations of each individual, including rare cell subsets.

In this example, the dimensional reduction step of ECLIPSE was not needed because the data had only two dimensions. In order to show that the SCA-based transformation will not affect the discovery of cells subpopulations we performed the analyses on a 3D datasets, obtained from the first one after specific transformations.

A third random aspect was added to the first dataset; the matrix obtained was rotated by using orthogonal Procrustes rotation[8], which allowed to introduce a more realistic correlation across all the three variables. A 3D scatter plot of the new dataset and histograms of the three marker expression levels are shown below (Figure *S32* and *S33*).

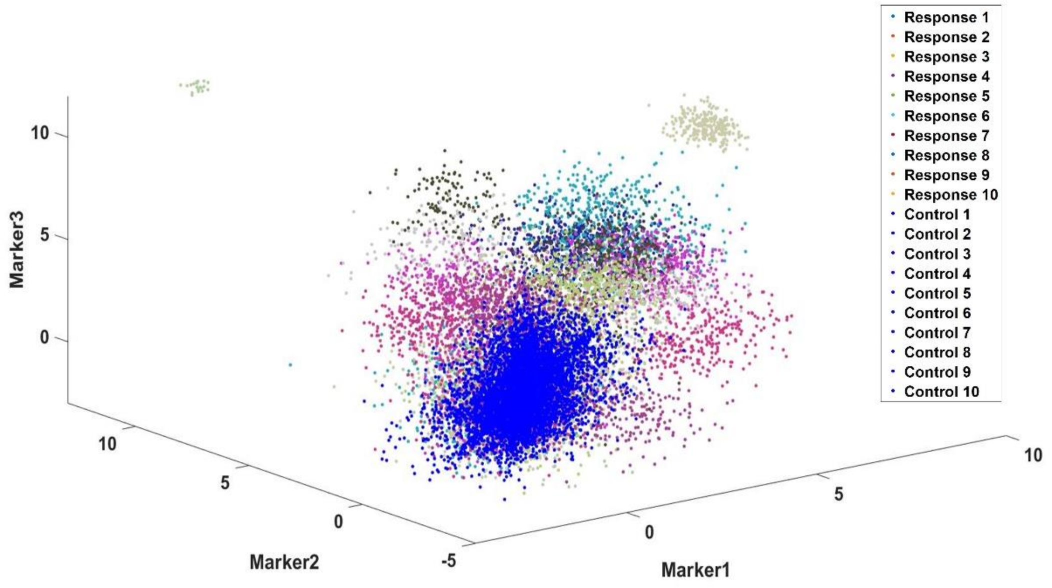

Figure S32: 3D scatter plot of the 3 dimensional synthetic data. Cells from the control individuals are coloured in blue, while cells from the responders are differently coloured per individual.

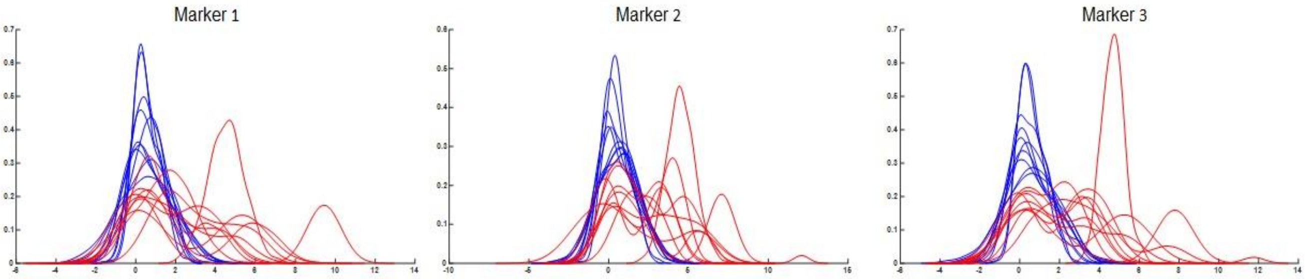

Figure S33: Histograms show the marker expression of the three markers created for the synthetic data. Distributions from control individuals is displayed in blue, while distributions from response are in red.

We applied the Citrus method on this new dataset. The cross-validated error rates of the model obtained, in Figure *S34*, indicate the model with only one feature as optimal. In this case, different relative abundance of cell populations is not identified as relevant feature (Figure *S35*). As for the previous Citrus analysis, the rare cell subset is not detected.

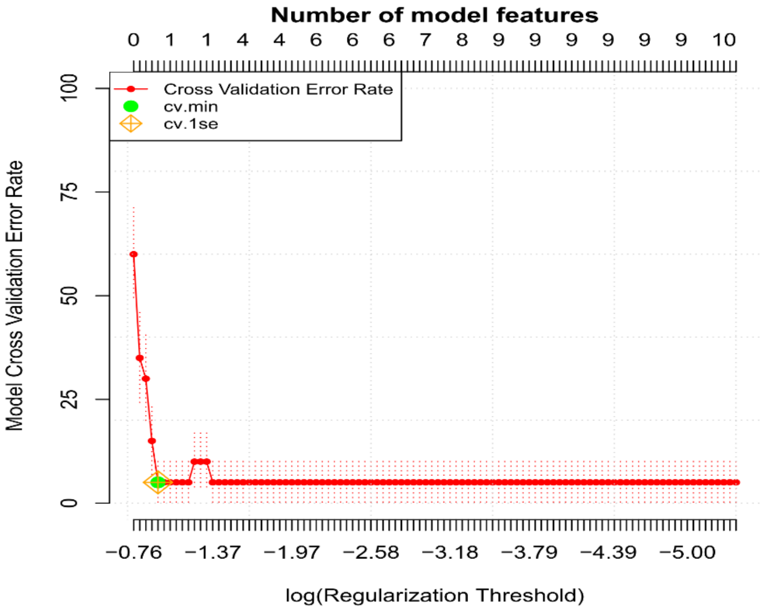

Figure S34: The figure shows the Model Cross-validation Error Rate vs the log(Regularization Threshold) for the classification models constructed on the synthetic dataset. The lowest cross-validation error (cv.min and cv.1se) is obtained with the model using one feature. This corresponds to an error rate of around 2.5%.

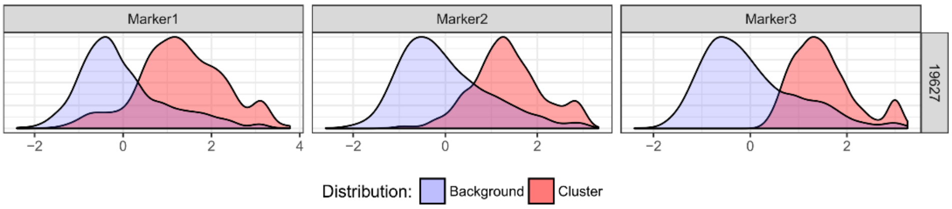

Figure S35: The histogram shows the phenotype of the cells belonging to the cluster (red) selected by the cross-validated model and found more abundant in the responder. The background histograms (blue) shows the rest of the data, not included in the cluster.

ECLIPSE analysis was performed on the three-dimensional dataset, whose dimensionality was reduced with Simultaneous Component Analysis to two components. Figure *S36* shows the Control Model with the density estimation of cell score distributions for the control (blue) and response group (red), together with the marker loadings. The axis show the variance explained by the model. The deviation of the responders (Figure *S36*B) from the reference group principally occurs along the first component PC1 and is mainly described by Marker1. Marker2 and Marker3 mostly explain the specific variability of cells along the second component PC2.

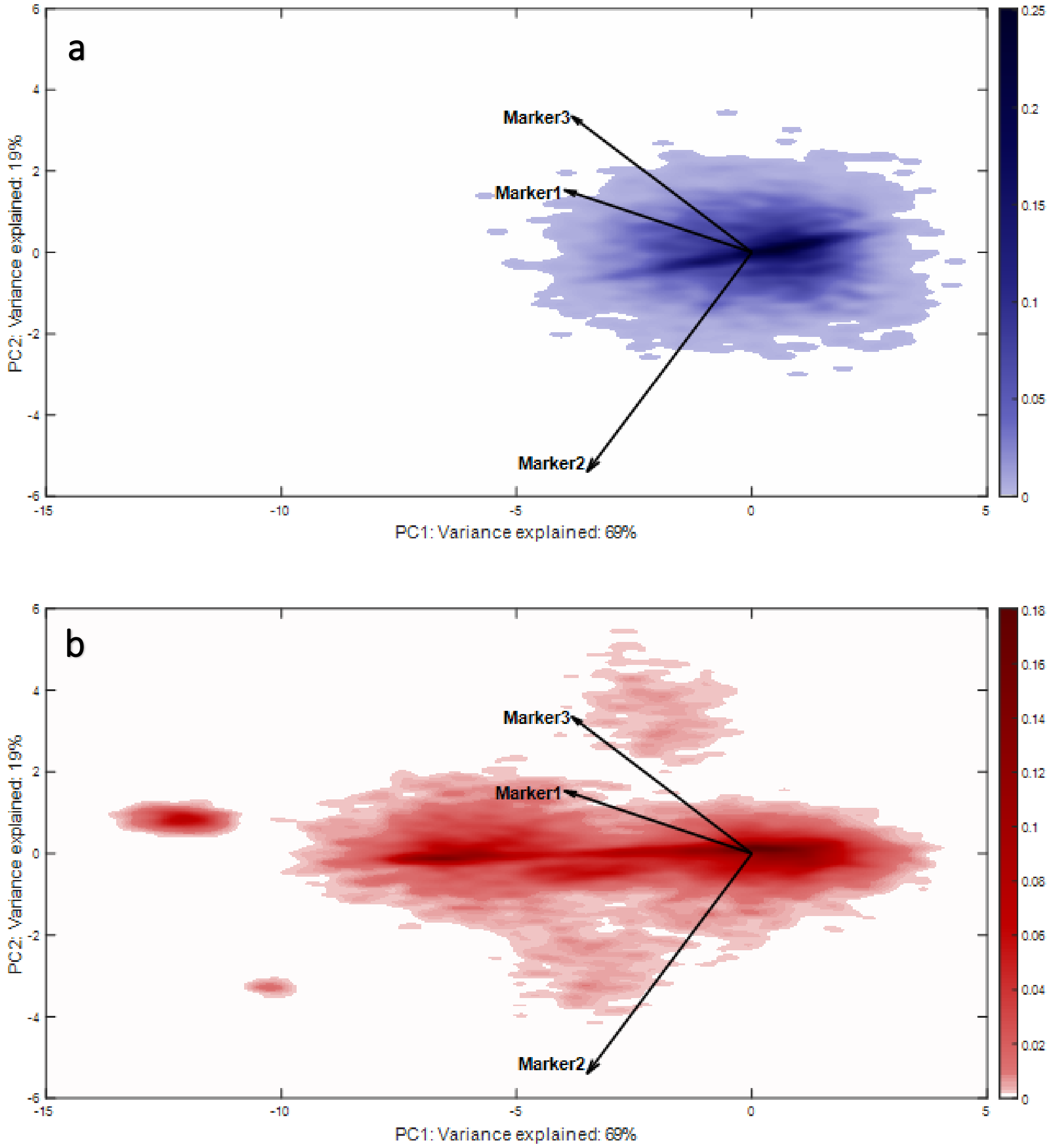

Figure S36: Panel A: shows the density estimate of the control cells scores distribution. Panel B: shows the KDE estimates of the response cells scores distribution. Biplots of the KDE estimates in the Control Model, built on the variability of the control individuals. The loadings of Control Model are plotted as vectors: their length indicates the contribution of each marker to the cell-to-cell variability; the mutual directions suggest a positive (same direction) or negative (opposite direction) co-expression.

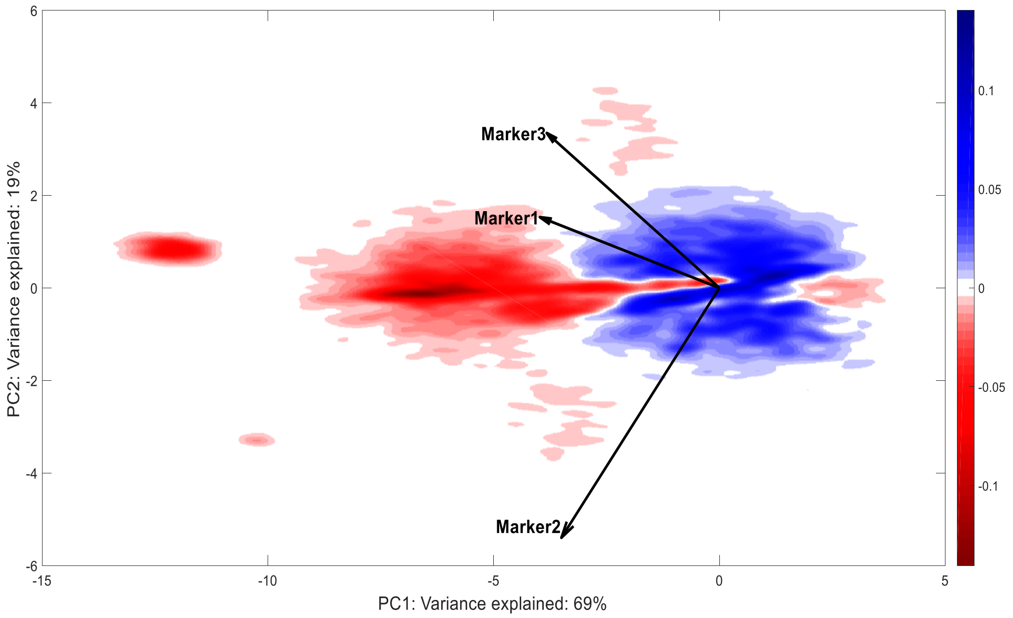

Figure S37: Density estimate of the cells scores distribution from the responder individuals is subtracted from the density estimate of the control individuals. The negative intensity (red) specifies the location where cells over-produced in the responder are more likely to be present, while the positive intensity (blue) indicates the location where healthy cells are more likely to be present. The white area between the red and blue areas corresponds to a value of KDE=0, which can indicate bins with no cells or equal intensity of control and responder estimates.

Responder cells with a marker profile variability overlapping with the control variability were removed. A new SCA-based space is built on the variability of cells left after this removal and it specifically focuses only on the marker expression specific for the responder cells. The resulting ECLIPSE space is shown in Figure *S38*, which does show the rare cell population in down right corner. Moreover, the loadings show the correct co-expression between Marker 2 and 3 for this rare cell population which was masked in Figure *S37*, due to the high variability in the control cells of all markers.

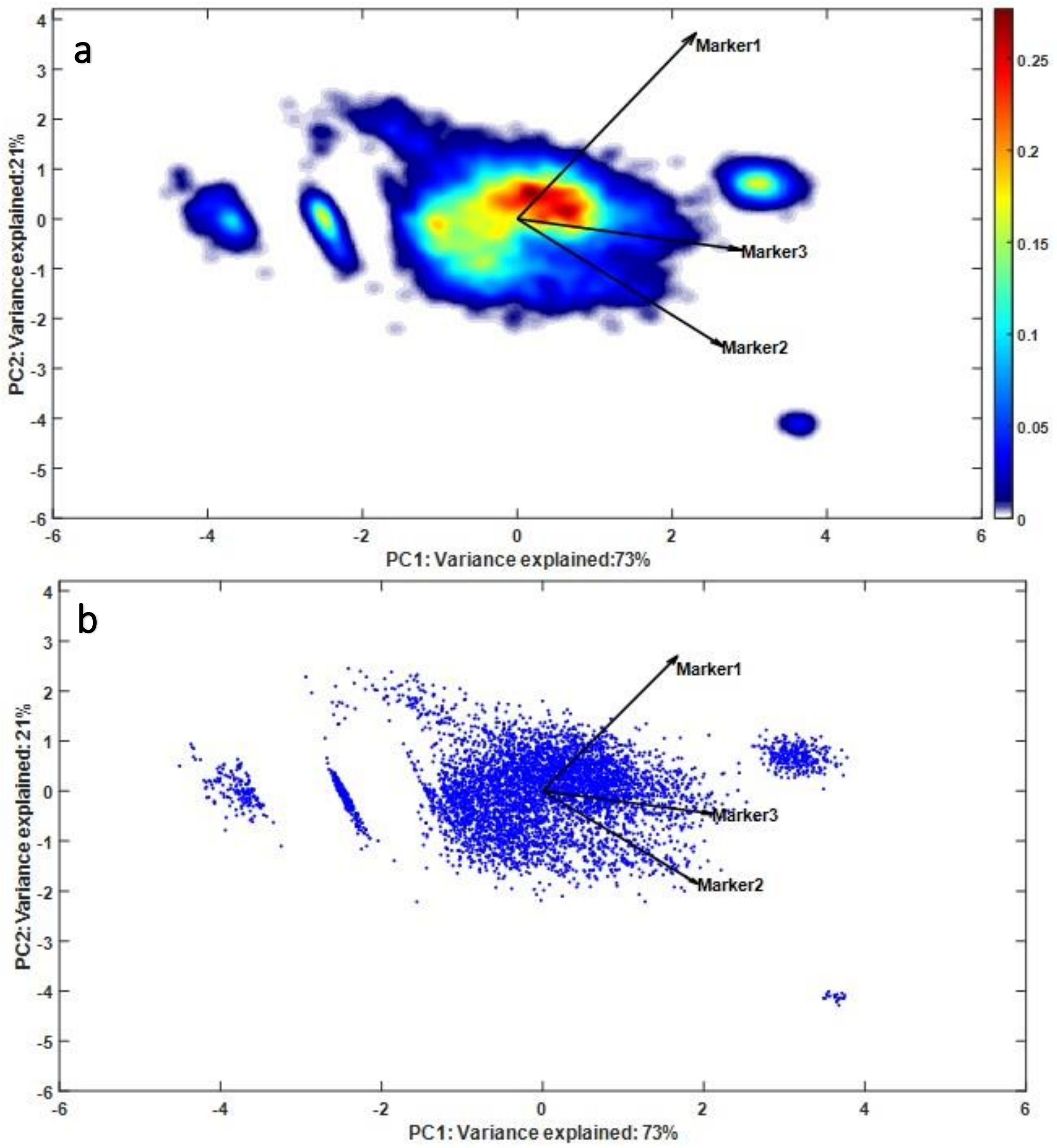

*Figure S38: ECLIPSE model built on the cells of the responder individuals, after removal of normal cells (KDE representation, panel **a**; single cells representation panel **b**). The loadings show the marker co-expression specific for the response-specific cell subsets. The small population, consisting of 20 cells, is visible on the bottom right corner.*

References

1 Engel, J. *et al.* Breaking with trends in pre-processing? *TrAC Trends in Analytical Chemistry* **50**, 96-106, doi:https://doi.org/10.1016/j.trac.2013.04.015 (2013).

2 Roederer, M. Compensation in flow cytometry. *Current protocols in cytometry* **Chapter 1**, Unit 1.14, doi:10.1002/0471142956.cy0114s22 (2002).

3 Finak, G., Perez, J. M., Weng, A. & Gottardo, R. Optimizing transformations for automated, high throughput analysis of flow cytometry data. *BMC bioinformatics* **11**, 546, doi:10.1186/1471-2105-11-546 (2010).

4 Timmerman, M. E., Hoefsloot, H. C. J., Smilde, A. K. & Ceulemans, E. Scaling in ANOVA-simultaneous component analysis. *Metabolomics* **11**, 1265-1276, doi:10.1007/s11306-015-0785-8 (2015).

5 Bro, R. & Smilde, A. K. Centering and scaling in component analysis. *Journal of Chemometrics* **17**, 16-33, doi:10.1002/cem.773 (2003).

6 Bruggner, R. V., Bodenmiller, B., Dill, D. L., Tibshirani, R. J. & Nolan, G. P. Automated identification of stratifying signatures in cellular subpopulations. *Proceedings of the National Academy of Sciences of the United States of America* **111**, E2770-E2777, doi:10.1073/pnas.1408792111 (2014).

7 Amir el, A. D. *et al.* viSNE enables visualization of high dimensional single-cell data and reveals phenotypic heterogeneity of leukemia. *Nature biotechnology* **31**, 545-552, doi:10.1038/nbt.2594 (2013).

8 Ten Berge, J. M. F. Orthogonal procrustes rotation for two or more matrices. *Psychometrika* **42**, 267-276, doi:10.1007/bf02294053 (1977).
